# Inequalities in all-cause mortality by ethnicity in the United Kingdom: a systematic review and meta-analysis

**DOI:** 10.1016/j.lanepe.2025.101498

**Published:** 2025-10-21

**Authors:** Fiona F. Stanaway, Saman Khalatbari-Soltani, Lin Zhu, Thulashigan Sreeharan, Naomi Noguchi, Anupa Pathak, Erin Mathieu, Raj Bhopal

**Affiliations:** a Sydney School of Public Health, Faculty of Medicine and Health, The University of Sydney, Sydney, 2006, Australia; b Usher Institute, University of Edinburgh, 5‒7 Little France Road, Edinburgh, EH16 4UX, Scotland

**Keywords:** Ethnicity, Mortality, Inequality, Equity

## Abstract

**Background:**

Ethnic inequalities in health outcomes are a persistent concern in the United Kingdom (UK) and internationally. In this systematic review and meta-analysis, we aimed to quantify ethnic differences in all-cause mortality across the UK and explore the potential modifying roles of socioeconomic position (SEP) and country of birth.

**Methods:**

We searched Medline, Embase, Scopus, and Web of Science from inception to April 20, 2024, and identified grey literature via EThOS, ProQuest, Google, and data-sharing platforms. Eligible studies reported all-cause mortality by ethnicity or other commonly used measures such as country of birth or person or parents and surname in UK populations. Two reviewers independently conducted screening, data extraction, and risk of bias assessment using the ROBINS-E tool. Certainty of evidence was evaluated using GRADE. The review was registered with PROSPERO (CRD42019146143).

**Findings:**

Of 7672 records screened, 32 studies met inclusion criteria, supplemented by unpublished data from three cohort studies. Age-adjusted all-cause mortality was consistently lower among females of Bangladeshi (Relative Risk = 0.80, 95% CI 0.67–0.95), Indian (0.78, 0.68–0.88), Pakistani (0.85, 0.79–0.91), Black Caribbean (0.83, 0.81–0.85), Chinese (0.64, 0.61–0.69), and Mixed ethnicity (0.94, 0.90–0.98), and among males of Bangladeshi (0.89, 0.86–0.93), Indian (0.78, 0.69–0.88), Pakistani (0.76, 0.69–0.83), Black Caribbean (0.89, 0.85–0.93), and Chinese ethnicity (0.66, 0.50–0.87)), compared with White British individuals. In contrast, White Irish (1.20, 1.06–1.34) and White Scottish males (1.19, 1.11–1.27), and White Scottish females (1.18, 1.08–1.29), had higher mortality than other White British. Evidence on the role of SEP was inconclusive due to methodological heterogeneity. Evidence on the role of country of birth was also limited with wide and overlapping confidence intervals around the results stratified by country of birth (UK-born vs non-UK-born) in each ethnic group. However, the UK-born Black Caribbean population had higher relative mortality rate compared to the UK-born White British population (1.49; 0.98–2.28), although this did not quite reach statistical significance.

**Interpretation:**

Inequalities in mortality by ethnic group were consistent and substantial. The contribution of SEP to observed inequalities is complex and the quality of evidence is impacted by methodological heterogeneity. However, SEP disadvantage is unlikely to explain the lower relative mortality of non-white groups. The potential for inequalities within ethnic groups by country of birth has important policy implications, as this suggests the need to tailor public health strategies within ethnic groups by country of birth. However, more evidence is needed for most ethnic groups. As a result, we call for greater reporting of both ethnicity and country of birth in ethnic inequalities research in the UK.

**Funding:**

Australian Research Council Centre of Excellence in Population Ageing Research (Grant ID: CE170100PRO005).

**Research in context**

**Evidence before this study**

Inequalities in all-cause mortality in the United Kingdom (UK) have been reported on for several decades. There is important heterogeneity in study methodology and estimates of inequalities and few studies have examined differences within ethnic groups by country of birth. To date, no systematic review or meta-analysis has comprehensively assessed ethnic inequalities in mortality in the UK. We conducted a systematic review and meta-analysis of studies published in Medline, Embase, Scopus, and Web of Science from inception to April 20, 2024. We updated our search in Medline and Embase on August 13, 2025 and found no further published studies meeting our inclusion criteria. Grey literature was searched via EThOS, ProQuest, and Google, and we sought unpublished cohort data through data-sharing platforms. While unlinked Census and death registry studies were generally at high risk of bias, most included cohort studies were assessed as having low risk of bias. The GRADE certainty of evidence for mortality inequalities was moderate for most ethnic groups when analyses were restricted to cohort studies with low or some risk of bias.

**Added value of this study**

This is to the best of our knowledge the first comprehensive systematic review and meta-analysis to quantify ethnic inequalities in all-cause mortality in the UK. We also examined the contribution of socioeconomic position (SEP) and country of birth to observed inequalities. Evidence on the influence of SEP was limited by methodological heterogeneity. Evidence for differences within ethnic groups by country of birth lacked precision due to the small number of contributing studies. However, UK-born Black Caribbean individuals had higher relative mortality than their UK-born White British counterparts (RR 1.49; 95% CI 0.98–2.28), although this did not quite reach statistical significance.

**Implications of all the available evidence**

This meta-analysis provides robust and quantifiable evidence of ethnic inequalities in mortality in the UK. It also highlights a critical gap in the literature: the lack of data disaggregated by country of birth within ethnic groups. The elevated mortality observed among UK-born Black Caribbean individuals underscores the importance of this distinction. There is also emerging evidence of important differences in health between migrants and their descendants in other European countries. The magnitude and consistency of the observed inequalities in mortality underscore their public health significance and the urgent need for targeted policy and research responses.

## Introduction

Ethnicity is a multi-dimensional concept that is challenging and complex to define as it is fluid in nature and varies by time,^1^ place and context,^2^ and is impacted by societal group relations.^3^ However, most definitions include a sense of identification with a particular group that can be based on shared culture and traditions, religion, language, and ancestral origins.^4^^,^^5^ Growing ethnic diversity in the United Kingdom (UK)^6^ and substantial inequalities in COVID-19 mortality by ethnicity^7^ exemplify the importance of high-quality evidence on the presence of ethnic health inequalities. Moreover, this evidence needs to be provided by granular or detailed ethnicity categories, given the diversity within broad categories such as Black, Asian and Minority Ethnic (BAME).^8^, ^9^, ^10^ Potential differences in health within ethnic minority groups by country of birth are also important as the size of the UK-born population of many ethnic groups is now substantial and UK-born ethnic minority individuals do not benefit from the healthy migrant effect.^11^

All-cause mortality is an important measure of overall health status, with several studies in the UK having published on mortality differences by ethnicity and migration status.^12^, ^13^, ^14^, ^15^, ^16^, ^17^, ^18^ However, to date, there has been no systematic review and meta-analysis that has produced a summary of this evidence. Furthermore, contributors to these mortality inequalities, including socioeconomic position (SEP) and differences within ethnic groups by country of birth, have not been well characterised. Our objective is to quantify mortality inequalities between major ethnic minority groups and the White British majority population in the UK, examining important contributors to these inequalities such as country of birth, SEP and risk of bias, and summarising important gaps in the evidence-base.

## Methods

This systematic review utilises the Preferred Reporting Items for Systematic Reviews and Meta-Analysis statement^19^ and checklist (see Supplementary File, S1) with methods based on the Cochrane Handbook of systematic reviews.^20^ The protocol was prospectively registered with PROSPERO (CRD42019146143), peer-reviewed and published.^21^

### Eligibility criteria

We included studies with population-based samples from any country or region within the UK. We included studies that measured ethnicity based on self-report, in addition to those using researcher/observer assigned ethnicity, or other proxy measures such as country of birth. We excluded studies that grouped several heterogeneous groups together, such as non-White or BAME. The list of ethnic groups considered is available in our published protocol.^21^ We included studies with a relevant majority comparator population, including White British, White Scottish, White Irish, White or rest of the population/total population. Included studies needed to provide data comparing all-cause mortality by ethnicity, presented using standardised mortality ratio (SMR), relative risk (RR), hazard ratio (HR), odds ratio (OR) or other equivalent relative measure. We included studies providing mortality data by different causes of death (e.g. COVID, non-COVID), if the data could be combined to provide a measure of all-cause mortality. Similarly, we included studies reporting age-standardised mortality rates by ethnicity if relative measures could be estimated from the data provided. Finally, where results were only available in graphs, we used the online software WebPlotDigitizer^22^ to extract results. We included all study types that provided mortality data by ethnicity, namely: unlinked studies using Census denominators and death-registry mortality incidence; and prospective and retrospective cohort studies (including those based on Census linkage or linkage of administrative data from primary care services).

### Data sources

We searched: Medline (OvidSP), Embase (OvidSP), Scopus and Web of Science. We conducted additional searches of grey literature via EThOS (the British Library e-theses online service) and Proquest. Searches were carried out from inception to 20/04/2024 with no language restrictions. The initial search was carried out on 02/08/2019, and we repeated the search on 22/10/2021, 27/03/2023 and 20/04/2024. The Medline search strategy is published^21^ and search strategies for all databases are in the Supplementary File (S2). We searched Google for preprint publications and government reports. We carried out forward and backward citation tracking of identified relevant articles. We also contacted experts in the field for additional studies that we may have missed. We contacted authors of studies to obtain any missing data but received no responses. We examined lists of UK cohort studies from the Medical Research Council and contacted study investigators (individually or via data request services such as Dementias Platform UK) to obtain access to unpublished data.

### Study selection

Results from the first search were imported into Endnote for removal of duplicates and then titles and abstracts were independently screened for inclusion by NN and FS. Repeat searches were imported into Covidence and screened independently by any two of NN, FS, LZ, TS and AP. The same two authors then independently reviewed full text articles for inclusion. Discrepancies between the two authors were resolved by discussion, with no need to consult a third author on disagreements. Some papers were excluded if they were based on analysis of the same data source as another paper (see Supplementary File, S3). Studies that used the same data source were reviewed by FS and TS, who prioritised studies that were of longer duration, measured ethnicity rather than country of birth, and/or reported more granular ethnicity categories. These criteria were developed a priori by FS and RB.

### Data extraction

Data were extracted for each study independently by any two of FS, NN, LZ and TS into Excel and included: citation details, design, setting, ethnic group(s) included and how ethnicity was measured, participant characteristics, follow up/linkage rates, number of persons in each group, number of events in each group, the measure of effect for mortality comparison (SMR, HR, RR, OR, MR), and what other variables were adjusted for. Risk of bias was assessed using the Risk of Bias in Non-randomised studies—of Exposure (ROBINS-E) tool.^23^ Risk of bias assessments were conducted independently by FS and one of EM, AP, or TS with disagreements resolved by discussion.

### Data synthesis and statistical methods

Data synthesis was carried out by LZ and SKS using R (version 4.4.2). Calculation of missing confidence intervals and combining of some results (e.g. across age groups, causes of death, time periods, sex) was carried out by SKS or LZ, with further details of methods in the Supplementary File (S4). We carried out our pre-specified list of meta-analyses^21^ when there were at least two studies reporting data for a particular ethnic group. Meta-analyses were conducted using random-effects models with restricted maximum likelihood (REML) estimation.

We treated SMR, HR, RR and OR as equivalent measures of a relative risk effect given the low event rates in the population-based samples and the small proportion of ethnic minority persons in the samples. We investigated publication bias by a funnel plot and Begg's test where more than 10 studies were available for quantitative synthesis. We also examined the potential for publication bias via comparison of published and unpublished results, where possible. We assessed the presence of statistical heterogeneity using Cochran's Q and the I^2^ statistic. Where heterogeneity was present, we conducted pre-specified^21^ subgroup and sensitivity analyses aimed at investigating clinical or methodological heterogeneity.

We pre-specified meta-analyses of differences in relative mortality within ethnic groups stratified by country of birth (UK-born vs born outside UK), with each stratum compared to the UK-born White population. We performed a statistical test of subgroup differences by country of birth for each ethnic group.

### Certainty of evidence for individual ethnic groups

We used GRADE (Grading of Recommendations, Assessment, Development, and Evaluations)^24^ to evaluate the level of certainty in the body of evidence on inequalities in all-cause mortality for each ethnic group. GRADE assessments were performed by FS and TS independently with disagreements resolved by discussion.

### Role of the funding source

Charges for use of the UK biobank data were covered by funds from the Australian Research Council Centre of Excellence in Population Ageing Research (CEPAR) (Grant ID: Project number CE170100PRO005). The funder had no role in considering the study design or in the collection, analysis, interpretation of data, writing of the report, or decision to submit the article for publication.

### Ethics approval

No primary data were collected for this study and no individual participant data are included in this manuscript. Data are aggregated from existing publications. For the unpublished studies, data were either provided in aggregate form or were available only in a secure portal with only aggregate results able to be exported. Formal ethics approval was therefore not required.

## Results

**Fig. 1:**
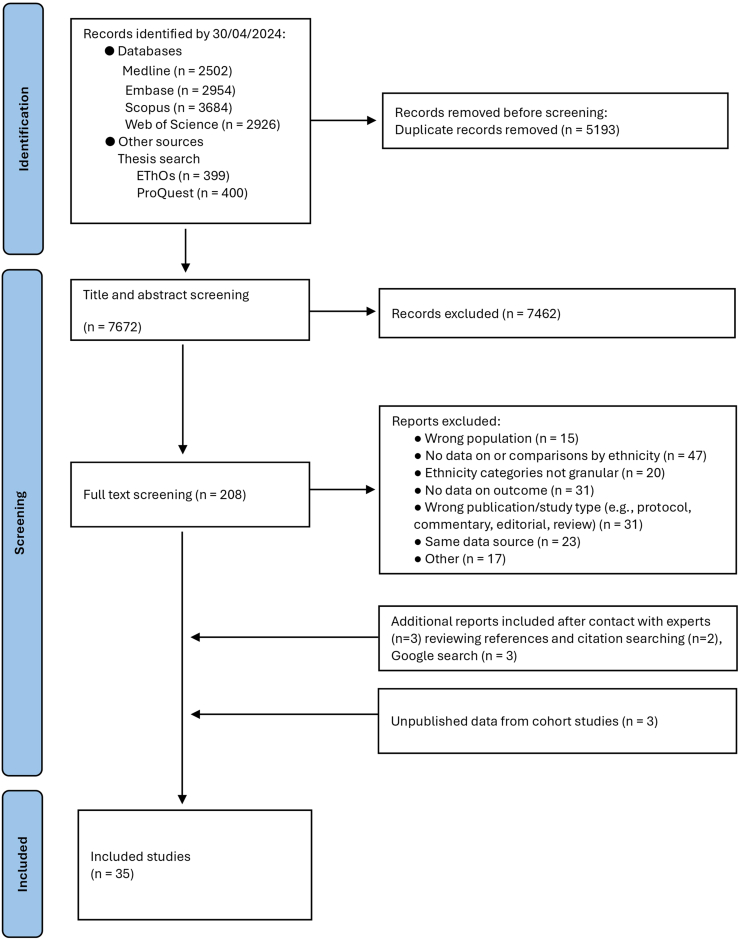
**Flow chart**.

Searches identified 12,865 records of which 5193 were removed as duplicates (Fig. 1). We screened 7672 titles and abstracts, and 208 articles underwent full-text review. We excluded 184 articles leaving 24 (see Supplementary Files (S3)). We located 8 articles from citation searching, Google searches and consultation with experts, giving a total of 32 included articles. We obtained unpublished data from three cohort studies, resulting in 35 included studies.

The characteristics of included studies are shown in the Supplementary Files (S5). Ten studies analysed unlinked Census and death registry data by country of birth.^14^^,^^17^^,^^18^^,^^25^, ^26^, ^27^, ^28^, ^29^, ^30^, ^31^ The remaining 25 studies were cohort studies, including four based on Census data linkage,^12^^,^^15^^,^^32^^,^^33^ six using the Office of National Statistics (ONS) longitudinal study,^34^, ^35^, ^36^, ^37^, ^38^, ^39^ one using the Scottish Longitudinal study,^40^ seven using primary care data linkage,^16^^,^^41^, ^42^, ^43^, ^44^, ^45^, ^46^ and seven studies of recruited participants.^47^, ^48^, ^49^, ^50^, ^51^, ^52^, ^53^ Two of the four Census data linkage studies were published on the ONS website.^15^^,^^33^ We included unpublished data from three cohort studies: EPIC Norfolk,^51^ Million Women's Study^52^ and the UK Biobank.^53^ Other cohort studies we approached did not agree to share data (English Longitudinal Study of Ageing and the Southall and Brent Revisited Study). We obtained unpublished data from the Whitehall Study, but this could not be included as all ethnic groups were categorised together as non-White.

Ethnicity was measured by self-report in most recent studies but was based on country of birth in most older studies. The majority of studies were from England and Wales but there were also seven studies^12^^,^^14^^,^^16^^,^^29^^,^^31^^,^^40^^,^^47^ that were either wholly or partially based in Scotland and two with data from Northern Ireland.^16^^,^^32^ The comparison population was the White English and Welsh in studies in England and Wales and the White Scottish or White Irish populations in studies based in Scotland or Northern Ireland. In studies estimating standardised mortality ratios the whole population was the comparison group.

**Fig. 2:**
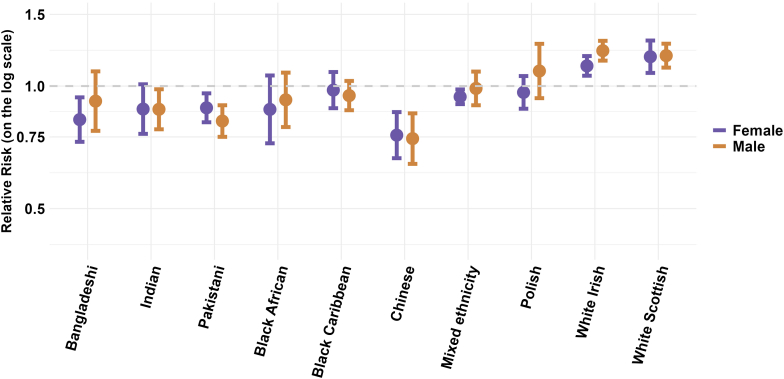
**Meta-analysis summary estimates of age-adjusted all-cause mortality by ethnicity, stratified by sex**. Relative risks and 95% confidence intervals shown in the Figure are also provided in Table 1. The forest plots that produced the presented summary estimates for each ethnic group are provided in the Supplementary File, Supplements 6 and 7.

Fig. 2 and Table 1 present the summary estimates and confidence intervals from the meta-analyses of age adjusted all-cause mortality in each ethnic group, stratified by sex compared to the White reference population. Detailed forest plots from each meta-analysis are provided in the Supplementary Files (S6, S7). The results demonstrate a 22% higher relative mortality in White Irish males (RR = 1.22; 95% Confidence Interval (CI): 1.15–1.29) and a 12% higher relative mortality in White Irish females (1.12; 1.06–1.18), a 19% higher relative mortality in White Scottish males (1.19; 1.11–1.27) and an 18% higher relative mortality in White Scottish females (1.18; 1.08–1.29). There was a 26% lower relative mortality in Chinese males (0.74; 0.64–0.86) and 24% lower relative mortality in Chinese females (0.76; 0.67–0.86). Lower relative mortality of 6–18% was also observed in Bangladeshi females (0.83, 0.73–0.94), Indian males (0.88, 0.78–0.98), Pakistani males (0.82, 0.75–0.90), Pakistani females (0.88, 0.81–0.96), and females of Mixed ethnicity (0.94, 0.90–0.98). Confidence intervals for estimates of relative mortality all crossed the null effect (1.0) in Bangladeshi males, Indian females, Black African males and females, Black Caribbean males and females, Polish males and females, and males of Mixed ethnicity. However, most point estimates were supportive of lower relative mortality.

**Table 1:** Comparison of results of meta-analyses with all studies included compared to meta-analyses restricted to cohort studies with only low or some risk of bias.

Ethnic group	Analysis with all studies included					Analysis restricted to cohort studies with low or some risk of bias only				
	Summary estimate	Confidence interval	Statistical heterogeneity (p-value)	I^2^ (%)	Important inconsistency^a^	Summary estimate	Confidence interval	Statistical heterogeneity (p-value)	I^2^ (%)	Important inconsistency^a^
**Males**										
Bangladeshi	0.92	0.78–1.09	<0.0001	97	Yes	0.89	0.86–0.93	0.0436	20	No
Indian	0.88	0.78–0.98	<0.0001	99	Yes	0.78	0.69–0.88	<0.0001	98	No
Pakistani	0.82	0.75–0.90	<0.0001	96	No	0.76	0.69–0.83	<0.0001	92	No
Black African	0.93	0.79–1.08	<0.0001	98	Yes	0.77	0.64–0.93	<0.0001	97	Yes
Black Caribbean	0.95	0.87–1.03	<0.0001	97	Yes	0.89	0.85–0.93	0.0498	69	No
Chinese	0.74	0.64–0.86	<0.0001	92	No	0.66	0.50–0.87	0.0013	90	No
Mixed ethnicity	0.99	0.90–1.09	<0.0001	89	Yes	0.98	0.88–1.10	<0.0001	94	Yes
Polish	1.09	0.93–1.27	<0.0001	98	Yes	–	–	–	–	–
White Irish	1.22	1.15–1.29	<0.0001	97	No	1.20	1.06–1.34	0.0025	83	No
White Scottish	1.19	1.11–1.27	<0.0001	98	No	–	–	–	–	–
**Females**										
Bangladeshi	0.83	0.73–0.94	<0.0001	86	Yes	0.80	0.67–0.95	<0.0001	92	No
Indian	0.88	0.76–1.01	<0.0001	99	Yes	0.78	0.68–0.88	0.0006	98	No
Pakistani	0.88	0.81–0.96	<0.0001	93	Yes	0.85	0.79–0.91	0.0004	83	No
Black African	0.88	0.72–1.06	<0.0001	99	Yes	0.67	0.56–0.80	<0.0001	96	Yes
Black Caribbean	0.98	0.88–1.08	<0.0001	98	Yes	0.83	0.81–0.85	0.0686	37	No
Chinese	0.76	0.67–0.86	<0.0001	88	No	0.64	0.61–0.69	0.9317	0	No
Mixed ethnicity	0.94	0.90–0.98	0.1770	47	No	0.94	0.90–0.98	0.1033	59	No
Polish	0.96	0.88–1.06	0.0017	80	Yes	–	–	–	–	–
White Irish	1.12	1.06–1.18	<0.001	95	Yes	1.06	0.87–1.29	<0.0001	93	No
White Scottish	1.18	1.08–1.29	<0.001	99	No	–	–	–	–	–

a Inconsistency was considered important if there was statistically significant heterogeneity with a high I^2^ AND the point estimates of included studies were on either side of the null.^49^

**Fig. 3:**
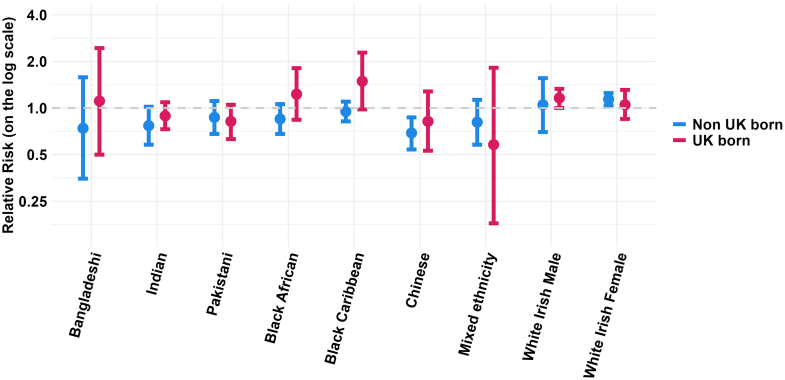
**Meta-analysis summary estimates of age and sex-adjusted all-cause mortality by ethnicity, stratified by country of birth**. Relative risks and 95% confidence intervals shown in the Figure are also provided in Table 2. The forest plots that produced the presented summary estimates for each ethnic group are provided in the Supplementary File, Supplement S8.

Data were only available from five studies for the analysis of country of birth differences within ethnic groups^12^^,^^35^^,^^36^^,^^39^^,^^53^ as most included studies analysed data by either ethnicity or country of birth alone and did not have data on both measures. The summary estimates of relative mortality in each country of birth subgroup (UK-born vs born outside UK) by ethnicity are shown in Fig. 3. Further detailed results are provided in the Supplementary Files (S8). Within Bangladeshi, Black African and Black Caribbean ethnicities, there was a higher relative mortality in the UK-born population, when compared to the UK-born White British, and a lower relative mortality in those born outside of the UK. However, the confidence intervals around these stratified results were wide, with considerable overlap between the UK and non-UK country of birth subgroups. The statistical test for subgroup differences by country of birth was nonsignificant (p < 0.05) in all instances. However, it reached borderline significance (p = 0.07) in the Black African and Black Caribbean ethnic groups (see Supplementary Files, S8). In addition, the UK-born Black Caribbean population had higher relative mortality rate compared to the UK-born White British population (1.49; 0.98–2.28) where the confidence interval only just crossed 1.0.

**Fig. 4:**
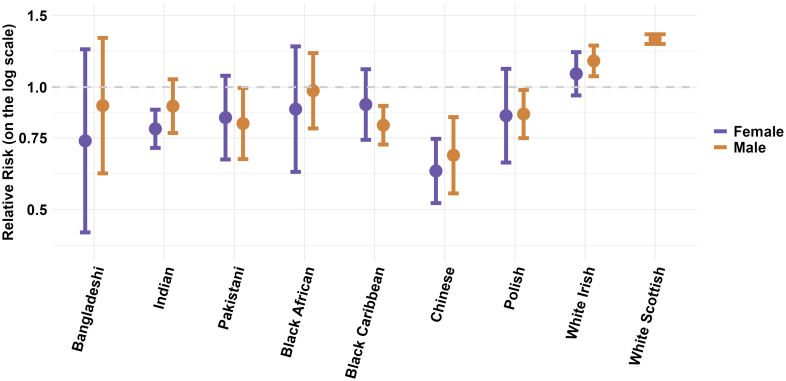
**Meta-analysis summary estimates of age and SEP-adjusted all-cause mortality by ethnicity, stratified by sex**. Relative risks and 95% confidence intervals shown in the Figure are also provided in Table 2. The forest plots that produced the presented summary estimates for each ethnic group are provided in the Supplementary File, Supplement S9.

The summary estimates from the meta-analyses of age and SEP adjusted mortality by ethnicity are shown in Fig. 4 (with more detailed results in Supplementary File, S9). For the majority of ethnic groups, these are similar to the age-adjusted results provided in Fig. 2 (see Supplementary File S10 for table of comparison) but in some ethnic groups with lower relative mortality in age-adjusted analyses, adjustment for SEP resulted in further lowering of relative mortality (Black Caribbean males and females, Chinese males and females, see Supplementary File S10). However, the age-adjusted and age + SEP adjusted analyses do not include the same studies, making direct comparison difficult. There was also considerable methodological heterogeneity in measures of SEP between studies (Supplementary File, S11).

**Fig. 5:**
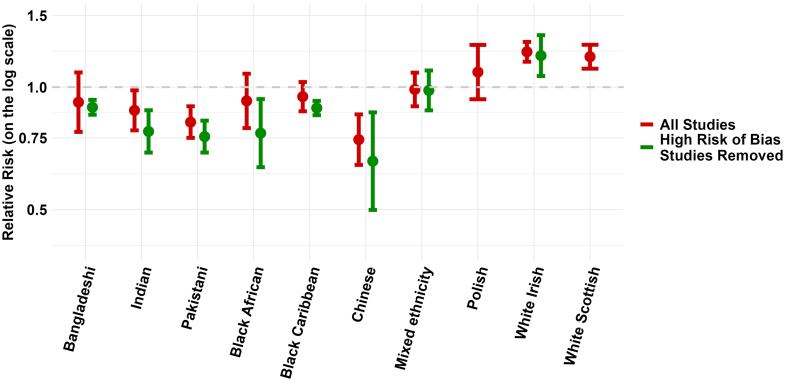
**Meta-analysis summary estimates for age-adjusted all-cause mortality by ethnicity in males in all studies and studies with low or some risk of bias**. Relative risks and 95% confidence intervals shown in the Figure are also provided in Table 1. The forest plots that produced the presented summary estimates for each ethnic group are provided in the Supplementary File, Supplements S6 and S13.

**Fig. 6:**
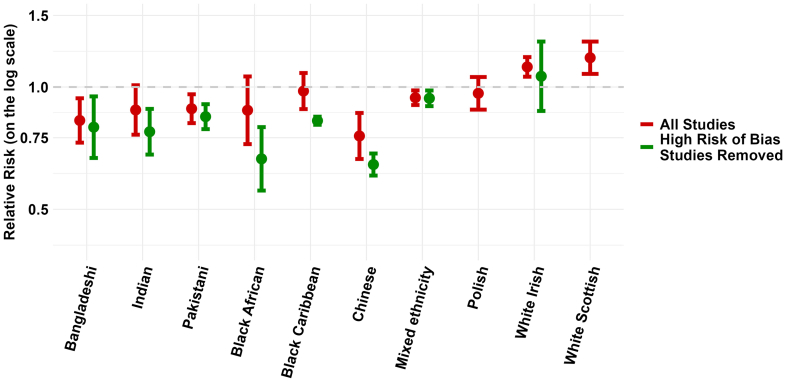
**Meta-analysis summary estimates for age-adjusted all-cause mortality by ethnicity in females in all studies and studies with low or some risk of bias**. Relative risks and 95% confidence intervals shown in the Figure are also provided in Table 1. The forest plots that produced the presented summary estimates for each ethnic group are provided in the Supplementary File, Supplements S7 and S13.

There was high statistical heterogeneity across all ethnic groups in both males and females with most I^2^ results greater than 90% (see Table 1 and Supplementary Files, S6, S7). Restricting the meta-analyses to studies with only a low or some risk of bias (Table 1, Figs. 5 and 6), did not remove statistical heterogeneity or improve the I^2^ result for most ethnic groups in either males or females. However, it did improve the consistency of point estimates (all one side of the null) in almost all groups apart from Black African males and females, and Mixed ethnicity males (Table 1, Supplementary Files, S13).

Excluding studies with a high or very high risk of bias tended to lower the summary estimates for relative all-cause mortality in most ethnic groups. The largest change was observed in Black African males with a relative mortality estimate of 0.77 (0.64–0.93) in the analysis restricted to studies with low or some risk of bias compared to the estimate of 0.93 (0.79–1.08) in the analysis with all studies included. Results in White Irish males and White Scottish males and females had minimal change and results in those of Mixed ethnicity were unchanged. In other ethnic groups, in the analysis restricted to high-quality studies, we observed 34% lower relative mortality in Chinese males (0.66; 0.50–0.87) and 36% lower relative mortality in Chinese females (0.64; 0.61–0.69). Lower relative mortality of 11–22% was also observed in Bangladeshi males (0.89, 0.86–0.92), Bangladeshi females (0.80, 0.67–0.95), Indian males (0.78, 0.69–0.88), Indian females (0.78, 0.68 = 0.88), Pakistani males (0.79, 0.69–0.83), Pakistani females (0.85, 0.79–0.91), Black Caribbean males (0.89, 0.85–0.93), and Black Caribbean females (0.83, 0.81–0.85).

We found statistically significant subgroup differences based on the comparison population used (English/Welsh vs Scottish) for the White Irish, Pakistani, and Bangladeshi ethnic groups in males and the White Irish and Pakistani ethnic groups in females (Supplementary Files, S14). In all these groups, relative mortality estimates were lower when the comparison population was White Scottish or Scotland-born than when compared to the English and Welsh or England/Wales-born populations. However, statistical heterogeneity remained high among the studies with an English/Welsh comparison population. None of the other pre-specified or post hoc (COVID-19) subgroup or sensitivity analyses were able to explain observed statistical heterogeneity or demonstrated statistically significant subgroup differences (see online repository link in Supplementary Files, S18).

There were sufficient studies to formally test for publication bias using a funnel plot and Egger's test for the meta-analysis of White Irish ethnicity in males only (Supplementary File, S15). This did not support the presence of publication bias, nor did any of our sensitivity or subgroup analyses (see online repository link in Supplementary Files, S18).

The risk of bias of included studies ranged from low to very high, with important differences in the risk of bias for individual ethnic groups within studies (Supplementary File, S16). Unlinked Census and death registry studies had a very high risk of bias, largely driven by using country of birth as a proxy for ethnicity and the risk of numerator-denominator mismatch in these unlinked studies. In contrast, most cohort studies received a low risk of bias rating for most domains. However, the UK Biobank and EPIC Norfolk studies received a high risk of bias for selection due to the under-representation of ethnic minority persons in their samples.

Results of the GRADE assessment of the certainty of evidence are shown in the summary of findings table (Table 2). We based GRADE assessments on the meta-analyses restricted to studies with low or some risk of bias given the substantial improvement in consistency of results when studies with high and very high risk of bias were removed.

**Table 2:** GRADE summary of findings table.

GRADE assessment (Table 2 and Supplementary File, S17) demonstrated moderate certainty of evidence for higher mortality observed in White Irish males, White Scottish males and females and for lower mortality observed in Bangladeshi, Indian, Pakistani, Black Caribbean and Chinese males and females. There was moderate certainty of the very small lower relative mortality observed in females of Mixed ethnicity. There was low or very low certainty around estimates of differences in all-cause mortality in Black African males and females, males of Mixed ethnicity, Polish males and females, and White Irish females. The lower certainty of evidence for most of these ethnic groups was a result of inconsistency^54^ (heterogeneity) and/or imprecision (confidence interval crosses no effect). In the Polish, the very low certainty was also contributed to by the high risk of bias from included studies, which were all unlinked Census and death registry studies. GRADE assessment of the role of SEP and country of birth in mortality inequalities was low to very low across all ethnic groups.

## Discussion

Our systematic review and meta-analysis of 35 published and unpublished studies examining ethnic inequalities in mortality in the UK found higher relative mortality in White Irish males and White Scottish males and females and lower mortality in Bangladeshi, Indian, Pakistani, Black Caribbean and Chinese males and females compared to White British populations in the UK. Evidence also suggests that the UK-born Black Caribbean population has higher mortality than the UK-born White British population. There was limited high quality evidence of mortality inequalities in Polish males and females, Black African males and females and males of Mixed ethnicity. There was also insufficient high-quality evidence on the role of either SEP or country of birth in observed mortality inequalities.

SEP is considered an important driver of ethnic health inequalities. However, we were unable to produce high-quality evidence about the potential mediation effects of SEP in our review. Different studies were included in the meta-analyses adjusted and unadjusted for SEP inequalities, meaning that results cannot simply be compared between the adjusted and unadjusted analyses. The evidence provided by these meta-analyses is further limited by the large methodological heterogeneity between studies in terms of which measure of SEP was used. Finally, many non-white ethnic groups demonstrated lower mortality despite their lower SEP. As SEP is typically associated with higher mortality, adjustment for SEP would likely further widen the difference in mortality observed. The exceptions would be the White Scottish and White Irish which had high relative mortality in comparison to White British populations, where narrowing of the gap would be expected.

The intersection of ethnicity and country of birth and how this relates to the presence of health inequalities is rarely considered in studies of ethnicity and health in the UK, with these analyses only conducted in studies by Wallace,^39^ Bhopal,^12^ Scott^36^ and Harding.^35^ We also conducted our own country of birth stratified analyses using unpublished UK biobank data. Given the growing size of the UK-born population and the suggestion of higher mortality amongst the UK-born Black Caribbean population, it is essential that more studies consider differences by country of birth within ethnic groups. Migrants to high-income countries such as the UK are a highly selected population and as a result frequently demonstrate lower mortality than non-migrants despite experiencing socioeconomic disadvantage and harmful exposures such as racism.^11^^,^^55^ Artefactual causes of low mortality in migrants have also been found including salmon bias which refers to missing data on mortality from unwell migrants who return to their country of origin. However, work by Wallace^56^ investigating the salmon bias effect in the UK has found that even when present, salmon bias is insufficient to explain the lower mortality observed in migrants in the UK.

In contrast, the locally born descendants of migrants do not benefit from migration selection effects but may experience the same or greater harmful exposures. However, some ethnic groups may equally enjoy health benefits from cultural taboos around high-risk behaviours such as smoking and alcohol, some of which can continue across generations.^11^^,^^57^ Importantly, these differences between the health of migrants and their descendants may differ by ethnicity, further highlighting the need for greater evidence. For example, past research in France using surnames to identify the children of migrants found that although North African migrants demonstrated a mortality advantage, their French-born children demonstrated a large mortality disadvantage which appeared largely explained by higher unemployment.^58^ In contrast, the French-born children of Italian migrants demonstrated a similar mortality advantage to their migrant parents that was not altered by adjustment for measures of SEP.^58^

This is the first systematic review to the best of our knowledge that has synthesised the large body of evidence examining mortality inequalities by ethnicity in the UK. Our protocol was peer reviewed and published,^21^ strengthening our methodological approach. We carried out an extensive search for both published and unpublished literature and were able to include unpublished data from several cohort studies in our meta-analyses. The increase in size of most mortality inequalities after the removal of studies at high risk of bias further reinforces the robustness of our findings. Despite these key strengths, the following limitations of the review and the evidence-base are also important to consider when interpreting our findings and providing recommendations for future research.

Some of the studies included in the systematic review would represent the same population analysed over different time periods. This would particularly be the case for analyses that included data from Census linked cohort studies that have included almost the entire population of England and Wales at the time of the Census. This could introduce bias due to due failure to meet the independence assumption. However, we consider that the conclusions would remain similar even if such studies were removed given the similarity of findings across time periods and different populations included.

There is an almost complete lack of evidence about mortality inequalities experienced by those of Polish ethnicity in the UK, with evidence only from two unlinked Census and death registry studies. This lack of evidence is important given that the Polish represent the largest non-British White ethnicity group in the 2021 England and Wales Census.^59^ There is similarly very limited evidence about the health of the White Scottish population and what is available is usually limited to consideration of country of birth alone. There is a larger volume of research about the White Irish population, but again this is largely based on country of birth and not ethnicity. The lack of research examining differences within ethnic groups by country of birth in the UK is important and likely data driven. In many studies, both published and unpublished, it was only ethnicity or country of birth data that was available. However, data on both measures is available in the Census and we would encourage reporting of mortality estimates by ethnicity published on the Office of National Statistics website to be stratified by country of birth where possible.

Our findings support the existence of important mortality inequalities by ethnicity in the UK. In contrast to the higher relative mortality observed in the White Irish and White Scottish, most non-white ethnic minority groups demonstrated lower relative mortality. This is despite some non-White ethnic minority groups experiencing higher levels of socioeconomic deprivation,^8^ higher mortality from particular causes of death such as cardiovascular disease and diabetes,^15^ higher mortality from COVID-19 in the first two years of the pandemic,^60^ higher maternal and infant mortality,^61^^,^^62^ poorer self-rated health^63^ and poorer perceived quality of health care.^64^ These paradoxical findings of low mortality in the presence of low SEP and poorer health have also been found in migrants in other countries.^65^^,^^66^ This highlights the complex nature of health inequalities between different ethnic groups in the UK and elsewhere with the interplay of multiple contributing factors operating in different directions including migration selection effects, culturally based differences in lifestyle behaviours, socioeconomic deprivation and racism.

In summary, in this comprehensive, and to our knowledge first, systematic review we found that inequalities in mortality by ethnic group were consistent and substantial. The contribution of SEP to observed inequalities is unclear but unlikely to explain the mortality advantage of non-white groups. Differences in inequalities within ethnic groups by country of birth have important policy implications but have received limited research attention in the UK to date.

## Contributors

The initial study idea was conceived by RB and FS. FS, NN, EM, SKS and RB drafted the original study protocol. FS, NN, AP, TS and LZ contributed to searches, screening of abstracts and full text, and data extraction. FS, EM, AP, and TS contributed to risk of bias assessment. LZ and SKS carried out the quantitative analyses. FS wrote the first draft of the manuscript, with critical input from all authors. All authors had full access to all the data in the study and had final responsibility for the decision to submit for publication. FS, TS, LZ and SKS directly accessed and verified the underlying data reported in the manuscript.

## Data sharing statement

The search strategy, characteristics of included studies and a list of studies excluded on full text review are provided in the Supplementary Files (S2, S3 and S5). The study protocol has been published open access.^21^ Extracted data used in analyses is available in the published articles or from the corresponding author upon reasonable request. Unpublished data included in the meta-analysis is not available as it was only accessible via a secure portal for a limited time or was not made available to us and we were provided with aggregate estimates by cohort study coordinators. Researchers registered with UK Biobank can apply for access to the database by completing an application. This must include a summary of the research plan, data-fields required, any new data or variables that will be generated, and payment to cover the incremental costs of servicing an application (https://www.ukbiobank.ac.uk/enable-your-research/apply-for-access).

## Declaration of interests

We decare no competing interests.
