# Supplementary material for: Inequalities in all-cause mortality by ethnicity in the United Kingdom: a systematic review and meta-analysis
Source: Lancet Reg Health Eur. 2025 Oct 21;59:101498. doi: 10.1016/j.lanepe.2025.101498 (PMC12744721; doi:10.1016/j.lanepe.2025.101498)
Supplement: Supplementary Files [file mmc1.docx]

**Supplemental material for the manuscript**

**Inequalities in all-cause mortality by ethnicity in the United Kingdom: a systematic review and meta-analysis**

Stanaway F, Khalatbari-Soltani S, Zhu L, Sreeharan T, Noguchi N, Pathak A, Mathieu E & Bhopal R

Contents

[Supplement 1: PRISMA Checklists 2](#_Toc198455000)

[Supplement 2: Search strategy 7](#_Toc198455001)

[Supplement 3: Excluded articles at full text screening with main reason for exclusion 9](#_Toc198455002)

[Supplement 4: Methods to combine study results 28](#_Toc198455003)

[Supplement 5: Table of characteristics for included studies 30](#_Toc198455004)

[Supplement 6: Age-adjusted all-cause mortality by ethnicity in males 40](#_Toc198455005)

[Supplement 7: Age-adjusted all-cause mortality by ethnicity in females 43](#_Toc198455006)

[Supplement 8: Age-adjusted all-cause mortality by ethnicity, stratified by country of birth 46](#_Toc198455007)

[Supplement 9: Age and SEP adjusted all-cause mortality by ethnicity 51](#_Toc198455010)

[Supplement 10: Table comparing age adjusted and age + SEP adjusted results 57](#_Toc198455013)

[Supplement 11: Table of SEP measures used in included studies 58](#_Toc198455014)

[Supplement 12: Sex adjusted analyses 60](#_Toc198455015)

[Supplement 13: Age-adjusted all-cause mortality by ethnicity, restricted to studies with low or some risk of bias 66](#_Toc198455018)

[Supplement 14: Sub-group analyses by comparison population (Scottish vs English and Welsh) 72](#_Toc198455021)

[Supplement 15: Funnel plot for assessment of publication bias in age adjusted all-cause mortality in Irish males 84](#_Toc198455032)

[Supplement 16: Risk of bias assessment by analysis and study 85](#_Toc198455033)

[Supplement 17: Detailed GRADE assessment for each ethnic group and analysis 89](#_Toc198455034)

[Supplement 18: Online repository 109](#_Toc198455035)

[Supplement 19: Analysis code 109](#_Toc198455035)

# Supplement 1: PRISMA Checklists


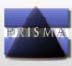
**PRISMA 2020 for Abstracts Checklist**

| **Section and Topic** | **Item #** | **Checklist item** | **Reported (Yes/No)** |
| --- | --- | --- | --- |
| **TITLE** | | |  |
| Title | 1 | Identify the report as a systematic review. | Yes |
| **BACKGROUND** | | |  |
| Objectives | 2 | Provide an explicit statement of the main objective(s) or question(s) the review addresses. | Yes |
| **METHODS** | | |  |
| Eligibility criteria | 3 | Specify the inclusion and exclusion criteria for the review. | Yes |
| Information sources | 4 | Specify the information sources (e.g. databases, registers) used to identify studies and the date when each was last searched. | Yes |
| Risk of bias | 5 | Specify the methods used to assess risk of bias in the included studies. | Yes |
| Synthesis of results | 6 | Specify the methods used to present and synthesise results. | Yes |
| **RESULTS** | | |  |
| Included studies | 7 | Give the total number of included studies and participants and summarise relevant characteristics of studies. | Yes |
| Synthesis of results | 8 | Present results for main outcomes, preferably indicating the number of included studies and participants for each. If meta-analysis was done, report the summary estimate and confidence/credible interval. If comparing groups, indicate the direction of the effect (i.e. which group is favoured). | Yes (only main results reported as too many) |
| **DISCUSSION** | | |  |
| Limitations of evidence | 9 | Provide a brief summary of the limitations of the evidence included in the review (e.g. study risk of bias, inconsistency and imprecision). | Yes, some mention of inconsistency and methodological concerns. Rest in main text. |
| Interpretation | 10 | Provide a general interpretation of the results and important implications. | Yes |
| **OTHER** | | |  |
| Funding | 11 | Specify the primary source of funding for the review. | Yes, as per BMJ requirements |
| Registration | 12 | Provide the register name and registration number. | Yes |

*From:*  Page MJ, McKenzie JE, Bossuyt PM, Boutron I, Hoffmann TC, Mulrow CD, et al. The PRISMA 2020 statement: an updated guideline for reporting systematic reviews. BMJ 2021;372:n71. doi: 10.1136/bmj.n71. This work is licensed under CC BY 4.0. To view a copy of this license, visit <https://creativecommons.org/licenses/by/4.0/>


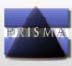
**PRISMA 2020 Checklist**

| **Section and Topic** | **Item #** | **Checklist item** | **Location where item is reported** |
| --- | --- | --- | --- |
| **TITLE** | | |  |
| Title | 1 | Identify the report as a systematic review. | Title page |
| **ABSTRACT** | | |  |
| Abstract | 2 | See the PRISMA 2020 for Abstracts checklist. | Abstract, checklist in Supplementary file, S1 |
| **INTRODUCTION** | | |  |
| Rationale | 3 | Describe the rationale for the review in the context of existing knowledge. | P3 |
| Objectives | 4 | Provide an explicit statement of the objective(s) or question(s) the review addresses. | P4 |
| **METHODS** | | |  |
| Eligibility criteria | 5 | Specify the inclusion and exclusion criteria for the review and how studies were grouped for the syntheses. | P4,5 |
| Information sources | 6 | Specify all databases, registers, websites, organisations, reference lists and other sources searched or consulted to identify studies. Specify the date when each source was last searched or consulted. | P5 |
| Search strategy | 7 | Present the full search strategies for all databases, registers and websites, including any filters and limits used. | Supplementary file, S2 |
| Selection process | 8 | Specify the methods used to decide whether a study met the inclusion criteria of the review, including how many reviewers screened each record and each report retrieved, whether they worked independently, and if applicable, details of automation tools used in the process. | P5,6 |
| Data collection process | 9 | Specify the methods used to collect data from reports, including how many reviewers collected data from each report, whether they worked independently, any processes for obtaining or confirming data from study investigators, and if applicable, details of automation tools used in the process. | P6 |
| Data items | 10a | List and define all outcomes for which data were sought. Specify whether all results that were compatible with each outcome domain in each study were sought (e.g. for all measures, time points, analyses), and if not, the methods used to decide which results to collect. | P6 |
|  | 10b | List and define all other variables for which data were sought (e.g. participant and intervention characteristics, funding sources). Describe any assumptions made about any missing or unclear information. | P6 |
| Study risk of bias assessment | 11 | Specify the methods used to assess risk of bias in the included studies, including details of the tool(s) used, how many reviewers assessed each study and whether they worked independently, and if applicable, details of automation tools used in the process. | P6 |
| Effect measures | 12 | Specify for each outcome the effect measure(s) (e.g. risk ratio, mean difference) used in the synthesis or presentation of results. | P6,7 |
| Synthesis methods | 13a | Describe the processes used to decide which studies were eligible for each synthesis (e.g. tabulating the study intervention characteristics and comparing against the planned groups for each synthesis (item #5)). | P6 |
|  | 13b | Describe any methods required to prepare the data for presentation or synthesis, such as handling of missing summary statistics, or data conversions. | P6, Supplementary file, S4 |
|  | 13c | Describe any methods used to tabulate or visually display results of individual studies and syntheses. | P7 |
|  | 13d | Describe any methods used to synthesize results and provide a rationale for the choice(s). If meta-analysis was performed, describe the model(s), method(s) to identify the presence and extent of statistical heterogeneity, and software package(s) used. | P7 |
|  | 13e | Describe any methods used to explore possible causes of heterogeneity among study results (e.g. subgroup analysis, meta-regression). | P7 |
|  | 13f | Describe any sensitivity analyses conducted to assess robustness of the synthesized results. | P7 |
| Reporting bias assessment | 14 | Describe any methods used to assess risk of bias due to missing results in a synthesis (arising from reporting biases). | P7 |
| Certainty assessment | 15 | Describe any methods used to assess certainty (or confidence) in the body of evidence for an outcome. | P7 |
| **RESULTS** | | |  |
| Study selection | 16a | Describe the results of the search and selection process, from the number of records identified in the search to the number of studies included in the review, ideally using a flow diagram. | P9, Figure 1 |
|  | 16b | Cite studies that might appear to meet the inclusion criteria, but which were excluded, and explain why they were excluded. | Supplementary file, S3 |
| Study characteristics | 17 | Cite each included study and present its characteristics. | P8,9 Supplementary file, S5 |
| Risk of bias in studies | 18 | Present assessments of risk of bias for each included study. | P13, Table 2, Supplementary file, S16 |
| Results of individual studies | 19 | For all outcomes, present, for each study: (a) summary statistics for each group (where appropriate) and (b) an effect estimate and its precision (e.g. confidence/credible interval), ideally using structured tables or plots. | Figures 2-4, Table 1, Supplementary files, S6-10,12 |
| Results of syntheses | 20a | For each synthesis, briefly summarise the characteristics and risk of bias among contributing studies. | Supplementary file, S5, S16 |
|  | 20b | Present results of all statistical syntheses conducted. If meta-analysis was done, present for each the summary estimate and its precision (e.g. confidence/credible interval) and measures of statistical heterogeneity. If comparing groups, describe the direction of the effect. | Figures 2-6, Tables 1, 2, Supplementary files, S6-9, S10-14, 17, online repository |
|  | 20c | Present results of all investigations of possible causes of heterogeneity among study results. | P11, 12, Figures 5, 6, Supplementary files, S13,14, online repository |
|  | 20d | Present results of all sensitivity analyses conducted to assess the robustness of the synthesized results. | Online repository |
| Reporting biases | 21 | Present assessments of risk of bias due to missing results (arising from reporting biases) for each synthesis assessed. | Supplementary file, S16 |
| Certainty of evidence | 22 | Present assessments of certainty (or confidence) in the body of evidence for each outcome assessed. | Table 2, P13, Supplementary file, S17 |
| **DISCUSSION** | | |  |
| Discussion | 23a | Provide a general interpretation of the results in the context of other evidence. | P14,15,17 |
|  | 23b | Discuss any limitations of the evidence included in the review. | P16 |
|  | 23c | Discuss any limitations of the review processes used. | P16 |
|  | 23d | Discuss implications of the results for practice, policy, and future research. | P16-18 |
| **OTHER INFORMATION** | | |  |
| Registration and protocol | 24a | Provide registration information for the review, including register name and registration number, or state that the review was not registered. | Abstract, P4 |
|  | 24b | Indicate where the review protocol can be accessed, or state that a protocol was not prepared. | P4 |
|  | 24c | Describe and explain any amendments to information provided at registration or in the protocol. | P6,12 |
| Support | 25 | Describe sources of financial or non-financial support for the review, and the role of the funders or sponsors in the review. | P19 |
| Competing interests | 26 | Declare any competing interests of review authors. | P20 |
| Availability of data, code and other materials | 27 | Report which of the following are publicly available and where they can be found: template data collection forms; data extracted from included studies; data used for all analyses; analytic code; any other materials used in the review. | P18 |

*From:*  Page MJ, McKenzie JE, Bossuyt PM, Boutron I, Hoffmann TC, Mulrow CD, et al. The PRISMA 2020 statement: an updated guideline for reporting systematic reviews. BMJ 2021;372:n71. doi: 10.1136/bmj.n71. This work is licensed under CC BY 4.0. To view a copy of this license, visit <https://creativecommons.org/licenses/by/4.0/>

# Supplement 2: Search strategy

**Embase**

(exp United Kingdom/) OR (england*.mp.) OR (scotland*.mp.) OR (northern ireland.mp.) OR (wales.mp.) OR (united kingdom.mp.) OR (britain.mp.) OR (exp United Kingdom/ or england*.mp. or scotland*.mp. or northern ireland.mp. or wales.mp. or united kingdom.mp. or britain.mp.) OR (exp ethnic group/) OR (exp migrant/) OR (exp ancestry group/) OR (exp "ethnic or racial aspects"/) OR (polish.mp.) OR (indian.mp.) OR (pakistan*.mp.) OR (bangladesh*.mp.) OR (chinese.mp.) OR (african.mp.) OR (caribbean.mp.) OR (black.mp.) OR (mixed.mp.) OR (asian.mp.) OR (ethnic*.mp.) OR (exp ethnic group/ or exp migrant/ or exp ancestry group/ or exp "ethnic or racial aspects"/ or polish.mp. or indian.mp. or pakistan*.mp. or bangladesh*.mp. or chinese.mp. or african.mp. or caribbean.mp. or black.mp. or mixed.mp. or asian.mp. or ethnic*.mp.) OR (exp mortality/) OR (exp death/) OR (mortal*.mp.) OR (exp mortality/ or exp death/ or mortal*.mp.) OR (exp United Kingdom/ or england*.mp. or scotland*.mp. or northern ireland.mp. or wales.mp. or united kingdom.mp. or britain.mp. and exp ethnic group/ or exp migrant/ or exp ancestry group/ or exp "ethnic or racial aspects"/ or polish.mp. or indian.mp. or pakistan*.mp. or bangladesh*.mp. or chinese.mp. or african.mp. or caribbean.mp. or black.mp. or mixed.mp. or asian.mp. or ethnic*.mp. and exp mortality/ or exp death/ or mortal*.mp.)

**Medline**

(exp United Kingdom/) OR (england*.mp.) OR (scotland*.mp.) OR (northern ireland.mp.) OR (wales.mp.) OR (united kingdom.mp.) OR (britain.mp.) OR (exp United Kingdom/ or england*.mp. or scotland*.mp. or northern ireland.mp. or wales.mp. or united kingdom.mp. or britain.mp.) OR (exp Ethnic Groups/) OR (exp Minority Groups/) OR (exp "Transients and Migrants"/) OR (exp "Emigrants and Immigrants"/) OR (polish.mp.) OR (indian.mp.) OR (pakistan*.mp.) OR (bangladesh*.mp.) OR (chinese.mp.) OR (african.mp.) OR (caribbean.mp.) OR (black.mp.) OR (mixed.mp.) OR (ethnic*.mp.) OR (exp Ethnic Groups/ or exp Minority Groups/ or exp "Transients and Migrants"/ or exp "Emigrants and Immigrants"/ or polish.mp. or indian.mp. or pakistan*.mp. or bangladesh*.mp. or chinese.mp. or african.mp. or caribbean.mp. or black.mp. or mixed.mp. or ethnic*.mp.) OR (exp Mortality/) OR (exp Death/) OR (mortal*.mp.) OR (exp Mortality/ or exp Death/ or mortal*.mp.) OR (exp United Kingdom/ or england*.mp. or scotland*.mp. or northern ireland.mp. or wales.mp. or united kingdom.mp. or britain.mp. and exp Ethnic Groups/ or exp Minority Groups/ or exp "Transients and Migrants"/ or exp "Emigrants and Immigrants"/ or polish.mp. or indian.mp. or pakistan*.mp. or bangladesh*.mp. or chinese.mp. or african.mp. or caribbean.mp. or black.mp. or mixed.mp. or ethnic*.mp. and exp Mortality/ or exp Death/ or mortal*.mp.)

**Scopus**

(TITLE-ABS-KEY("united kingdom" or england or scotland or "northern ireland" or wales or britain)) AND (TITLE-ABS-KEY(ethnic* or minority or migrant* or polish or indian or pakistan* or bangladesh* or chinese or asian or african or caribbean or black)) AND (TITLE-ABS-KEY(mortality or death)) AND (LIMIT-TO(SUBJAREA, "MEDI"))

**Web of Science**

("united kingdom" or england or scotland or "northern ireland" or wales or britain) OR (ethnic* or minority or migrant* or polish or indian or pakistan* or bangladesh or chinese or asian or african or caribbean or black) OR (mortality or death) OR ("united kingdom" or england or scotland or "northern ireland" or wales or Britain and ethnic* or minority or migrant* or polish or indian or pakistan* or bangladesh or chinese or asian or african or caribbean or black and mortality or death)

**Ethos**

“ethnic” and “migrant”

**Proquest Thesis**

noft(ethnic minority) OR noft(migrant) AND noft(mortality)

# Supplement 3: Excluded articles at full text screening with main reason for exclusion

| **Reference** | **Reason for full-text exclusion** |
| --- | --- |
| Abbasi K. Connections with death. Journal of the Royal Society of Medicine 2007;100(5):205. doi: 10.1258/jrsm.100.5.205 | Wrong publication/study type (e.g. protocol, commentary, editorial, review) |
| Abbotts J, Williams R, Davey Smith G. Mortality in men of Irish heritage in West Scotland. Public Health 1998;112(4):229-32. doi: 10.1016/S0033-3506(98)00237-6 | Same data source |
| Abbotts J, Williams R, Ford G, et al. Morbidity and Irish Catholic descent in Britain: An ethnic and religious minority 150 years on. Social Science & Medicine 1997;45(1):3-14. doi: 10.1016/s0277-9536(96)00302-4 | No data on relative all-cause mortality or unable to estimate from available data |
| Abbotts J, Williams R, Ford G. Morbidity and Irish Catholic descent in Britain relating health disadvantage to socio-economic position. Social Science & Medicine 2001;52(7):999-1005. doi: 10.1016/s0277-9536(00)00200-8 | No data on relative all-cause mortality or unable to estimate from available data |
| Adelstein AM, Marmot MG, Bulusu L. Migrant studies in Britain. British Medical Bulletin 1984;40(4):315-19. doi: 10.1093/oxfordjournals.bmb.a071997 | Same data source |
| Adelstein AM, Marmot MG, Dean G, et al. Comparison of mortality of Irish immigrants in England and Wales with that of Irish and British nationals. Irish Medical Journal 1986;79:185–9. | Same data source |
| Adelstein AM. Current vital statistics: methods and interpretation. British Medical Journal 1978;2(6143):983-7. doi: 10.1136/bmj.2.6143.983 | No data on relative all-cause mortality or unable to estimate from available data |
| Adler NE, Stewart J. Preface to The Biology of Disadvantage: Socioeconomic Status and Health. In: Adler NE, Stewart J, eds. Biology of Disadvantage: Socioeconomic Status and Health 2010:1-4. doi: 10.1111/j.1749-6632.2009.05385.x | No data on or comparisons by ethnicity |
| Agarwal S, Watson S. BAME women and health inequality. Anaesthesia 2021;76(S4):10-13. doi: 10.1111/anae.15362 | Wrong publication/study type (e.g. protocol, commentary, editorial, review) |
| Ahmad WI, Kernohan EE, Baker MR. Health of British Asians; a research review. Community Medicine 1989;11(1):49-56. doi: 10.1093/oxfordjournals.pubmed.a042446 | No data on or comparisons by ethnicity |
| Alarilla A, Mondor L, Knight H, et al. Socioeconomic gradient in mortality of working age and older adults with multiple long-term conditions in England and Ontario, Canada. BMC Public Health 2023;23(1):472. doi: 10.1186/s12889-023-15370-y | No data on or comparisons by ethnicity |
| Aldridge RW, Lewer D, Katikireddi SV, et al. Black, Asian and Minority Ethnic groups in England are at increased risk of death from COVID-19: indirect standardisation of NHS mortality data. Wellcome Open Research 2020;5:88. doi: 10.12688/wellcomeopenres.15922.2 | No data on relative all-cause mortality or unable to estimate from available data |
| Ali R, Raleigh V, Majeed A, et al. Life expectancy by ethnic group in England. British Medical Journal (Clinical research ed) 2021;375(8900488, bmj, 101090866):e068537. doi: 10.1136/bmj-2021-068537 | Wrong publication/study type (e.g. protocol, commentary, editorial, review) |
| Allik M, Brown D, Dundas R, et al. Differences in ill health and in socioeconomic inequalities in health by ethnic groups: a cross-sectional study using 2011 Scottish census. Ethnicity & Health 2019. doi: 10.1080/13557858.2019.1643009 | No data on relative all-cause mortality or unable to estimate from available data |
| Anderson JJ, Gray SR, Welsh P, et al. The associations of sugar-sweetened, artificially sweetened and naturally sweet juices with all-cause mortality in 198,285 UK Biobank participants: A prospective cohort study. BMC Medicine 2020;18(1). doi: 10.1186/s12916-020-01554-5 | No data on or comparisons by ethnicity |
| Anderson SG, Hutchings DC, Heald AH, et al. Haemostatic factors, lipoproteins and long-term mortality in a multi-ethnic population of Gujarati, African-Caribbean and European origin. Atherosclerosis 2014;236(1):62-72. doi: 10.1016/j.atherosclerosis.2014.6.4 | No data on relative all-cause mortality or unable to estimate from available data |
| Anya I. Right to health care for vulnerable migrants. Lancet 2007;370(9590):827. doi: 10.1016/S0140-6736%2807%2961410-X | No data on relative all-cause mortality or unable to estimate from available data |
| Arai L, Harding S. A review of the epidemiological literature on the health of UK-born Black Caribbeans. Critical Public Health 2004;14(2):81-116. doi:10.1080/109581590410001725355 | Wrong publication/study type (e.g. protocol, commentary, editorial, review) |
| Asaria M, Griffin S, Cookson R, et al. Estimating the social distribution of health in England. Value in Health 2013;16 (7):A602. doi: 10.1016/j.jval.2013.8.1708 | No data on or comparisons by ethnicity |
| Aspinall PJ. Ethnic groups and our healthier nation: whither the information base? Journal of Public Health Medicine 1999;21(2):125-32. doi: 10.1093/pubmed/21.2.125 | Wrong publication/study type (e.g. protocol, commentary, editorial, review) |
| Aspinall PJ. Measuring the health patterns of the 'mixed/multiple' ethnic group in Britain: data quality problems, reporting issues, and implications for policy. International Journal of Social Research Methodology 2018;21(3):359-71. doi: 10.1080/13645579.2017.1399623 | No data on relative all-cause mortality or unable to estimate from available data |
| Aspinall PJ. The Sri Lankan community of descent in the UK: a neglected population in demographic and health research. South Asian Diaspora 2019;11(1):51-65. doi: 10.1080/19438192.2018.1505065 | Wrong publication/study type (e.g. protocol, commentary, editorial, review) |
| Atkins JL, Masoli JAH, Delgado J, et al. Preexisting Comorbidities Predicting COVID-19 and Mortality in the UK Biobank Community Cohort. Journals of Gerontology Series A-Biological Sciences & Medical Sciences 2020;75(11):2224-30. doi: 10.1093/gerona/glaa183 | Wrong population (e.g. limited to population with a specific disease or condition and/or not based in the UK) |
| Atkins JL, Whincup PH, Morris RW, et al. Dietary patterns and the risk of CVD and all-cause mortality in older British men. British Journal of Nutrition 2016;116(7):1246-55. doi: 10.1017/S0007114516003147 | No data on or comparisons by ethnicity |
| Ayoubkhani D, Nafilyan V, White C, et al. Ethnic-minority groups in England and Wales-factors associated with the size and timing of elevated COVID-19 mortality: a retrospective cohort study linking census and death records. International Journal of Epidemiology 2021;49(6):1951-62. doi: 10.1093/ije/dyaa208 | No data on relative all-cause mortality or unable to estimate from available data |
| Baines DR. Obstacles to equality: Issues for purchaser and provider. Ethnicity and Health 1999;4(3):189-210. doi: 10.1080/13557859998137 | Wrong population (e.g. limited to population with a specific disease or condition and/or not based in the UK) |
| Baker D, Mead N, Campbell S. Inequalities in morbidity and consulting behaviour for socially vulnerable groups. British Journal of General Practice 2002;52(475):124-30. PMID: 11885821 | Ethnicity categories not sufficiently granular |
| Balarajan R, Bulusu L, Adelstein AM, et al. Patterns of mortality among migrants to England and Wales from the Indian sub-continent. British Medical Journal 1984;289(6453):1185-87. doi: 10.1136/bmj.289.6453.1185 | No data on relative all-cause mortality or unable to estimate from available data |
| Balarajan R, McDowall ME. Regional socioeconomic differences in mortality among men in Great Britain today. Public Health 1988;102(1):33-43. doi: 10.1016/S0033-3506(88)80008-8 | No data on or comparisons by ethnicity |
| Balarajan R, Yuen P, Machin D. Inequalities in health: Changes in RHAs in the past decade. British Medical Journal (Clinical research ed) 1987;294(6586):1561-64. doi: 10.1136/bmj.294.6586.1561 | No data on or comparisons by ethnicity |
| Balarajan R. Ethnicity and variations in the nation's health. Health Trends 1995;27(4):114-9. PMID: 10162321 | No data on relative all-cause mortality or unable to estimate from available data |
| Bambra C, Cairns JM, Kasim A, et al. This divided land: An examination of regional inequalities in exposure to brownfield land and the association with morbidity and mortality in England. Health Place 2015;34:257-69. doi:10.1016/j.healthplace.2015.5.10 | Ethnicity categories not sufficiently granular |
| Barnard S, Fryers P, Fitzpatrick J, et al. Inequalities in excess premature mortality in England during the COVID-19 pandemic: a cross-sectional analysis of cumulative excess mortality by area deprivation and ethnicity. BMJ Open 2021;11(12):e052646. doi: 10.1136/bmjopen-2021-052646 | Ethnicity categories not sufficiently granular |
| Bartley M, Carpenter L, Dunnell K, et al. Measuring inequalities in health: An analysis of mortality patterns using two social classifications. Sociology of Health & Illness 1996;18(4):455-74. doi: 10.1111/1467-9566.ep10939068 | No data on or comparisons by ethnicity |
| Basellini U, Alburez-Gutierrez D, Del Fava E, et al. Linking excess mortality to mobility data during the first wave of COVID-19 in England and Wales. SSM - Population Health 2021;14:100799. doi: 10.1016/j.ssmph.2021.100799 | No data on or comparisons by ethnicity |
| Beard E, Brown J, West R, et al. Healthier central England or North-South divide? Analysis of national survey data on smoking and high-risk drinking. BMJ Open 2017;7(3) doi: 10.1136/bmjopen-2016-014210 | No data on relative all-cause mortality or unable to estimate from available data |
| Becares L, Nazroo J, Stafford M. The buffering effects of ethnic density on experienced racism and health. Health Place 2009;15(3):670-8. doi: 10.1016/j.healthplace.2008.10.8 | No data on relative all-cause mortality or unable to estimate from available data |
| Beevers DG, Lip GYH. JNC-VI: A view from the United Kingdom. Journal of Human Hypertension 1998;12(9):649-51. doi: 10.1038/sj.jhh.1000659 | No data on or comparisons by ethnicity |
| Bentham G. Migration and morbidity - implications for geographical studies of disease. Social Science & Medicine 1988;26(1):49-54. doi: 10.1016/0277-9536(88)90044-5 | No data on or comparisons by ethnicity |
| Bhala N, Zaman MJS. Preventing premature mortality in chronic diseases for South Asians in the UK and beyond. Journal of the Royal Society of Medicine 2009;102(11):459-63. doi: 10.1258/jrsm.2009.90112 | No data on relative all-cause mortality or unable to estimate from available data |
| Blaxter M. Evidence on inequality in health from a national survey. The Lancet 1987;330(8549):30-33. doi: 10.1016/S0140-6736(87)93062-5 | No data on or comparisons by ethnicity |
| Bonnechère B, Liu J, Thompson A, et al. Does ethnicity influence dementia, stroke and mortality risk? Evidence from the UK Biobank. Frontiers in Public Health 2023; 11:1111321. doi: 10.3389/fpubh.2023.1111321 | Same data source |
| Brawley OW, Jani AB. Race and Disparities in Health. Current Problems in Cancer 2007;31(3):114-22. doi: 10.1016/j.currproblcancer.2007.2.1 | Wrong population (e.g. limited to population with a specific disease or condition and/or not based in the UK) |
| Bray I, Gibson A, White J. Coronavirus disease 2019 mortality: a multivariate ecological analysis in relation to ethnicity, population density, obesity, deprivation and pollution. Public Health 2020;185:261-63. doi: 10.1016/j.puhe.2020.06.056 | No data on relative all-cause mortality or unable to estimate from available data |
| Brimblecombe N, Dorling D, Shaw M. Migration and geographical inequalities in health in Britain. Social Science & Medicine 2000;50(6):861-78. doi: 10.1016/S0277-9536(99)00371-8 | No data on or comparisons by ethnicity |
| Brimblecombe N, Dorling D, Shaw M. Mortality and migration in Britain, first results from the British Household Panel Survey. Social Science & Medicine 1999;49(7):981-8. doi: 10.1016/S0277-9536(99)00195-1 | No data on or comparisons by ethnicity |
| Brown D, Leyland AH. Scottish mortality rates 2000-2002 by deprivation and small area population mobility. Social Science & Medicine 2010;71(11):1951-7. doi: 10.1016/j.socscimed.2010.09.015 | No data on or comparisons by ethnicity |
| Burns R, Pathak N, Campos-Matos I, et al. Million migrants study of healthcare and mortality outcomes in non-EU migrants and refugees to England: Analysis protocol for a linked population-based cohort study of 1.5 million migrants [version 1; referees: 2 approved, 2 approved with reservations]. Wellcome Open Research 2019;4 doi: 10.12688/wellcomeopenres.15007.1 | Wrong publication/study type (e.g. protocol, commentary, editorial, review) |
| Carballo M, Divino JJ, Zeric D. Migration and health in the European Union. Tropical Medicine & International Health 1998;3(12):936-44. doi: 10.1046/j.1365-3156.1998.337.x | Wrong publication/study type (e.g. protocol, commentary, editorial, review) |
| Carr-Hill RA. Time trends in inequalities in health. Journal of Biosocial Science 1988;20(3):265-74. doi: 10.1017/S002193200000660X | No data on or comparisons by ethnicity |
| Carstairs V. Avoidable deaths in countries of the European community and in Scotland. Health Bulletin 1993;51(3):151-57 | No data on or comparisons by ethnicity |
| Carter PR, Reynolds J, Carter A, et al. Impact of ethnicity on mortality amongst tobacco abusers in the United Kingdom. Heart 2016;102 (Supplement 6):A56-A57. doi: 10.1136/heartjnl-2016-309890.78 | Wrong population (e.g. limited to population with a specific disease or condition and/or not based in the UK) |
| Celis-Morales CA, Lyall DM, Steell L, et al. Associations of discretionary screen time with mortality, cardiovascular disease and cancer are attenuated by strength, fitness and physical activity: Findings from the UK Biobank study. BMC Medicine 2018;16 (1) (no pagination)(77) doi: 10.1186/s12916-018-1063-1 | Other (e.g. ecological not individual data) |
| Champion T. Population review: (3). Migration to from and within the United Kingdom. Population Trends 1996(83):5-16. PMID: 8729791 | No data on relative all-cause mortality or unable to estimate from available data |
| Chan M. Ethnic health matters. Annals of Tropical Paediatrics 1998;18 Suppl:S61-5. doi: 10.1080/02724936.1998.11747982 | Wrong publication/study type (e.g. protocol, commentary, editorial, review) |
| Chandola T. Ethnic and class differences in health in relation to British South Asians: Using the new National Statistics Socio-Economic Classification. Social Science and Medicine 2001;52(8):1285-96. doi: 10.1016/S0277-9536%2800%2900231-8 | No data on relative all-cause mortality or unable to estimate from available data |
| Chaturvedi N. Ethnicity as an epidemiological determinant - crudely racist or crucially important? International Journal of Epidemiology 2001;30(5):925-27. doi: 10.1093/ije/30.5.925 | No data on relative all-cause mortality or unable to estimate from available data |
| Chudasama YV, Khunti K, Gillies CL, et al. Healthy lifestyle and life expectancy in people with multimorbidity in the UK Biobank: A longitudinal cohort study. PLoS Medicine / Public Library of Science 2020;17(9):e1003332. doi: 10.1371/journal.pmed.1003332 | No data on or comparisons by ethnicity |
| Coles B, Zaccardi F, Ling S, et al. Cardiovascular events and mortality in people with and without type 2 diabetes: An observational study in a contemporary multi-ethnic population. Journal of Diabetes Investigation 2021;12(7):1175-82. doi: 10.1111/jdi.13464. | No data on relative all-cause mortality or unable to estimate from available data |
| Congdon P, Shouls S, Curtis S. A multi-level perspective on small-area health and mortality: a case study of England and Wales. International Journal of Population Geography 1997;3(3):243-63. doi: 10.1002/(SICI)1099-1220(199709)3:3<243::AID-IJPG70>3.0.CO;2-V | Ethnicity categories not sufficiently granular |
| Connolly S, O'Reilly D, Rosato M. Increasing inequalities in health: Is it an artefact caused by the selective movement of people? Social Science & Medicine 2007;64(10):2008-15. doi: 10.1016/j.socscimed.2007.2.21 | No data on relative all-cause mortality or unable to estimate from available data |
| Cruickshank JK. Mortality in second generation Irish people living in England and Wales [7]. British Medical Journal 1996;313(7059):753. doi: 10.1136/bmj.313.7059.753a. | Wrong publication/study type (e.g. protocol, commentary, editorial, review) |
| Davies B, Parkes BL, Bennett J, et al. Community factors and excess mortality in first wave of the COVID-19 pandemic in England. Nature Communications 2021;12(1):3755. doi: 10.1038/s41467-021-23935-x | Other (e.g. ecological not individual data) |
| Davies JM, Chilvers C. The study of mortality variations in small administrative areas of England and Wales, with special reference to cancer. Journal of Epidemiology and Community Health 1980;34(2):87-92. doi: 10.1136/jech.34.2.87 | No data on or comparisons by ethnicity |
| de Lusignan S, Joy M, Oke J, et al. Disparities in the excess risk of mortality in the first wave of COVID-19: Cross sectional study of the English sentinel network. Journal of Infection 2020;81(5):785-92. doi: 10.1016/j.jinf.2020.08.037 [published Online First: 20200825] | Other (e.g. ecological not individual data) |
| Delaney L, Fernihough A, Smith JP. Exporting Poor Health: The Irish in England. Demography 2013;50(6):2013-35. doi: 10.1007/s13524-013-0235-z | No data on relative all-cause mortality or unable to estimate from available data |
| Dhalwani NN, Zaccardi F, Davies MJ, et al. Body mass index and mortality in people with and without diabetes: A UK Biobank study. Nutrition, Metabolism and Cardiovascular Diseases 2018;28(12):1208-16. doi: 10.1016/j.numecd.2018.7.7 | Ethnicity categories not sufficiently granular |
| Doolin N. Ethnic minorities: the luck of the Irish? Nursing Standard (Royal College of Nursing (Great Britain):1987) 1994;8(46):40-41 doi: 10.7748/ns.8.46.40.s57 | Wrong publication/study type (e.g. protocol, commentary, editorial, review) |
| Doyle YG, Conway M. Transferable deaths during 1990 for residents of Camberwell Health Authority. Public Health 1992;106(6):449-56. doi: 10.1016/s0033-3506(05)80459-7 | Other (e.g. ecological not individual data) |
| Dr Foster's case notes. British Medical Journal 2004;328(7436):369 | No data on or comparisons by ethnicity |
| Drozd M, Pujades-Rodriguez M, Lillie PJ, et al. Non-communicable disease, sociodemographic factors, and risk of death from infection: a UK Biobank observational cohort study. The Lancet Infectious Diseases 2021;21(8):1184-91. doi: 10.1016/S1473-3099(20)30978-6 [published Online First: 20210301] | Same data source |
| Dunnell K. The changing demographic picture of the UK: national statistician's annual article on the population. Population Trends 2007(130):9-21. PMID: 18240843 | Wrong publication/study type (e.g. protocol, commentary, editorial, review) |
| Elliott J, Bodinier B, Whitaker M et al. COVID-19 mortality in the UK Biobank cohort: revisiting and evaluating risk factors. European Journal of Epidemiology 2021, 36(3), 299–309. doi:10.1007/s10654-021-00722-y | Same data source |
| Elovainio M, Hakulinen C, Pulkki-Raback L, et al. Contribution of risk factors to excess mortality in isolated and lonely individuals: an analysis of data from the UK Biobank cohort study. The Lancet Public Health 2017;2(6):e260-e66. doi: 10.1016/S2468-2667%2817%2930075-0 | Ethnicity categories not sufficiently granular |
| Eriksen A, Tillin T, Connor LO, et al. The impact of health behaviours on incident cardiovascular disease in Europeans and South Asians - A prospective analysis in the UK SABRE study. PLoS ONE 2015;10 (3) (no pagination)(e0117364) doi: 10.1371/journal.pone.117364 | No data on relative all-cause mortality or unable to estimate from available data |
| Evangelopoulos D, Analitis A, Giannakopoulos C, et al. Does climatic zone of birth modify the temperature-mortality association of London inhabitants during the warm season? A time-series analysis for 2004-2013. Environmental Research 2021;193:110357 | No data on or comparisons by ethnicity |
| Finer S, Martin HC, Khan A, et al. Cohort Profile: East London Genes & Health (ELGH), a community-based population genomics and health study in British Bangladeshi and British Pakistani people. International Journal of Epidemiology 2020;49(1):20-21I. doi: 10.1093/ije/dyz174 | Wrong publication/study type (e.g. protocol, commentary, editorial, review) |
| Fox AJ. Socio–economic Differences in Mortality and Morbidity. Scandinavian Journal of Public Health 1990;18(1):1-8. doi: 10.1177/140349489001800101 | Wrong publication/study type (e.g. protocol, commentary, editorial, review) |
| Gaia S, Baboukardos D. Ethnic minorities, income inequalities and the COVID-19 pandemic: evidence from English local councils. Regional Studies 2023;57(10):2006-20. doi: 10.1080/00343404.2022.2157384 | No data on or comparisons by ethnicity |
| Gorsky M, Guntupalli A, Harris B, et al. The 'cultural inflation of morbidity' during the English mortality decline: A new look. Social Science and Medicine 2011;73(12):1775-83. doi: 10.1016/j.socscimed.2011.9.28 | No data on or comparisons by ethnicity |
| Grainger CR. A retrospective estimate of some vital statistics for British Indian-Ocean territory 1965-1973. Journal of the Royal Society of Health 1985;105(5):178-80. doi: 10.1177/146642408510500508 | Wrong population (e.g. limited to population with a specific disease or condition and/or not based in the UK) |
| Griffiths R, White M. Ethnic differences in postneonatal mortality. British Medical Journal 1989;298(6680):1099-100. doi: 10.1136/bmj.298.6680.1099-c | Wrong population (e.g. limited to population with a specific disease or condition and/or not based in the UK) |
| Gruer L, Cézard G, Clark E, et al. Life expectancy of different ethnic groups using death records linked to population census data for 4.62 million people in Scotland. Journal of Epidemiology and Community Health 2016;70(12):1251-54. doi: 10.1136/jech-2016-207426 | Same data source |
| Gruer L, Hart CL, Watt GC. After 50 years and 200 papers, what can the Midspan cohort studies tell us about our mortality? Public Health 2017;142:186-95. doi: 10.1016/j.puhe.2015.6.17 | No data on or comparisons by ethnicity |
| Guillot M, Khlat M, Elo I, et al. Understanding age variations in the migrant mortality advantage: An international comparative perspective. PLoS ONE 2018;13(6):e0199669. doi: 10.1371/journal.pone.199669 | Ethnicity categories not sufficiently granular |
| Harding S, Balarajan R. Longitudinal study of socio-economic differences in mortality among South Asian and West Indian migrants. Ethnicity & Health 2001;6(2):121-8. doi: 10.1080/13557850120068441 | Other (e.g. ecological not individual data) |
| Harding S, Balarajan R. Longitudinal study of the relationship between mortality of migrants from the Indian subcontinent and length of residence in England and Wales. American Journal of Epidemiology 2001;153(11):S62-S62. | Other (e.g. ecological not individual data) |
| Harding S. Mortality of migrants from the Caribbean to England and Wales: effect of duration of residence. International Journal of Epidemiology 2004;33(2):382-6. doi: 10.1093/ije/dyh059 | Other (e.g. ecological not individual data) |
| Harding S. Mortality of migrants from the Indian sub-continent and duration of residence in England and Wales. European Journal of Public Health 2002;12(4):59-59 | Same data source |
| Harding S. Mortality of migrants from the Indian subcontinent to England and Wales: effect of duration of residence. Epidemiology 2003;14(3):287-92. doi:10.1097/01.EDE.0000050369.88416.82 | Same data source |
| Haskey J. Mortality among second generation Irish in England and Wales. British Medical Journal 1996;312(7043):1373-74. doi: 10.1136/bmj.312.7043.1373 | Wrong publication/study type (e.g. protocol, commentary, editorial, review) |
| Hiam L, Zhang CX, Burns R, et al. What can the UK learn from the impact of migrant populations on national life expectancy? Journal of Public Health (Oxford, England) 2022;44(4):e499-e505. doi: 10.1093/pubmed/fdac013 | No data on relative all-cause mortality or unable to estimate from available data |
| Ho FK, Celis Morales C, Anderson J, et al. Association of macronutrient intake with all-cause mortality and cardiovascular disease: Findings from the UK Biobank study. Obesity Facts 2019;12 (Supplement 1):41-42. doi: 10.1159/000489691 | No data on or comparisons by ethnicity |
| Janssen F, Kibele E, Reus Pons M, et al. [Healthy life expectancy of older migrants and non-migrants in three European countries over time]. Journal for Gerontology and Geriatrics 2018;49(6):232-43. doi:10.1007/s12439-018-0267-3 | Ethnicity categories not sufficiently granular |
| Jarman B, Aylin P. Death rates in England and Wales and the United States: variation with age, sex, and race. British Medical Journal (Clinical research ed) 2004;329(7479):1367. doi: 10.1136/bmj.329.7479.1367 | Wrong population (e.g. limited to population with a specific disease or condition and/or not based in the UK) |
| Jones RP. Excess Winter Mortality (EWM) as a Dynamic Forensic Tool: Where, When, Which Conditions, Gender, Ethnicity and Age. International Journal of Environmental Research & Public Health [Electronic Resource] 2021;18(4):23. doi: 10.3390/ijerph18042161 | No data on or comparisons by ethnicity |
| Joy M, Hobbs FR, Bernal JL, et al. Excess mortality in the first COVID pandemic peak: cross-sectional analyses of the impact of age, sex, ethnicity, household size, and long-term conditions in people of known SARS-CoV-2 status in England. British Journal of General Practice 2020;70(701):e890-e98. doi: 10.3399/bjgp20X713393 [published Online First: 20201126] | Same data source |
| Katikireddi SV, Cezard G, Bhopal RS, et al. Assessment of health care, hospital admissions, and mortality by ethnicity: population-based cohort study of health-system performance in Scotland. Lancet Public Health 2018;3(5):e226-e36. doi: 10.1016/S2468-2667(18)30068-9 | Same data source |
| Keenan K, Kulu H, Cox F. Editorial introduction: Social and spatial inequalities in health and mortality: The analysis of longitudinal register data from selected European countries. Population Space and Place 2022. doi: 10.1002/psp.2411 | Wrong publication/study type (e.g. protocol, commentary, editorial, review) |
| Khattar RS, Swales JD, Senior R, et al. Racial variation in cardiovascular morbidity and mortality in essential hypertension. Heart 2000;83(3):267-71. doi: 10.1136/heart.83.3.267 | Wrong population (e.g. limited to population with a specific disease or condition and/or not based in the UK) |
| Kinge JM, Morris S. Variation in the relationship between BMI and survival by socioeconomic status in Great Britain. Economics and Human Biology 2014;12(1):67-82. doi: 10.1016/j.ehb.2013.5.6 | No data on or comparisons by ethnicity |
| Koffman J, Higginson IJ, Aspinall PJ. Minority ethnic groups and our healthier nation (multiple letters). Journal of Public Health Medicine 2000;22(2):245-46. doi: 10.1093/pubmed/22.2.245 | Other (e.g. ecological not individual data) |
| Kontopantelis E, Mamas MA, van Marwijk H, et al. Increasing socioeconomic gap between the young and old: temporal trends in health and overall deprivation in England by age, sex, urbanity and ethnicity, 2004-2015. Journal of Epidemiology and Community Health 2018;72(7):636-44. doi: 10.1136/jech-2017-209895 | No data on relative all-cause mortality or unable to estimate from available data |
| Kumari M, Shipley M, Stafford M, et al. Association of diurnal patterns in salivary cortisol with all-cause and cardiovascular mortality: findings from the Whitehall II study. The Journal of Clinical Endocrinology and Metabolism 2011;96(5):1478-85. doi: 10.1210/jc.2010-2137 | No data on or comparisons by ethnicity |
| Kunitz SJ. Sex, race and social role-history and the social determinants of health. International Journal of Epidemiology 2007;36(1):3-10. doi: 10.1093/ije/dyl296 | Wrong population (e.g. limited to population with a specific disease or condition and/or not based in the UK) |
| Lakasing E, Mirza ZA. The health of Britain's Polish migrants: A suitable case for history taking and examination. British Journal of General Practice 2009;59(559):138-39. doi: 10.3399/bjgp09X406992 | Wrong publication/study type (e.g. protocol, commentary, editorial, review) |
| Landman J, Cruickshank JK. A review of ethnicity, health and nutrition-related diseases in relation to migration in the United Kingdom. Public Health Nutrition 2001;4(2B):647-57. doi: 10.1079/phn2001148 | Wrong publication/study type (e.g. protocol, commentary, editorial, review) |
| Large P, Ghosh K. A methodology for estimating the population by ethnic group for areas within England. Population Trends 2006(123):21-31. PMID: 16619856 | No data on relative all-cause mortality or unable to estimate from available data |
| Launer J. Ethnic inequalities in health: Should we talk about implicit white supremacism? Postgraduate Medical Journal 2020;96(1132):117-18. doi: 10.1136/postgradmedj-2019-137295 | Wrong publication/study type (e.g. protocol, commentary, editorial, review) |
| Law MR, Morris JK. Why is mortality higher in poorer areas and in more northern areas of England and Wales? Journal of Epidemiology and Community Health 1998;52(6):344-52. doi: 10.1136/jech.52.6.344 | No data on or comparisons by ethnicity |
| Lay-Flurrie S, Mathieu E, Bankhead C, et al. Patient consultation rate and clinical and NHS outcomes: a cross-sectional analysis of English primary care data from 2.7 million patients in 238 practices. BMC Health Services Research 2019;19(1):219. doi: 10.1186/s12913-019-4036-y | No data on or comparisons by ethnicity |
| Lee A, Landau R. Racial and ethnic disparities uncovered in the 2020 MBRRACE-UK report - Global implications and future agenda. Anaesthesia, Critical care & Pain medicine 2021;40(2):100853. doi: 10.1016/j.accpm.2021.100853 | Wrong publication/study type (e.g. protocol, commentary, editorial, review) |
| Lee KK, Norris ET, Rishishwar L, et al. Ethnic disparities in mortality and group-specific risk factors in the UK Biobank. PLOS Global Public Health 2023;3(2):e0001560. doi: 10.1371/journal.pgph.0001560 | Same data source |
| Lester D. Consistency between death rates of immigrants and death rates in their home nations. Perceptual and Motor Skills 1992;75(3 Pt 2):1154. doi: 10.2466/pms.1992.75.3f.1154 | Wrong population (e.g. limited to population with a specific disease or condition and/or not based in the UK) |
| Li CP. Quality of life patterns and survival among older people. Journal of Nursing Research 2013;21(2):94-109. doi: 10.1097/jnr.0b013e3182921f9d | No data on or comparisons by ethnicity |
| Ling S, Xu G, Zaccardi F, et al. Kidney Function and Long-Term Risk of End-Stage Kidney Disease and Mortality in a Multiethnic Population. Kidney International Reports 2023;8(9):1761-71. doi: 10.1016/j.ekir.2023.06.014 | Wrong population (e.g. limited to population with a specific disease or condition and/or not based in the UK) |
| Lip GYH, Lane DA, Beevers DG. Ethnicity, blood pressure and all-cause mortality in the Birmingham Factory Screening Project. American Journal of Hypertension 2003;16(5):211A-12A. doi: 10.1016/s0895-7061(03)00650-2 | Same data source |
| Lister J. Inequalities in Health. New England Journal of Medicine 1980;303(22):1281-82. doi: 10.1056/NEJM198011273032207 | No data on or comparisons by ethnicity |
| Lord Hunt P. The future of public health. World Hospitals and Health Services 2002;38(3):34-40, 42, 44. PMID: 12602090 | Wrong publication/study type (e.g. protocol, commentary, editorial, review) |
| Macfarlane GJ, Beasley M, Jones GT, et al. The epidemiology of regular opioid use and its association with mortality: Prospective cohort study of 466 486 UK biobank participants. EClinicalMedicine 2020;21 doi: 10.1016/j.eclinm.2020.100321 | No data on or comparisons by ethnicity |
| Macintyre S. The patterning of health by social position in contemporary Britain: directions for sociological research. Social Science & Medicine 1986;23(4):393-415. doi: 10.1016/0277-9536(86)90082-1 | Wrong publication/study type (e.g. protocol, commentary, editorial, review) |
| Mackenbach JP, Kulhanova I, Menvielle G, et al. Trends in inequalities in premature mortality: a study of 3.2 million deaths in 13 European countries. Journal of Epidemiology and Community Health 2015;69(3):207-17; discussion 05-6. doi: 10.1136/jech-2014-204319 | No data on or comparisons by ethnicity |
| Maheswaran R, Strong M, Clifford P, et al. Socioeconomic deprivation, mortality and health of within-city migrants: A population cohort study. Journal of Epidemiology and Community Health 2018;72(6):519-25. doi: 10.1136/jech-2017-210166 | No data on or comparisons by ethnicity |
| Malmusi D. Immigrants' health and health inequality by type of integration policies in European countries. European Journal of Public Health 2015;25(2):293-99. doi: 10.1093/eurpub/cku156 | Ethnicity categories not sufficiently granular |
| Marmot MG, Adelstein AM, Bulusu L. Lessons from the study of immigrant mortality. Lancet 1984;1(8392):1455-7. doi: 10.1016/s0140-6736(84)91943-3 | Same data source |
| Marmot MG, Smith GD. Why are the Japanese living longer? British Medical Journal 1989;299(6715):1547-51. doi: 10.1136/bmj.299.6715.1547 | No data on or comparisons by ethnicity |
| Maxwell R, Harding S. Mortality of migrants from outside England and Wales by marital status. Population Trends 1998(91):15-22. PMID: 9575539 | Same data source |
| McCartney G, Russ TC, Walsh D, et al. Explaining the excess mortality in Scotland compared with England: pooling of 18 cohort studies. Journal of Epidemiology and Community Health 2015;69(1):20-27. doi: 10.1136/jech-2014-204185 | No data on or comparisons by ethnicity |
| McLoone P, Boddy FA. Deprivation and mortality in Scotland, 1981 and 1991. British Medical Journal 1994;309(6967):1465. doi: 10.1136/bmj.309.6967.1465 | No data on or comparisons by ethnicity |
| Measuring progress on health disparities. Lancet (London, England) 2006;367(9526):1876-76 | Wrong publication/study type (e.g. protocol, commentary, editorial, review) |
| Mindell J, Biddulph JP, Hirani V, et al. Cohort profile: The health survey for England. International Journal of Epidemiology 2012;41(6):1585-93. doi: 10.1093/ije/dyr199 | Wrong publication/study type (e.g. protocol, commentary, editorial, review) |
| Morris M, Woods LM, Rachet B. A novel ecological methodology for constructing ethnic-majority life tables in the absence of individual ethnicity information. Journal of Epidemiology and Community Health 2015;69(4):361-7. doi: 10.1136/jech-2014-204210 | Other (e.g. ecological not individual data) |
| Muennig P, Murphy M. Does racism affect health? Evidence from the United States and the United Kingdom. Journal of Health Politics, Policy and Law 2011;36(1):187-214. doi: 10.1215/03616878-1191153 | Wrong publication/study type (e.g. protocol, commentary, editorial, review) |
| Muilwijk M, Ho F, Waddell H, et al. Contribution of type 2 diabetes to all-cause mortality, cardiovascular disease incidence and cancer incidence in white Europeans and South Asians: findings from the UK Biobank population-based cohort study. BMJ Open Diabetes Research & Care 2019;7(1):e000765. doi: 10.1136/bmjdrc-2019-000765 [published Online First: 20191217] | Same data source |
| Nikoloudis D, Kountouras D, Hiona A. The frequency of combined IFITM3 haplotype involving the reference alleles of both rs12252 and rs34481144 is in line with COVID-19 standardized mortality ratio of ethnic groups in England. PeerJ 2020;8:e10402 | Same data source |
| Nimmo AW, Peterkin G, Coid DR. Monitoring mortality in general practice in Grampian, Scotland. Scottish Medical Journal 2004;49(2):66-68. doi: 10.1177/003693300404900209 | No data on or comparisons by ethnicity |
| Norman P, Boyle P, Rees P. Selective migration, health and deprivation: a longitudinal analysis. Social Science & Medicine 2005;60(12):2755-71. doi: 10.1016/j.socscimed.2004.11.8 | No data on or comparisons by ethnicity |
| Parry G, Van Cleemput P, Peters J, et al. Health status of Gypsies and Travellers in England. Journal of Epidemiology and Community Health 2007;61(3):198-204. doi: 10.1136/jech.2006.045997 | Other (e.g. ecological not individual data) |
| Patterson R, Panter J, Vamos EP, et al. Associations between commute mode and cardiovascular disease, cancer, and all-cause mortality, and cancer incidence, using linked Census data over 25 years in England and Wales: a cohort study. The Lancet Planetary Health 2020;4(5):e186-e94. doi: 10.1016/S2542-5196(20)30079-6 | Ethnicity categories not sufficiently granular |
| Pearson-Stuttard J, Bennett J, Cheng YJ, et al. Trends in predominant causes of death in individuals with and without diabetes in England from 2001 to 2018: an epidemiological analysis of linked primary care records. The Lancet Diabetes & Endocrinology 2021;9(3):165-73. doi: 10.1016/S2213-8587(20)30431-9 | No data on relative all-cause mortality or unable to estimate from available data |
| Pegington M, Harvie M, Harkness EF, et al. Obesity at age 20 and weight gain during adulthood increase risk of total and premature all-cause mortality: findings from women attending breast screening in Manchester. BMC Women's Health 2023;23(1):17. doi: 10.1186/s12905-023-02162-0 | Wrong population (e.g. limited to population with a specific disease or condition and/or not based in the UK) |
| Perkin MR, Heap S, Crerar-Gilbert A, et al. Deaths in people from Black, Asian and minority ethnic communities from both COVID-19 and non-COVID causes in the first weeks of the pandemic in London: a hospital case note review. BMJ Open 2020;10(10):e040638. doi: 10.1136/bmjopen-2020-040638 | Ethnicity categories not sufficiently granular |
| Phillimore P, Beattie A, Townsend P. Widening inequality of health in Northern England, 1981-91. British Medical Journal 1994;308(6937):1125-28. doi: 10.1136/bmj.308.6937.1125 | No data on or comparisons by ethnicity |
| Platt L, Warwick R. COVID-19 and Ethnic Inequalities in England and Wales*. Fiscal Studies 2020;41(2):259-89. doi: 10.1111/1475-5890.12228 | No data on relative all-cause mortality or unable to estimate from available data |
| Rafnsson SB, Bhopal RS. Large-scale epidemiological data on cardiovascular diseases and diabetes in migrant and ethnic minority groups in Europe. European Journal of Public Health 2009;19(5):484-91. doi: 10.1093/eurpub/ckp073 | Wrong publication/study type (e.g. protocol, commentary, editorial, review) |
| Raftery J, Jones DR, Rosato M. The mortality of first and second generation Irish immigrants in the U.K. Social Science & Medicine 1990;31(5):577-84. doi: 10.1016/0277-9536(90)90093-8 | Same data source |
| Raynor P, Born Bradford Collaborative G. Born in Bradford, a cohort study of babies born in Bradford, and their parents: Protocol for the recruitment phase. BMC Public Health 2008;8 doi: 10.1186/1471-2458-8-327 | Wrong publication/study type (e.g. protocol, commentary, editorial, review) |
| Rees PH, Wohland PN, Norman PD. The estimation of mortality for ethnic groups at local scale within the United Kingdom. Social Science & Medicine 2009;69(11):1592-607. doi: 10.1016/j.socscimed.2009.8.15 | Other (e.g. ecological not individual data) |
| Reus-Pons M, Kibele EUB, Janssen F. Differences in healthy life expectancy between older migrants and non-migrants in three European countries over time. International Journal of Public Health 2017;62(5):531-40. doi: 10.1007/s00038-017-0949-6 | Ethnicity categories not sufficiently granular |
| Riva M, Curtis S, Norman P. Residential mobility within England and urban-rural inequalities in mortality. Social Science & Medicine 2011;73(12):1698-706. doi: 10.1016/j.socscimed.2011.9.30 | Ethnicity categories not sufficiently granular |
| Rothstein WG. The decrease in socioeconomic differences in mortality from 1920 to 2000 in the United States and England. Journal of the History of Medicine and Allied Sciences 2012;67(4):515-52. doi: 10.1093/jhmas/jrr031 | No data on or comparisons by ethnicity |
| Scally G. 'The very pests of society': the Irish and 150 years of public health in England. Clinical Medicine 2004;4(1):77-81. doi: 10.7861/clinmedicine.4-1-77 | Wrong publication/study type (e.g. protocol, commentary, editorial, review) |
| Schofield L, Walsh D, Feng Z, et al. Does ethnic diversity explain intra-UK variation in mortality? A longitudinal cohort study. BMJ Open 2019;9(3):e024563. doi: 10.1136/bmjopen-2018-024563 | Ethnicity categories not sufficiently granular |
| Schofield L, Walsh D, Feng Z, et al. Does ethnic diversity explain intra-UK variation in mortality? A longitudinal cohort study. BMJ Open 2019;9(3):e024563. doi: 10.1136/bmjopen-2018-024563 | Ethnicity categories not sufficiently granular |
| Seaman R, Leyland AH, Popham F. How have trends in lifespan variation changed since 1950? A comparative study of 17 Western European countries. European Journal of Public Health 2016;26(2):360-62. doi: 10.1093/eurpub/ckv185 | Wrong population (e.g. limited to population with a specific disease or condition and/or not based in the UK) |
| Shah AD, Thornley S, Chung S-C, et al. White cell count in the normal range and short-term and long-term mortality: international comparisons of electronic health record cohorts in England and New Zealand. BMJ Open 2017;7(2) doi: 10.1136/bmjopen-2016-013100 | Other (e.g. ecological not individual data) |
| Shantsila E, Shantsila A, Williams N, et al. Community echocardiographic left ventricular hypertrophy and mortality in South Asians in the UK: E-ECHOES study. European Journal of Preventive Cardiology 2023;30(Supplement 1):i287. doi: 10.1093/eurjpc/zwad125.219 | Wrong population (e.g. limited to population with a specific disease or condition and/or not based in the UK) |
| Shantsila E, Shantsila A, Williams N, et al. Left ventricular hypertrophy and mortality in ethnic minority groups in the UK: e-ECHOES study. Journal of Hypertension 2024;42(1):95-100. doi: 10.1097/HJH.0000000000003561 | Wrong population (e.g. limited to population with a specific disease or condition and/or not based in the UK) |
| Shibata D, Tillin T, Beauchamp N, et al. Cerebral infarcts in blacks vs whites: The Southall and Brent revisited (SABRE) multi-ethnic cohort study. Stroke Conference 2012;43(2 Meeting Abstracts) | Other (e.g. ecological not individual data) |
| Smith GD, Blane D, Bartley M. Explanations for socio-economic differentials in mortality: Evidence from Britain and elsewhere. European Journal of Public Health 1994;4(2):131-44. doi: 10.1093/eurpub/4.2.131 | No data on or comparisons by ethnicity |
| Socioeconomic mortality differentials. Statistical bulletin (Metropolitan Life Insurance Company) 1975;56:2-5 | No data on or comparisons by ethnicity |
| Soedamah-Muthu SS, De Neve M, Shelton NJ, et al. Joint associations of alcohol consumption and physical activity with all-cause and cardiovascular mortality. The American Journal of Cardiology 2013;112(3):380-6. doi: 10.1016/j.amjcard.2013.3.40 | Ethnicity categories not sufficiently granular |
| Steptoe A, Jackson SE. Association of Noncognitive Life Skills With Mortality at Middle and Older Ages in England. JAMA Network Open 2020;3(5):e204808. doi: 10.1001/jamanetworkopen.2020.4808 | Ethnicity categories not sufficiently granular |
| Sun YR, Hu XK, Xie J. Spatial inequalities of COVID-19 mortality rate in relation to socioeconomic and environmental factors across England. Science of the Total Environment 2021;758 doi: 10.1016/j.scitotenv.2020.143595 | Other (e.g. ecological not individual data) |
| Sundquist J, Bajekal M, Jarman B, et al. Underprivileged area score, ethnicity, social factors and general mortality in district health authorities in England and Wales. Scandinavian Journal of Primary Health Care 1996;14(2):79-85. doi: 10.3109/02813439608997075 | Ethnicity categories not sufficiently granular |
| Swerdlow AJ. Mortality and cancer incidence in Vietnamese refugees in England and Wales: A follow-up study. International Journal of Epidemiology 1991;20(1):13-19. doi: 10.1093/ije/20.1.13 | Other (e.g. ecological not individual data) |
| Teagle WL, Norris ET, Rishishwar L, et al. Comorbidities and ethnic health disparities in the UK biobank. JAMIA OPEN 2022;5(3) doi: 10.1093/jamiaopen/ooac057 | No data on relative all-cause mortality or unable to estimate from available data |
| The Black report. British Medical Journal (Clinical research ed) 1990;301(6752):608-9. PMID: 2242466 | No data on or comparisons by ethnicity |
| Tillin T, Forouhi NG, McKeigue PM, et al. Southall And Brent REvisited: Cohort profile of SABRE, a UK population-based comparison of cardiovascular disease and diabetes in people of European, Indian Asian and African Caribbean origins. International Journal of Epidemiology 2012;41(1):33-42. doi: 10.1093/ije/dyq175 | Wrong publication/study type (e.g. protocol, commentary, editorial, review) |
| Tunstall H, Mitchell R, Gibbs J, et al. Socio-demographic diversity and unexplained variation in death rates among the most deprived parliamentary constituencies in Britain. Journal of Public Health (Oxford) 2012;34(2):296-304. doi: 10.1093/pubmed/fdr078 | Ethnicity categories not sufficiently granular |
| Wallace M, Darlington‐Pollock F. Poor health, low mortality? Paradox found among immigrants in England and Wales. Population, Space and Place 2020;28(3) doi: 10.1002/psp.2360 | Same data source |
| Wallace M, Kulu H. Can the salmon bias effect explain the migrant mortality advantage in England and Wales? Population Space and Place 2018;24(8) doi: 10.1002/psp.2146 | Same data source |
| Wallace M, Kulu H. Migration and Health in England and Scotland: a Study of Migrant Selectivity and Salmon Bias. Population Space and Place 2014;20(8):694-708. doi: 10.1002/psp.1804 | No data on relative all-cause mortality or unable to estimate from available data |
| Wallace MJ. Mortality among immigrants and their descendants in England and Wales. Thesis 2016. Department of Geography and Planning, School of Environmental Sciences. University of Liverpool. | Same data source |
| Walsh D, Buchanan D, Douglas A, et al. Increasingly Diverse: the Changing Ethnic Profiles of Scotland and Glasgow and the Implications for Population Health. Applied Spatial Analysis and Policy 2019;12(4):983-1009. doi: 10.1007/s12061-018-9281-7 | Same data source |
| Ward I, Robertson C, Agrawal U, et al. Risk of COVID-19 death in adults who received booster COVID-19 vaccinations: national retrospective cohort study on 14.6 million people in England. medRxiv 2023((Ward, Nafilyan) Office for National Statistics, Newport, United Kingdom(Robertson) Department of Mathematics and Statistics, Strathclyde University, Glasgow, United Kingdom(Robertson) Public Health Scotland, Glasgow, United Kingdom(Agrawal, de Lusignan) doi: 10.1101/2023.07.03.23291596 | No data on relative all-cause mortality or unable to estimate from available data |
| Williams R, Bhopal R, Hunt K. Health of a Punjabi ethnic-minority in Glasgow - a comparison with the general-population. Journal of Epidemiology and Community Health 1993;47(2):96-102. doi: 10.1136/jech.47.2.96 | No data on relative all-cause mortality or unable to estimate from available data |
| Williams R, Ecob R. Regional mortality and the Irish in Britain: Findings from the ONS longitudinal study. Sociology of Health and Illness 1999;21(3):344-67. doi: 10.1111/1467-9566.160 | Same data source |
| Wohland P, Rees P, Nazroo J, et al. Inequalities in healthy life expectancy between ethnic groups in England and Wales in 2001. Ethnicity & Health 2015;20(4):341-53. doi: 10.1080/13557858.2014.921892 | Other (e.g. ecological not individual data) |
| Wohland P, Rees P. Life Expectancy Variation across England's Local Areas by Ethnic Group in 2001. Journal of Maps 2010;6(1):354-59. doi: 10.4113/jom.2010.1110 | Other (e.g. ecological not individual data) |
| Wood A, Denholm R, Hollings S, et al. Linked electronic health records for research on a nationwide cohort of more than 54 million people in England. British Medical Journal 2021;372 doi: 10.1136/bmj.n826 | Wrong publication/study type (e.g. protocol, commentary, editorial, review) |
| Wright J, Small N, Raynor P, et al. Cohort profile: The born in Bradford multi-ethnic family cohort study. International Journal of Epidemiology 2013;42(4):978-91. doi: 10.1093/ije/dys112 | Wrong publication/study type (e.g. protocol, commentary, editorial, review) |
| Yates T, Bakrania K, Zaccardi F, et al. Reaction time, cardiorespiratory fitness and mortality in UK Biobank: An observational study. Intelligence 2018;66:79-83. doi: 10.1016/j.intell.2017.11.6 | No data on or comparisons by ethnicity |
| Yates T, Zaccardi F, Dhalwani NN, et al. Association of walking pace and handgrip strength with all-cause, cardiovascular, and cancer mortality: a UK Biobank observational study. European Heart Journal 2017;38(43):3232-40. doi: 10.1093/eurheartj/ehx449 | Ethnicity categories not sufficiently granular |

# Supplement 4: Methods to combine study results

The detailed methods utilised for combining estimates and calculating the effect using age-standardized mortality rates are provided below.

Combining estimates

We utilized a random-effects meta-analysis approach to combine the results, with the formulas presented below:

$Pooled Estimate=\frac{\sum_{i=1}^{k} w_{i} \times{Estimate}_{i}}{\sum_{i=1}^{k} w_{i}}$ ,

where ${Estimate}_{i}$ represents the effect size estimate from each study, $w_{i}$ represents the weight assigned to each study, which accounts for both within-study and between-study variability, *k* is the total number of studies to combine.

The weights $w_{i}$ can be calculated using the DerSimonian and Laird method^1^:

$w_{i}= \frac{1}{{Var}_{i}+ \tau^{2}}$ ,

where ${Var}_{i}$is the variance of the effect size estimate in study *i* , $\tau^{2}$ is the estimated between-study variance. To avoid a unit-of-analysis error, we adjust the variance by applying the variance inflation factor^2^:

$${Var}_{adj}= {Var}_{unadj}*k/2$$

Estimating relative measures and its confidence intervals from the age standardised mortality rates (ASMR)

The relative risk (RR) is the ratio of the risk of mortality in one group to the risk in another group. The formula to calculate RR is:

$RR=\frac{{ASMR}_{2}}{{ASMR}_{1}}$ ,

where ${ASMR}_{1}$ is the reference group.

The formula to calculate the confidence interval for RR is:

$${CI}_{RR}= e^{ln(RR)\pm Z\times{SE}_{ln(rr)}}$$

where *ln*(*RR*) is the natural log of the relative risk, *Z* is the Z-score corresponding to the desired level of confidence (1.96 for 95% confidence interval), ${SE}_{ln(rr)}$ is the standard error of the natural log of the relative risk.

The standard error of the natural log of the relative risk ${SE}_{ln(rr)}$can be calculated using the following formula:

$${SE}_{ln(rr)}=\sqrt{\frac{1}{{ASMR}_{1}}+\frac{1}{{ASMR}_{2}}}$$

Estimating relative measures and its confidence intervals from Standardized Mortality Ratio (SMR)

When having number of observed deaths and SMR, using the above formula we could calculate number of expected deaths.

$$SMR=\frac{Number of observed deaths}{Number of expected deaths}$$

Then CIs could then be calculated using Vandenbroucke method^3^:

$$CI=\frac{\left( \sqrt{Number of observed deaths} \pm Z\times0.5 \right)^{2}}{Number of expected deaths}$$

where Z is the Z-score corresponding to the desired level of confidence (1.96 for 95% confidence interval).

References:

1. DerSimonian R, Laird N. Meta-analysis in clinical trials. *Control Clin Trials*. 1986;7(3):177-188. doi:10.1016/0197-2456(86)90046-2

2. Rücker G, Cates CJ, Schwarzer G. Methods for including information from multi-arm trials in pairwise meta-analysis. *Res Synth Methods*. 2017;8(4):392-403. doi:10.1002/jrsm.1259

3. Ulm K. A simple method to calculate the confidence interval of a standardized mortality ratio (SMR). *Am J Epidemiol*. 1990;131(2):373-375. doi:10.1093/oxfordjournals.aje.a115507

# Supplement 5: Table of characteristics for included studies

| Study citation | Location | Study design | Baseline recruitment | Mortality follow-up | Total study population (n) | Data source | Age range (year) | Ethnicity measure | Ethnic groups as defined by study | Ethnic groups as included in analysis |
| --- | --- | --- | --- | --- | --- | --- | --- | --- | --- | --- |
| Abbotts *et al.* 1999 | West Scotland | Cohort | 1970 - 1973 | 1973 - 1994 | 5545 | The Collaborative Study | ≥ 35 | Irish surname | Irish,  Non-Irish *(ref.)* | White Irish |
| Balarajan & Bulusu 1990 | England & Wales | Unlinked census and death registry | 1981 Census | 1979 - 1983 | 49 154 693 | 1981 Census, death registry | ≥ 20 | Country of birth | Indian subcontinent,  African CW,  Caribbean CW,  Poland,  All Ireland,  Scotland,  Whole population *(ref.)* | South Asian,  Black African,  Black Caribbean,  Polish,  White Irish,  White Scottish |
| Balarajan & Raleigh 1997 | England & Wales | Unlinked census and death registry | 1991 Census | 1988 - 1992 | NR | 1991 Census, death registry | 20 - 69 | Country of birth | Bangladesh,  Whole population *(ref.)* | Bangladeshi |
| Bhaskaran *et al.* 2021 | England | Cohort (Primary Care) | 1 February 2019 & 1 February 2020 | 1 February - 9 November (2019 & 2020) | 17 456 515 | SystmOne^®^ & death registry (Open-SAFELY) | ≥ 18 | Self-reported ethnicity | South Asian,  Black,  Mixed,  White *(ref.)* | South Asian,  Black,  Mixed |
| Bhopal *et al.* 2018 | Scotland | Cohort (Census Linkage) | 2001 Census | 1 May 2001 - 30 April 2013 | 4 607 393 | 2001 Census & death registry | All ages | Self-reported ethnicity | Bangladeshi,  Indian,  Pakistani,  African,  Caribbean,  Chinese,  Mixed,  White Irish,  White Scottish *(ref.)* | Bangladeshi,  Indian,  Pakistani,  Black African,  Black Caribbean,  Chinese,  Mixed,  White Irish |
| Carey *et al.* 2021 | England | Cohort (Primary Care) | 18 March - 19 May (in each of 2015 - 2020) | 18 March - 19 May (in each of 2015 - 2020) | 4 835 708 | CPRD Aurum (EMIS^®^) | 30 - 104 | Self-reported ethnicity | Asian,  Black,  Mixed,  White *(ref.)* | South Asian,  Black,  Mixed |
| Connolly *et al.* 2011 | Northern Ireland | Cohort (Census Linkage) | 2001 Census | 2001 - 2007 | 930 219 | 2001 Census & death registry | 25 - 74 | Country of birth | Scotland,  Northern Ireland *(ref.)* | White Scottish |
| Eastwood *et al.* 2019 | North-West London (England) | Cohort | 1988 - 1991 | 1988 - 2015 | 1961 | Southall And Brent REvisited (SABRE) study | ≥ 40 | Self-reported ethnicity | South Asian,  White European *(ref.)* | South Asian |
| Fischbacher *et al.* 2007 | Scotland | Unlinked census and death registry | 1991 & 2001 Census | 1 January 1997 - 31 March 2003 | NR | 1991/2001 Census, death registry | ≥ 25 | Country of birth | Bangladesh,  India,  Pakistan,  China,  Hong-Kong,  Northern Ireland,  Republic of Ireland,  Scotland,  England & Wales *(ref.)* | Bangladeshi,  Indian,  Pakistani,  Chinese,  White Irish,  White Scottish |
| Gill *et al.* 2007 | England & Wales | Unlinked census and death registry | 1991 Census | 1989 - 1992 | NR | 1991 Census, death registry | All ages | Country of birth | Bangladesh,  India,  Pakistan,  West & South Africa,  Caribbean,  China,  Hong-Kong,  Taiwan,  Whole population *(ref.)* | Bangladeshi,  Indian,  Pakistani,  Black African,  Black Caribbean  Chinese, |
| Harding & Balarajan 1996 | England & Wales | Cohort | 1971 Census | 1971 - 1989 | NR | ONS Longitudinal Study | ≥ 15 | Country of birth of parents | Second generation Irish,  Whole population *(ref.)* | White Irish |
| Harding *et al.* 1997 | England & Wales | Unlinked census and death registry | 1991 Census | 1991 - 1993 | NR | 1991 Census, death registry | 20 - 64 | Country of birth | Bangladesh,  India,  Pakistan,  Indian subcontinent,  West & South Africa,  Caribbean,  All Ireland,  Scotland,  Whole population *(ref.)* | Bangladeshi,  Indian,  Pakistani,  South Asian,  Black African,  Black Caribbean,  White Irish,  White Scottish |
| Harding & Balarajan 2001 | England & Wales | Cohort | 1971 Census | 1971 - 1997 | 358 012 | ONS Longitudinal Study | NR | Country of birth of person, parents and grandparents | First, second or third generation Irish,  Other *(ref.)* | White Irish |
| Hayes *et al.* 2017 | Newcastle upon Tyne (England) | Cohort | 1993 - 1997 | To 31 May 2014 | 1501 | Newcastle Heart Project | ≥ 25 | Self-reported ethnicity | Indian,  Pakistani + Bangladeshi (combined),  South Asian,  European (*ref*.) | Bangladeshi,  Indian,  Pakistani,  South Asian |
| Hippisley-Cox & Coupland 2017 | England | Cohort (Primary Care) | 1 January 2012 - 30 September 2016 | 1 January 2012 - 30 September 2016 | 1 466 598 | QResearch (EMIS^®^) | 65 - 100 | Self-reported ethnicity | Bangladeshi,  Indian,  Pakistani,  Black African,  Caribbean,  Chinese,  White or not recorded *(ref.)* | Bangladeshi,  Indian,  Pakistani  Black African,  Black Caribbean,  Chinese, |
| Ikram *et al.* 2016 | England & Wales | Unlinked census and death registry | 2001 Census | 1999 - 2003 | NR | 2001 Census, death registry | ≤ 69 | Country of birth | Indian subcontinent,  Sub-Saharan Africa,  Caribbean,  East Asia,  Eastern Europe,  England & Wales *(ref.)* | South Asian  Black African,  Black Caribbean,  Chinese,  Polish, |
| Jesky *et al.* 2013 | West Midlands (England) | Cohort (Primary Care) | May 2008 | May 2008 - February 2011 | 31 254 | Primary Care Mortality Database | ≥ 40 | Self-reported ethnicity | South Asian,  Black,  White *(ref.)* | South Asian,  Black |
| Lane *et al.* 2005 | Birmingham (England) | Cohort | 1979 - 1986 | 1979 - 31 December 2003 | 2624 | Birmingham Factory Screening Project | NR | Self-reported ethnicity | African-Caribbean,  White European *(ref.)* | Black Caribbean |
| Marmot *et al.* 1984 | England &  Wales | Unlinked census and death registry | 1971 Census | 1970 - 1972 | NR | 1971 Census, death registry | ≥ 15 | Country of birth | Indian subcontinent,  African CW,  Caribbean CW,  Poland,  All Ireland,  Scotland,  Whole population *(ref.)* | South Asian,  Black African,  Black Caribbean,  Polish,  White Irish,  White Scottish |
| Millard *et al.* 2015 | Scotland | Cohort | 1991 Census | 1991 - 31 December 2009 | NR | Scottish Longitudinal Study | ≤ 64 | Self-reported ethnicity | Asian (excluding Chinese),  White *(ref.)* | South Asian |
| ONS 2021 | England & Wales | Cohort (Census Linkage) | 2011 Census | 2012 - 2019 | 50 189 388 | 2011 Census & death registry | ≥ 10 | Self-reported ethnicity | Bangladeshi,  Indian,  Pakistani,  Black African,  Black Caribbean,  Mixed,  White *(ref.)* | Bangladeshi,  Indian,  Pakistani,  Black African,  Black Caribbean,  Mixed |
| ONS 2023 | England & Wales | Cohort (Census Linkage) | 2021 Census | 2021 - 2023 | NR | 2021 Census & death registry | All ages | Self-reported ethnicity | Bangladeshi,  Indian,  Pakistani,  Black African,  Black Caribbean,  Chinese,  Mixed,  White *(ref.)* | Bangladeshi,  Indian,  Pakistani,  Black African,  Black Caribbean,  Chinese,  Mixed |
| Popham *et al.* 2010 | England, Scotland & Wales | Unlinked census and death registry | 2001 Census | 2000 - 2002 | 35 298 779 | 2001 Census, death registry | ≥ 25 | Country of birth | Scotland,  England & Wales *(ref.)* | White Scottish |
| Scott & Timæus 2013 | England & Wales | Cohort | 1991 Census | To 2005 | 436 195 | ONS Longitudinal Study | 14 - 79 | Self-reported ethnicity | Bangladeshi,  Indian,  Pakistani,  Black African,  Black Caribbean,  Chinese,  White *(ref.)* | Bangladeshi,  Indian,  Pakistani,  Black African,  Black Caribbean,  Chinese, |
| Stafford *et al.* 2022 | England | Cohort (Primary are) | 1 January 2015 | 1 January 2015 - 31 December 2019 | 532 059 | CPRD Aurum (EMIS^®^) | ≥ 18 | Self-reported ethnicity | Bangladeshi,  Indian,  Pakistani,  Black African,  Black Caribbean,  Chinese,  Mixed,  White *(ref.)* | Bangladeshi,  Indian,  Pakistani,  Black African,  Black Caribbean,  Chinese,  Mixed |
| Strongman *et al.* 2022 | United Kingdom | Cohort (Primary Care) | 5 March 2015 - 31 July 2020 | 5 March 2015 - 31 July 2020 | 9 635 613 | CPRD Gold & Aurum (EMIS^®^, Vision^®^) | ≥ 40 | Self-reported ethnicity | South Asian,  Black,  White *(ref.)* | South Asian,  Black |
| Wallace & Kulu 2014 | England & Wales | Cohort | Census: 1971, 1981, 1991, 2001 | 1971 - 2001 | 453 352 | ONS Longitudinal Study | 20 - 75 | Country of birth | Bangladesh,  India,  Pakistan,  West & Central Africa,  Jamaica,  Other Caribbean,  Scotland,  England & Wales *(ref.)* | Bangladeshi,  Indian,  Pakistani,  Black African,  Black Caribbean,  White Scottish, |
| Wallace & Kulu 2015 | England & Wales | Cohort | Census: 1971, 1981, 1991, 2001, 2011 | April 1971 - December 2012 | 591 724 | ONS Longitudinal Study | 20 - 86 | Country of birth | Bangladesh,  India,  Pakistan,  West and Central Africa,  Jamaica,  Other Caribbean,  China,  Eastern Europe,  Northern Ireland,  Republic of Ireland,  Scotland,  England & Wales *(ref.)* | Bangladeshi,  Indian,  Pakistani,  Black African,  Black Caribbean,  Chinese,  Polish,  White Irish,  White Scottish |
| Wallace 2016 | England & Wales | Cohort | Census: 1971, 1981, 1991, 2001, 2011 | April 1991 - December 2012 | 555 111 | ONS Longitudinal Study | 20 - 86 | Self-reported ethnicity | Indian,  Black African,  Black Caribbean,  Mixed: Asian/White,  Mixed: Black/White,  White: born in England & Wales *(ref.)* | Indian,  Black African,  Black Caribbean,  Mixed |
| Wild & McKeigue 1997 | England & Wales | Unlinked census and death registry | 1991 Census | 1989 - 1992 | 31 704 859 | 1991 Census, death registry | 20 - 69 | Country of birth | South Asia,  Ireland,  Scotland,  Whole population *(ref.)* | South Asian,  White Irish,  White Scottish |
| Wild *et al.* 2007 | England & Wales | Unlinked census and death registry | 2001 Census | 2001 - 2003 | NR | 2001 Census, death registry | ≥ 20 | Country of birth | Bangladesh,  India,  Pakistan,  China and Hong-Kong,  Ireland,  Scotland,  Whole population *(ref.)* | Bangladeshi,  Indian,  Pakistani,  Chinese,  White Irish,  White Scottish |
| Wright *et al.* 2017 | England | Cohort (Primary Care) | 1 January 1998 - 31 March 2015 | 1 January 1998 - 31 March 2015 | 908 016 | CPRD | NR | Self-reported ethnicity | South Asian,  Black,  White *(ref.)* | South Asian,  Black |
| Unpublished cohort study data | | | | | | | | | | |
| EPIC-Norfolk | Norfolk (England) | Cohort | 1993 - 1998 | 1993 - 2015 | NR | EPIC Norfolk study | NR | Self-reported ethnicity | Indian,  Pakistani,  Black Caribbean  White (ref.) | Indian, Pakistani, Black Caribbean |
| Million Women Study | England & Scotland | Cohort | 1996 - 2001 | 1996 - 2017 | NR | Million Women study | 50 - 82 | Self-reported ethnicity | Asian,  Black+other*  White (ref.) | Black Caribbean |
| UK Biobank | United Kingdom | Cohort | 2006 - 2010 | 2006 - 2022 | NR | UK Biobank study | 40 - 81 | Self-reported ethnicity | Bangladeshi,  Indian,  Pakistani,  Asian or Asian British,  Black African,  Black Caribbean,  Black or Black British,  Chinese,  Mixed,  White Irish,  White British (ref.) | Bangladeshi,  Indian,  Pakistani,  South Asian,  Black African,  Black Caribbean,  Black,  Chinese,  Mixed,  White Irish |

**Abbreviations*:*** *ref.* reference population; *CW* Commonwealth; *NR* not reported; *NHS* National Health Service; *ONS* Office for National Statistics; *CPRD* Clinical Practice Research Datalink; *EMIS*^®^ Egton Medical Information System

*The majority of participants in the Black + other ethnicity category were of Black Caribbean ethnicity so they were analysed in this group in the meta-analysis.

# Supplement 6: Age-adjusted all-cause mortality by ethnicity in males


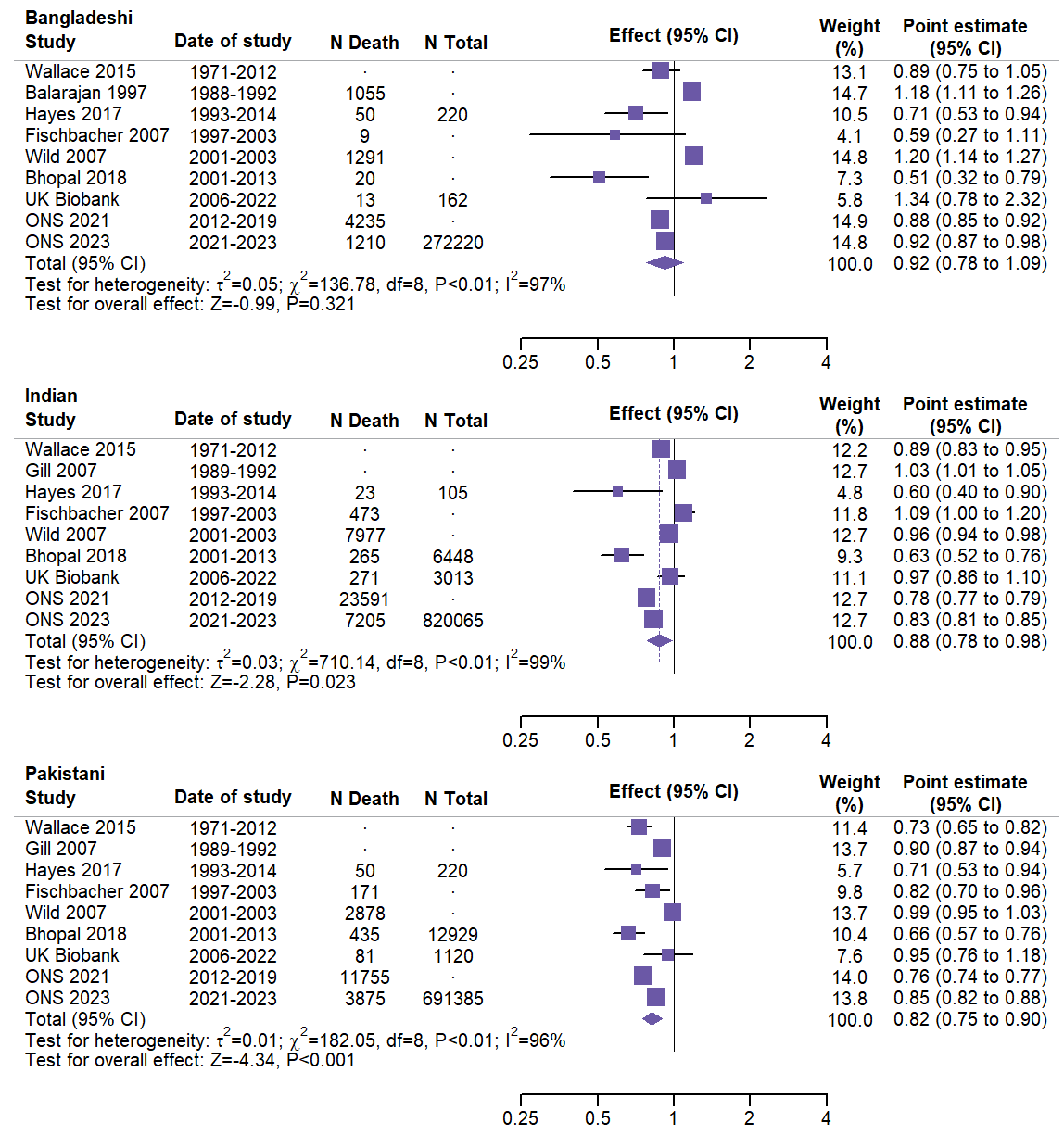


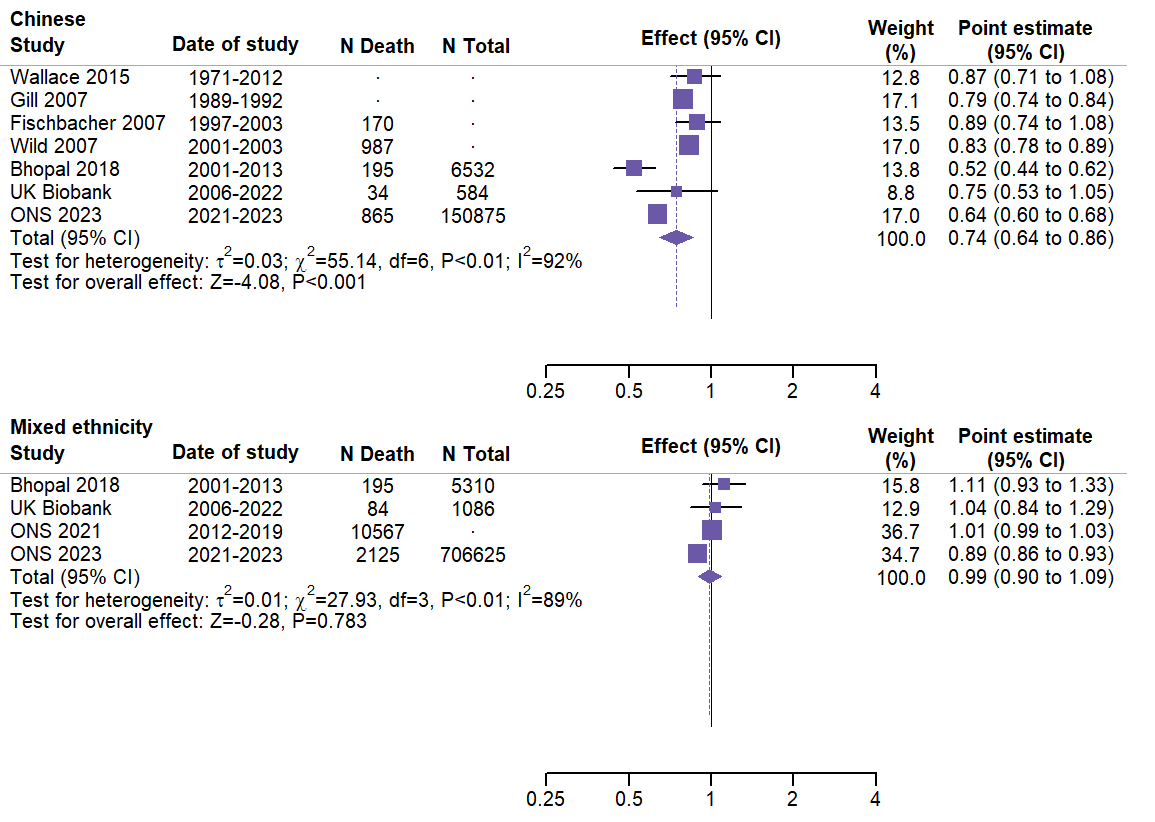

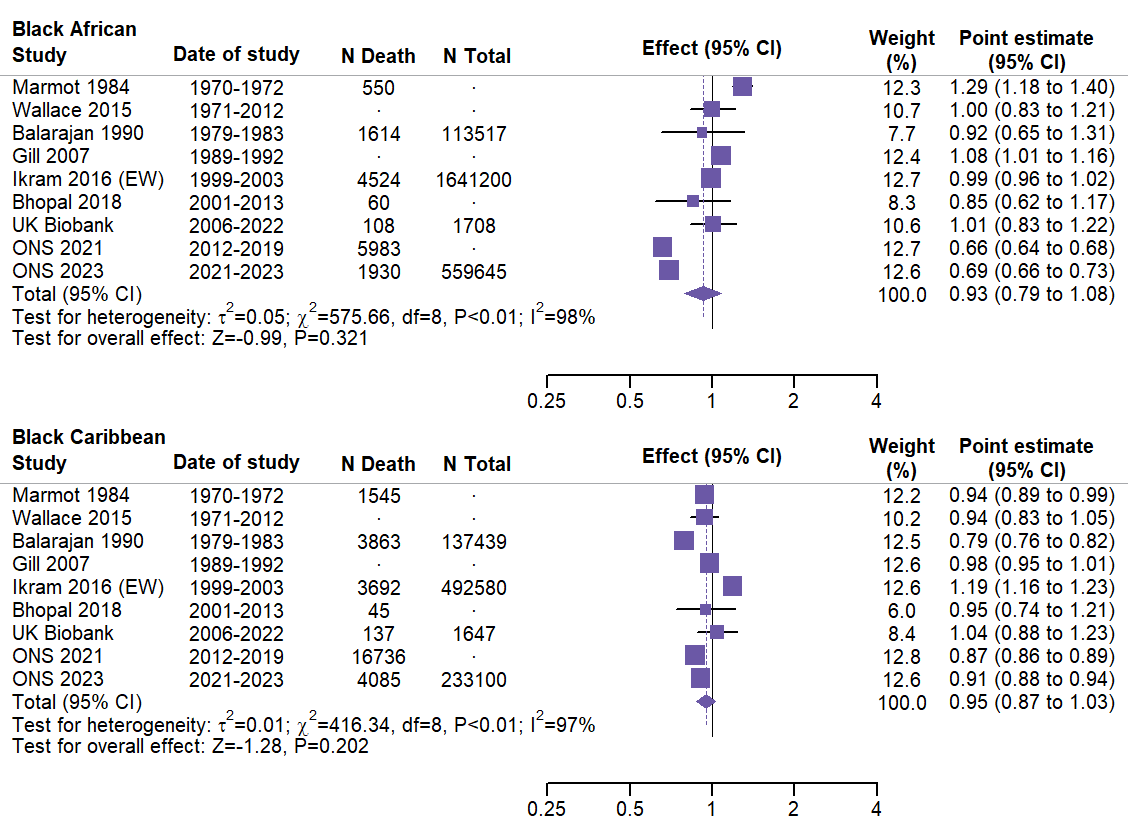


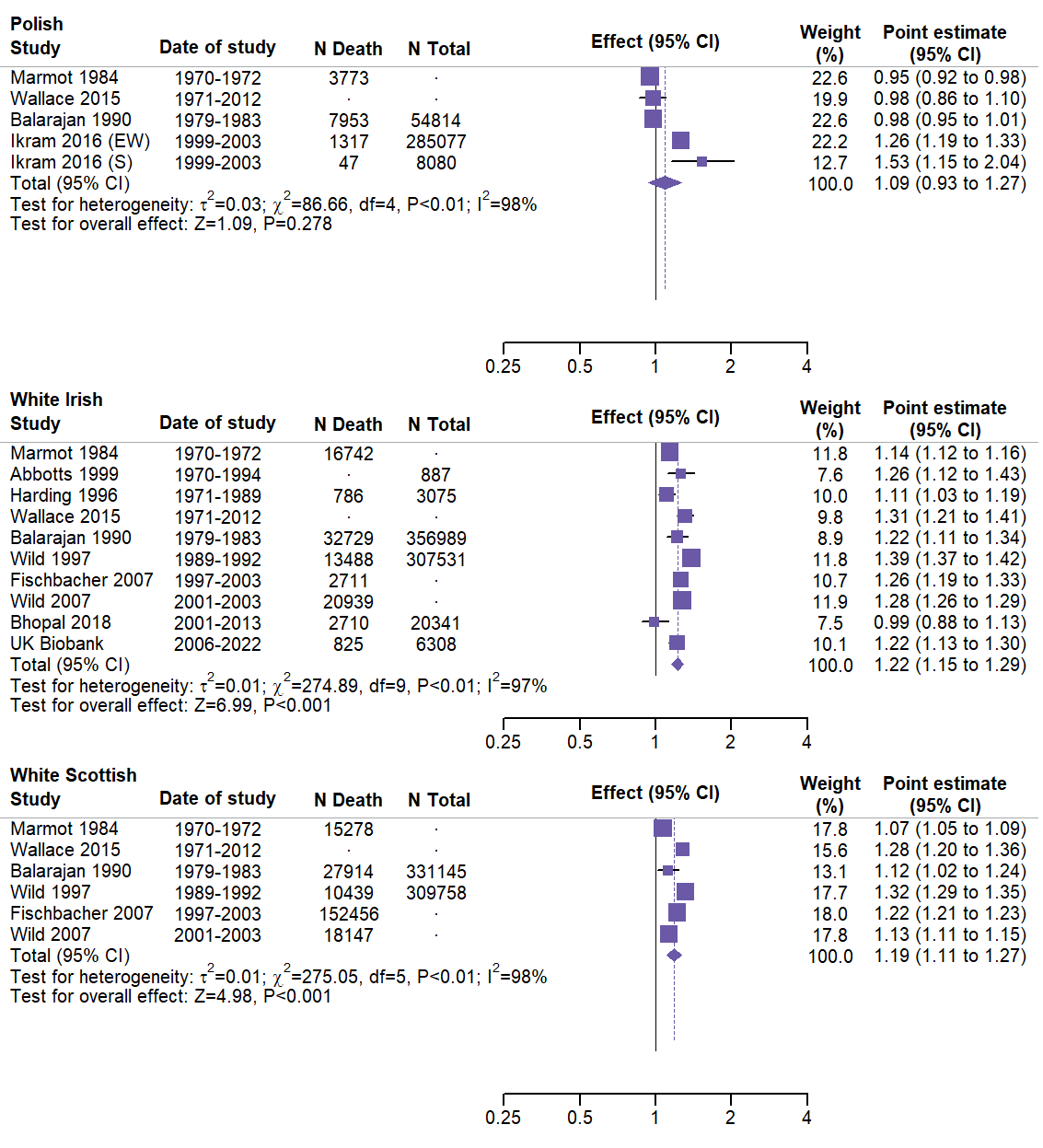


Note: All included studies had a White English/Welsh or total population comparator apart from the following studies with a White Scottish comparator: Bhopal 2018 and Ikram 2016 (S).

# Supplement 7: Age-adjusted all-cause mortality by ethnicity in females


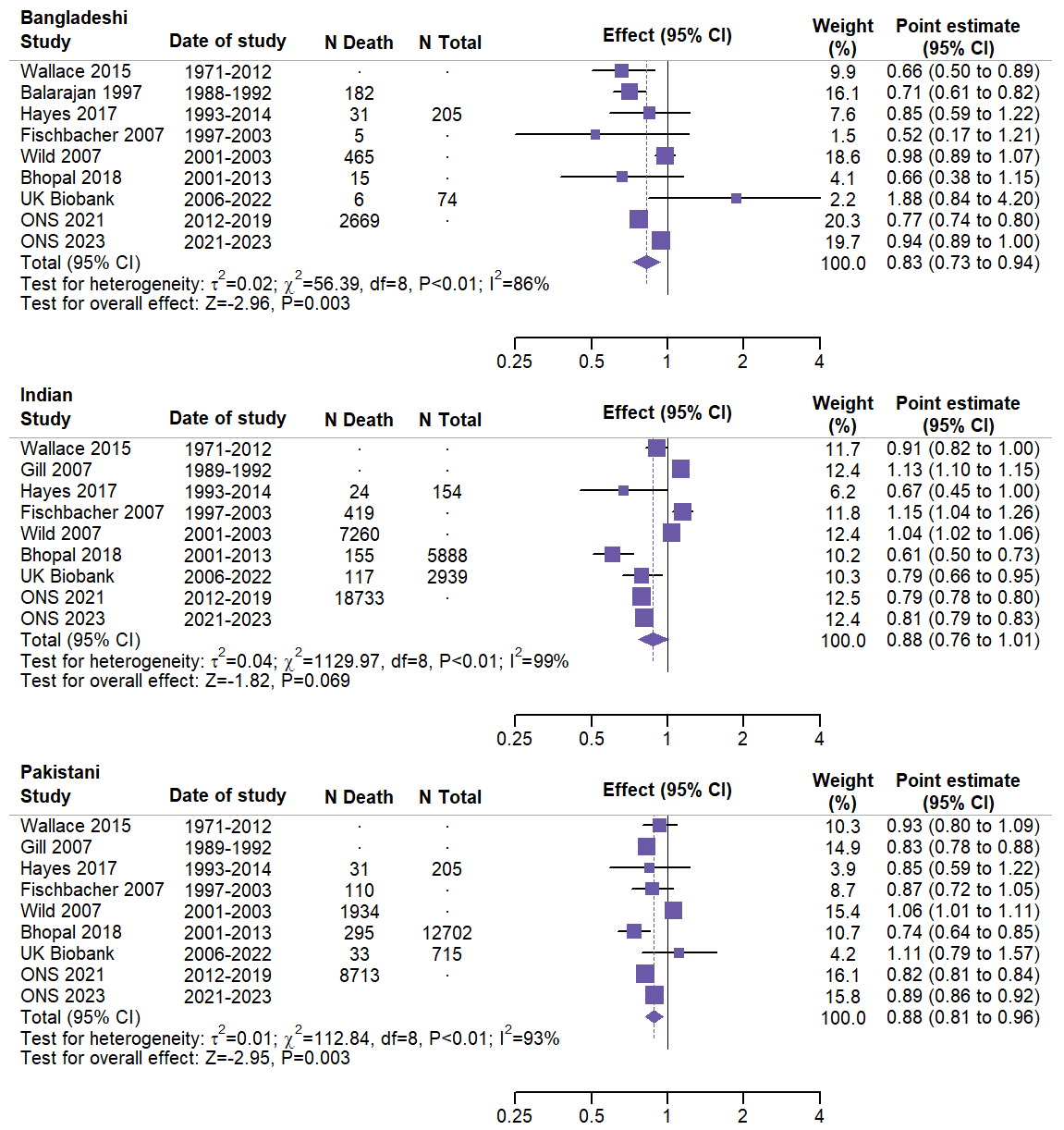


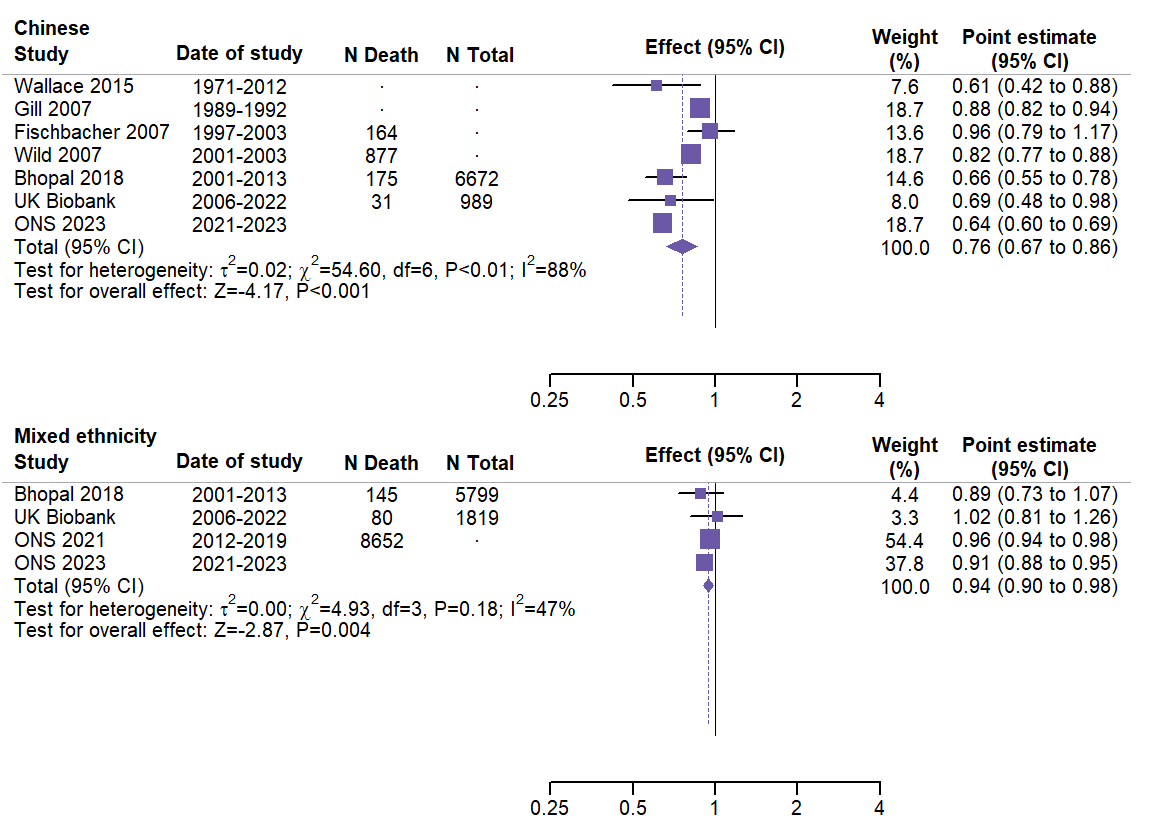

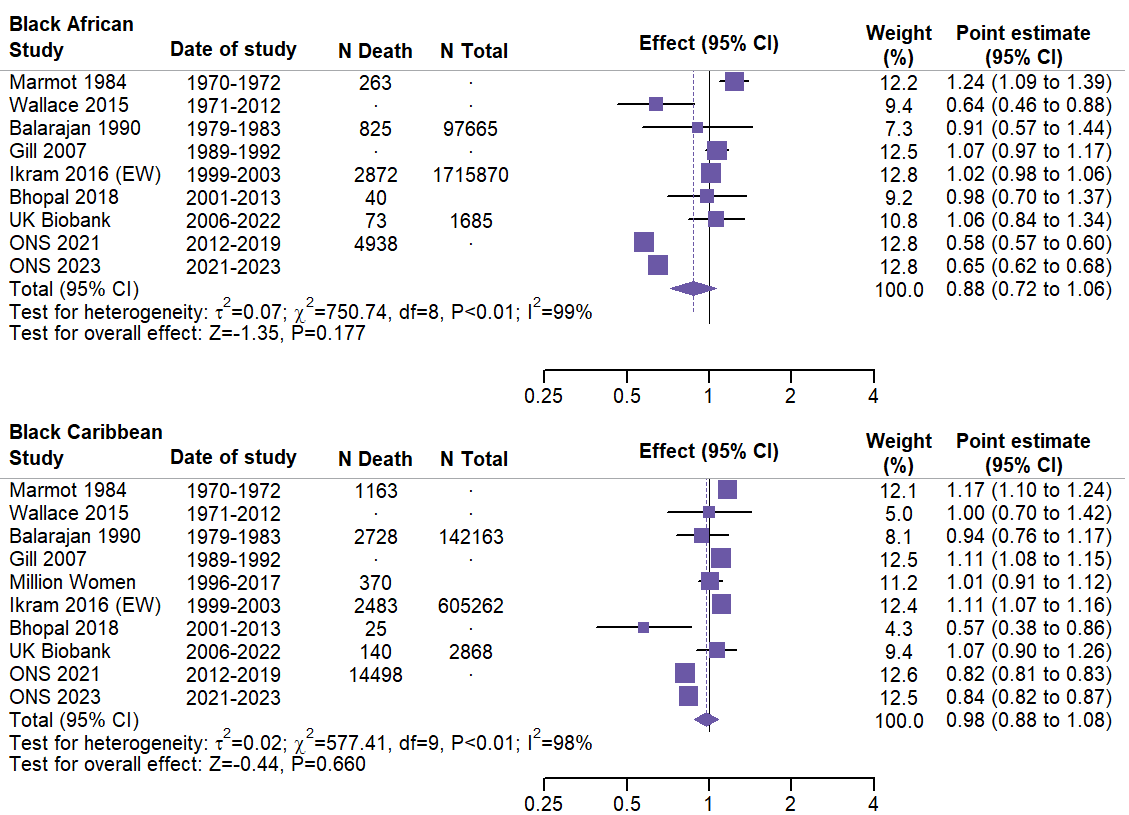


*The Million Women Study combined Black African and Black Caribbean + Other ethnicity together as Black + Other but the majority of participants in this category would be of Black Caribbean ethnicity.


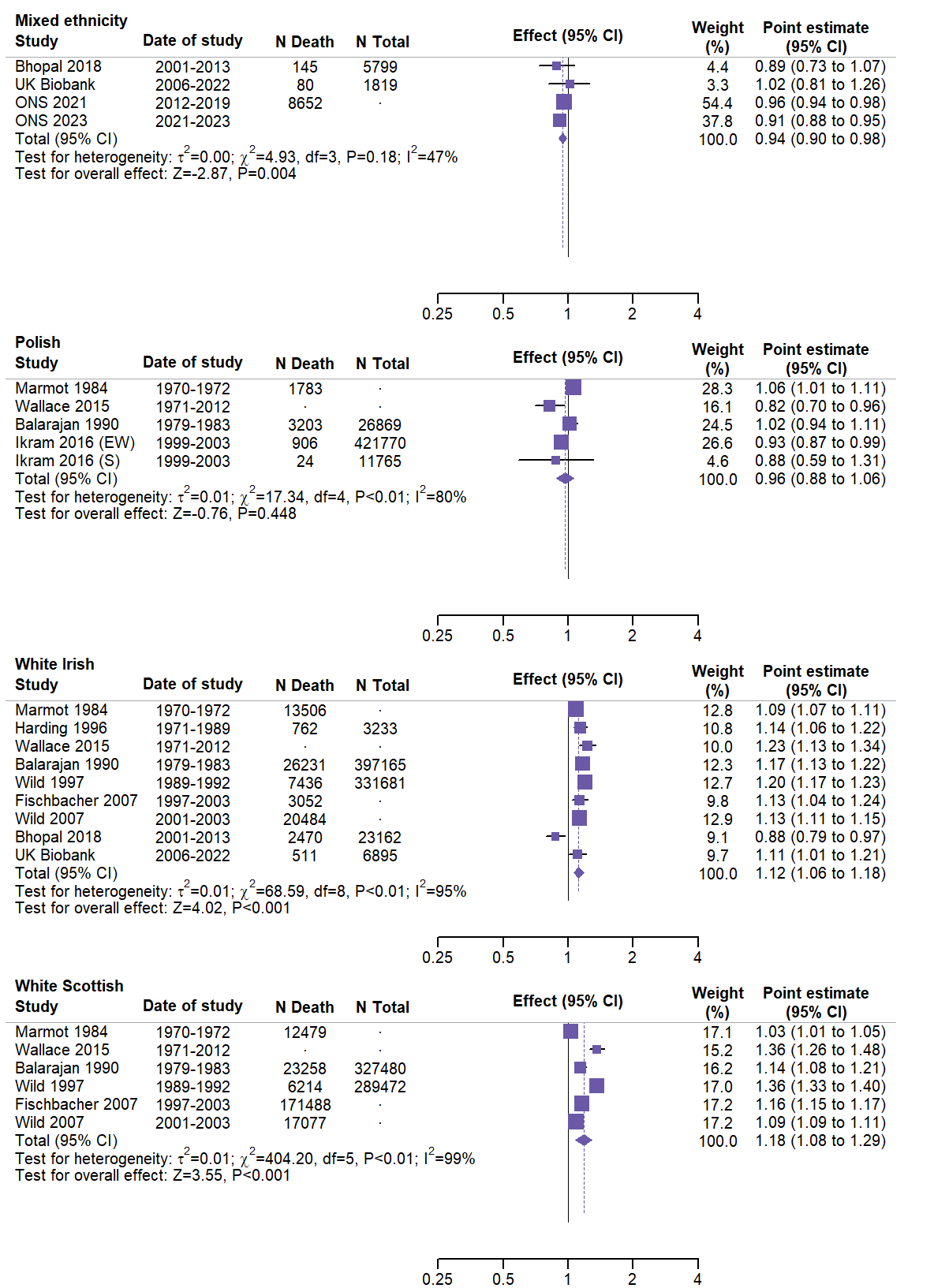


Note: All included studies had a White English/Welsh or total population comparator apart from the following studies with a White Scottish comparator: Bhopal 2018 and Ikram 2016 (S).

# Supplement 8: Age-adjusted all-cause mortality by ethnicity, stratified by country of birth


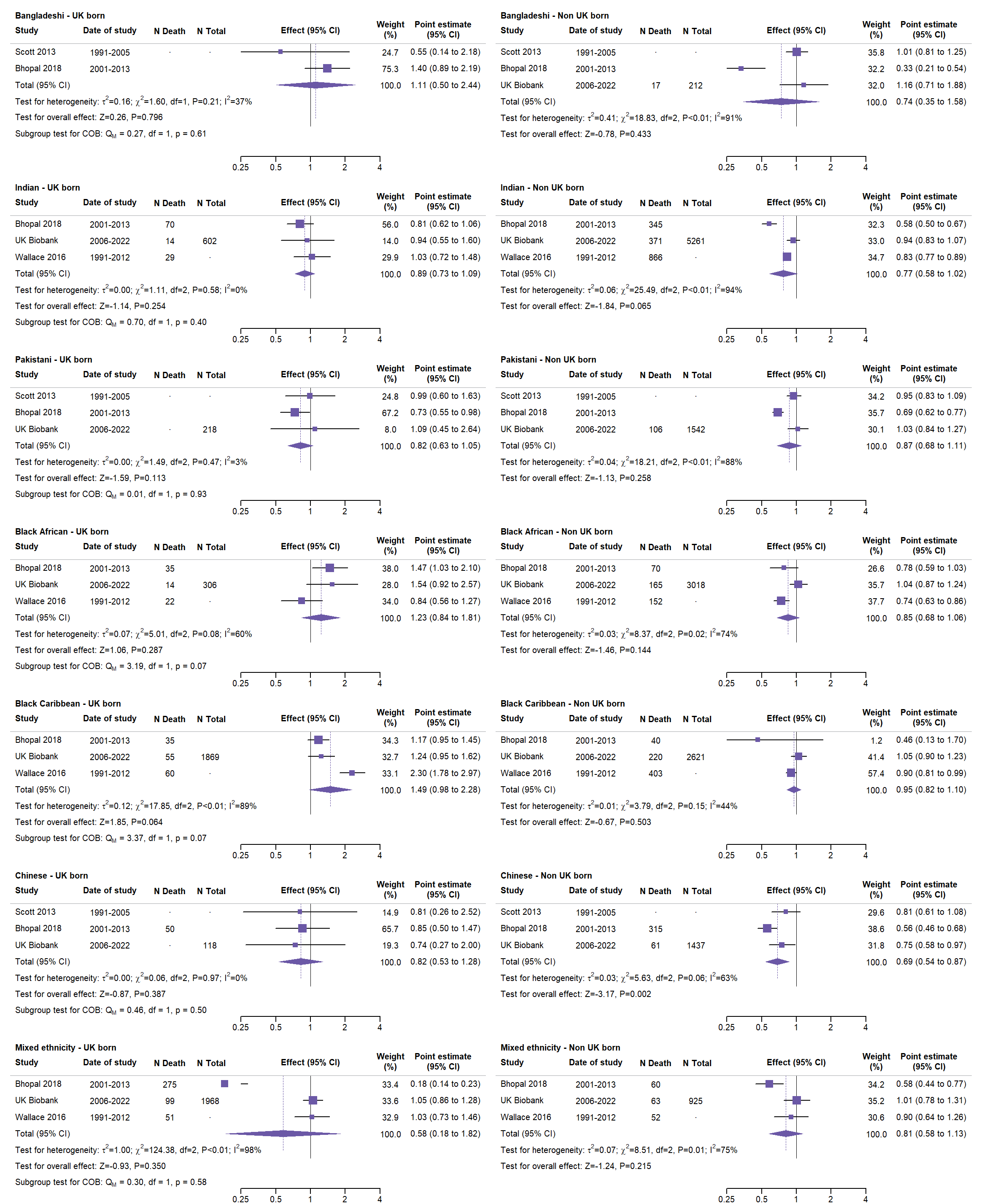

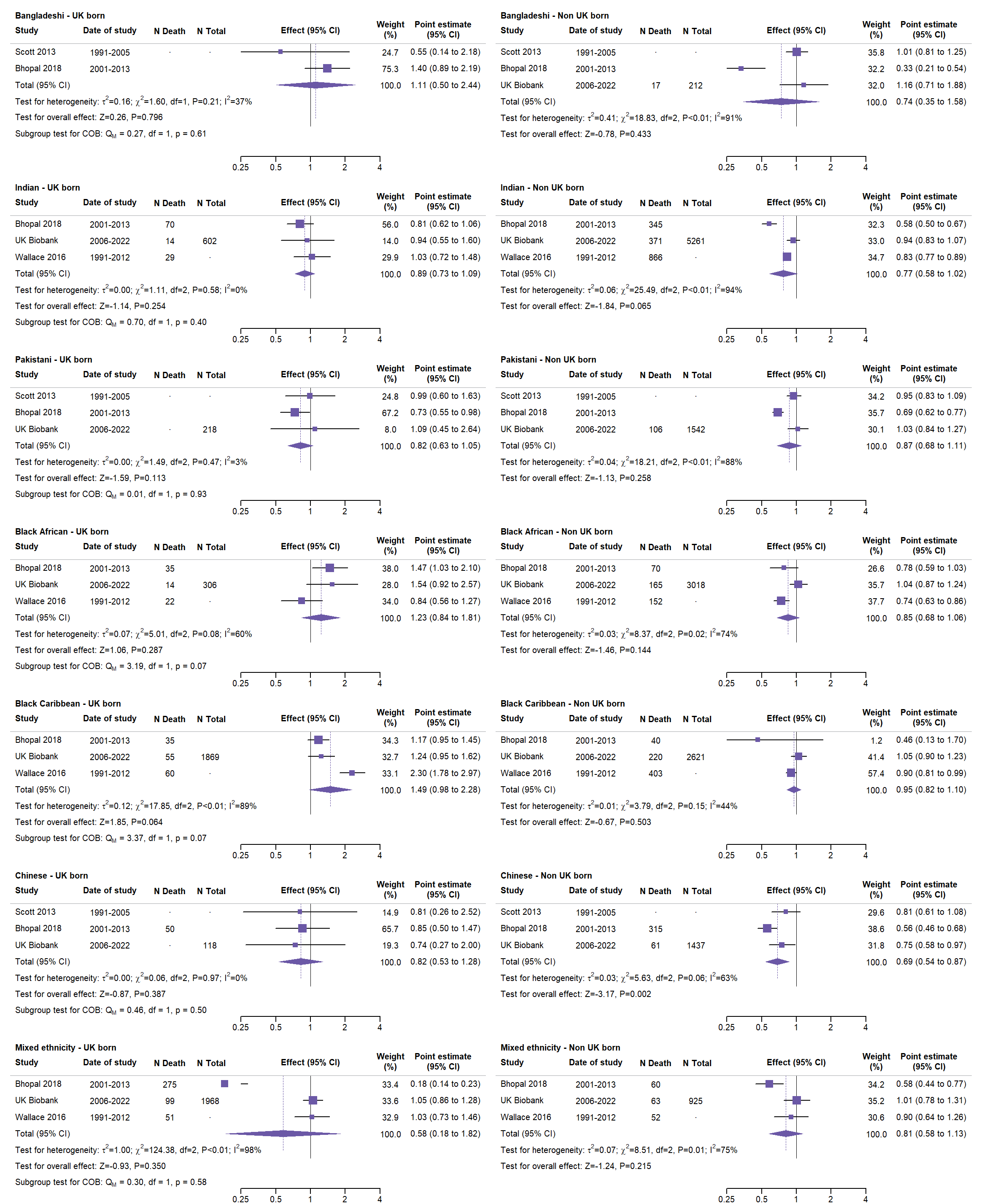

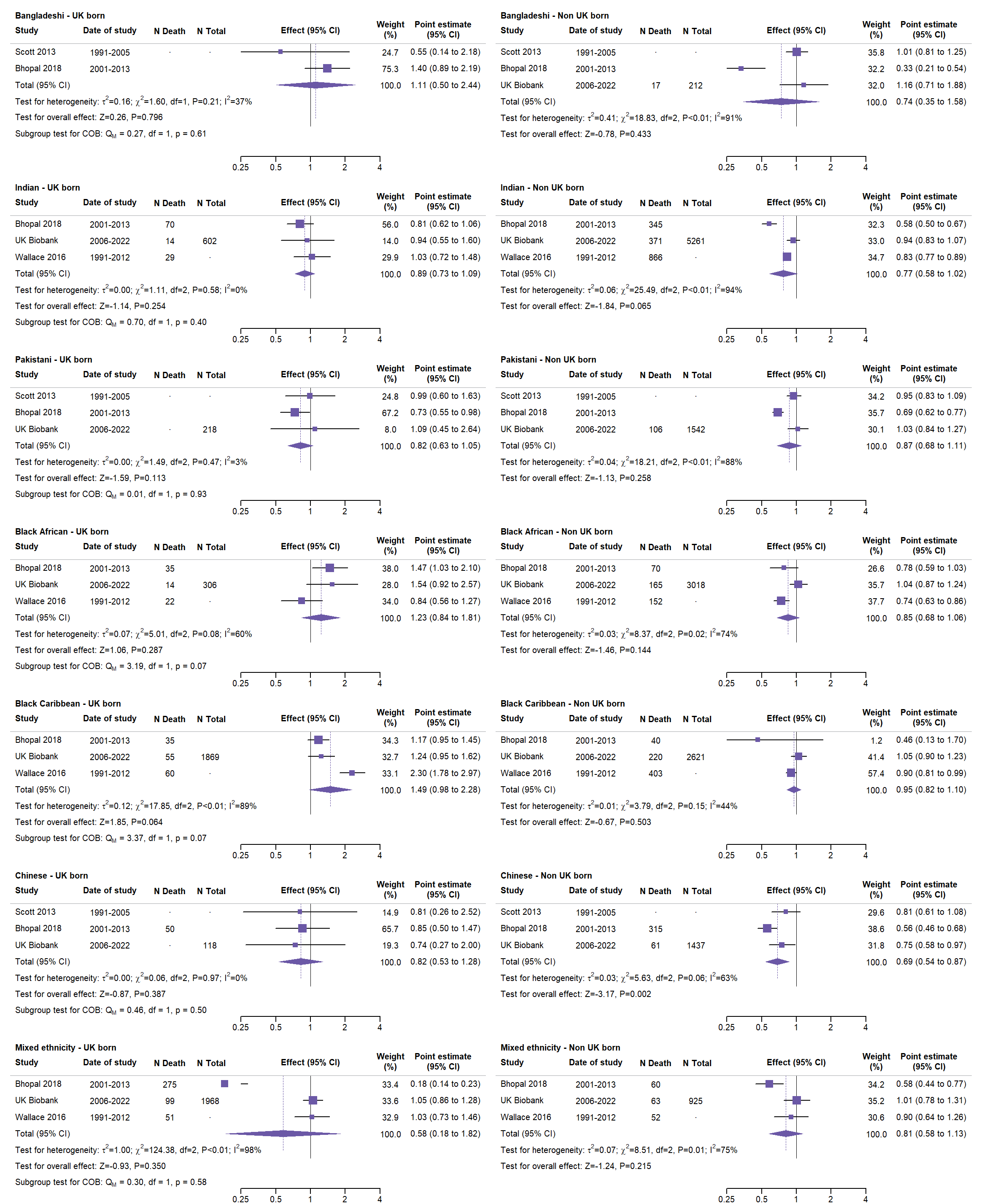

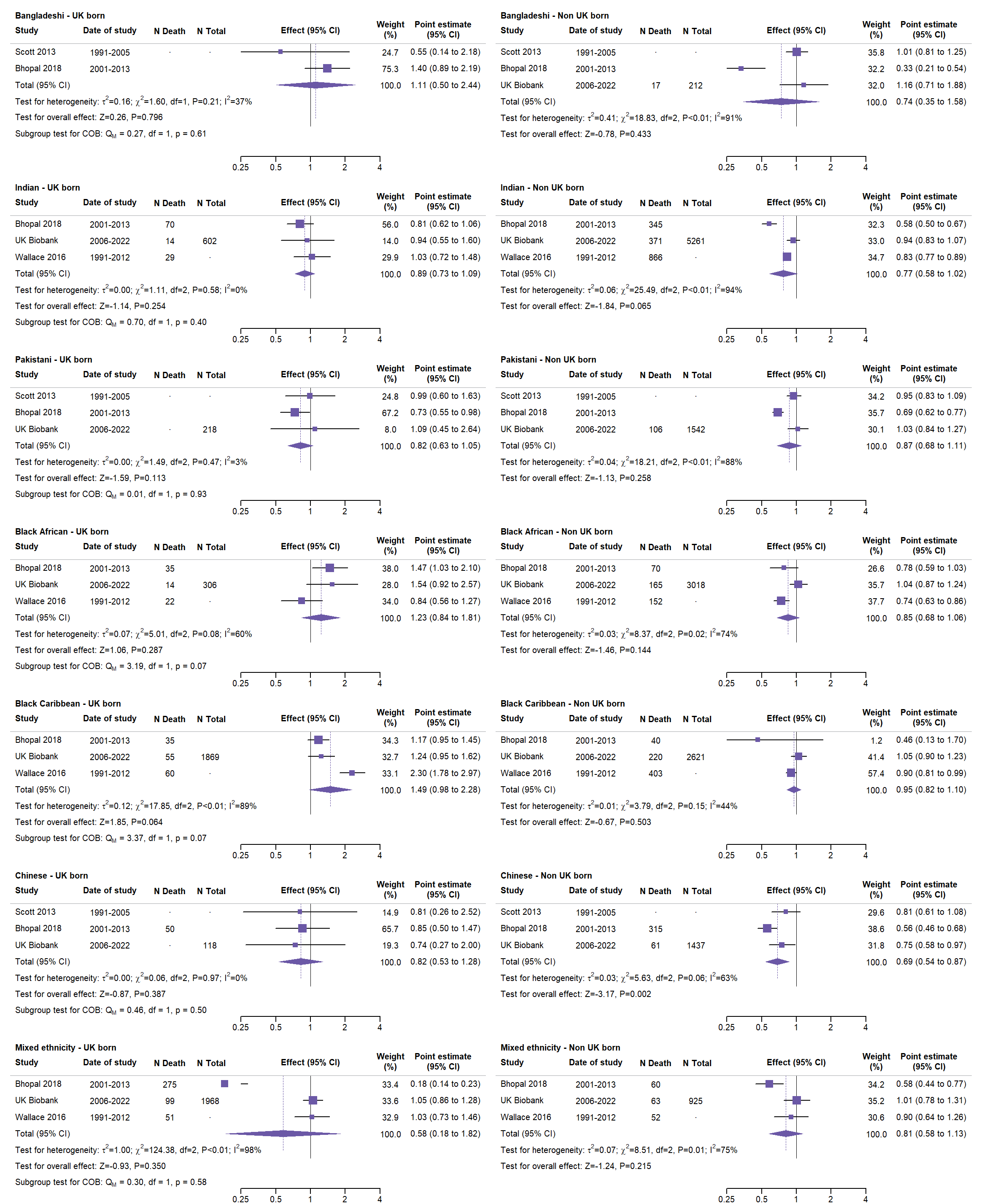


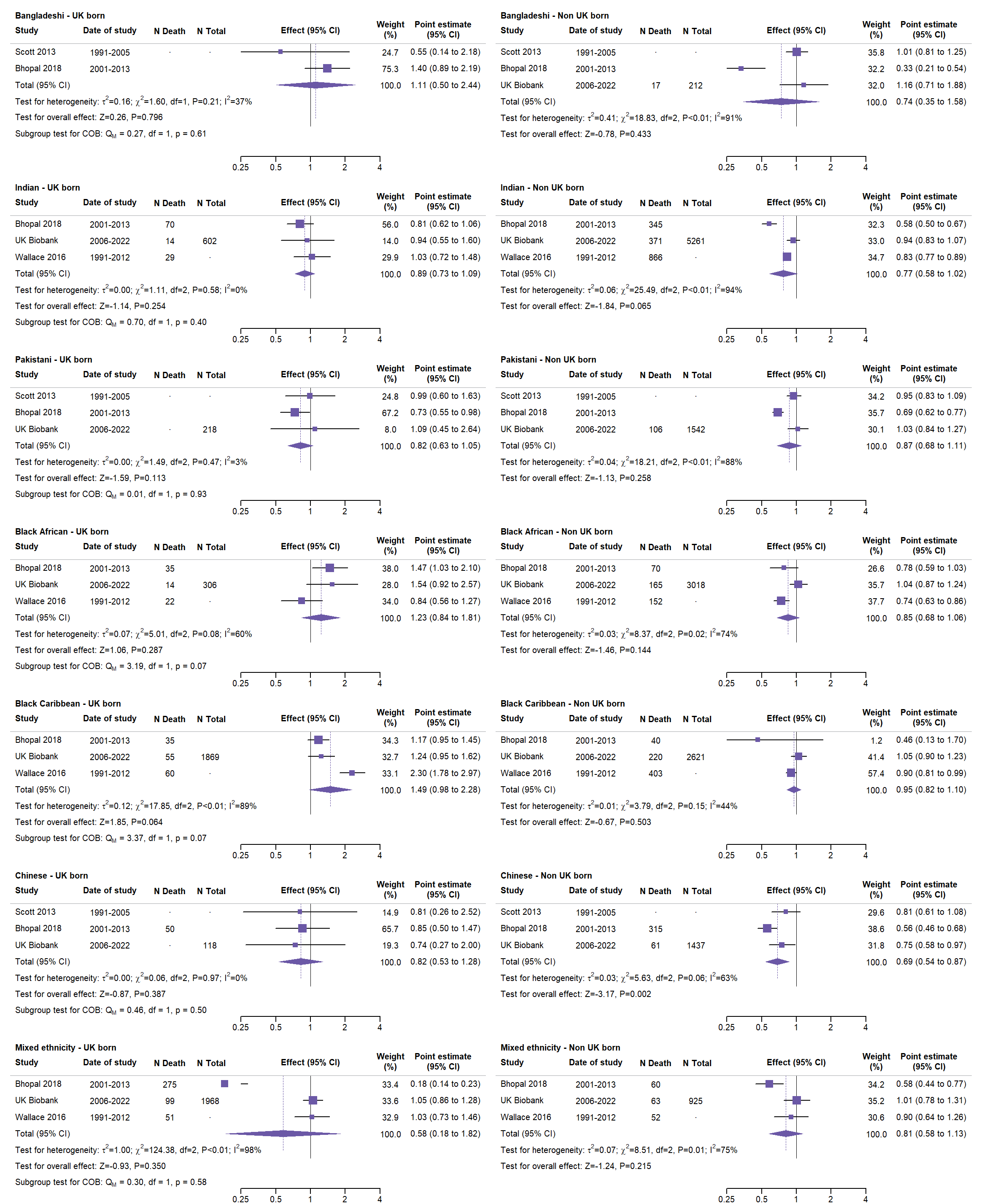

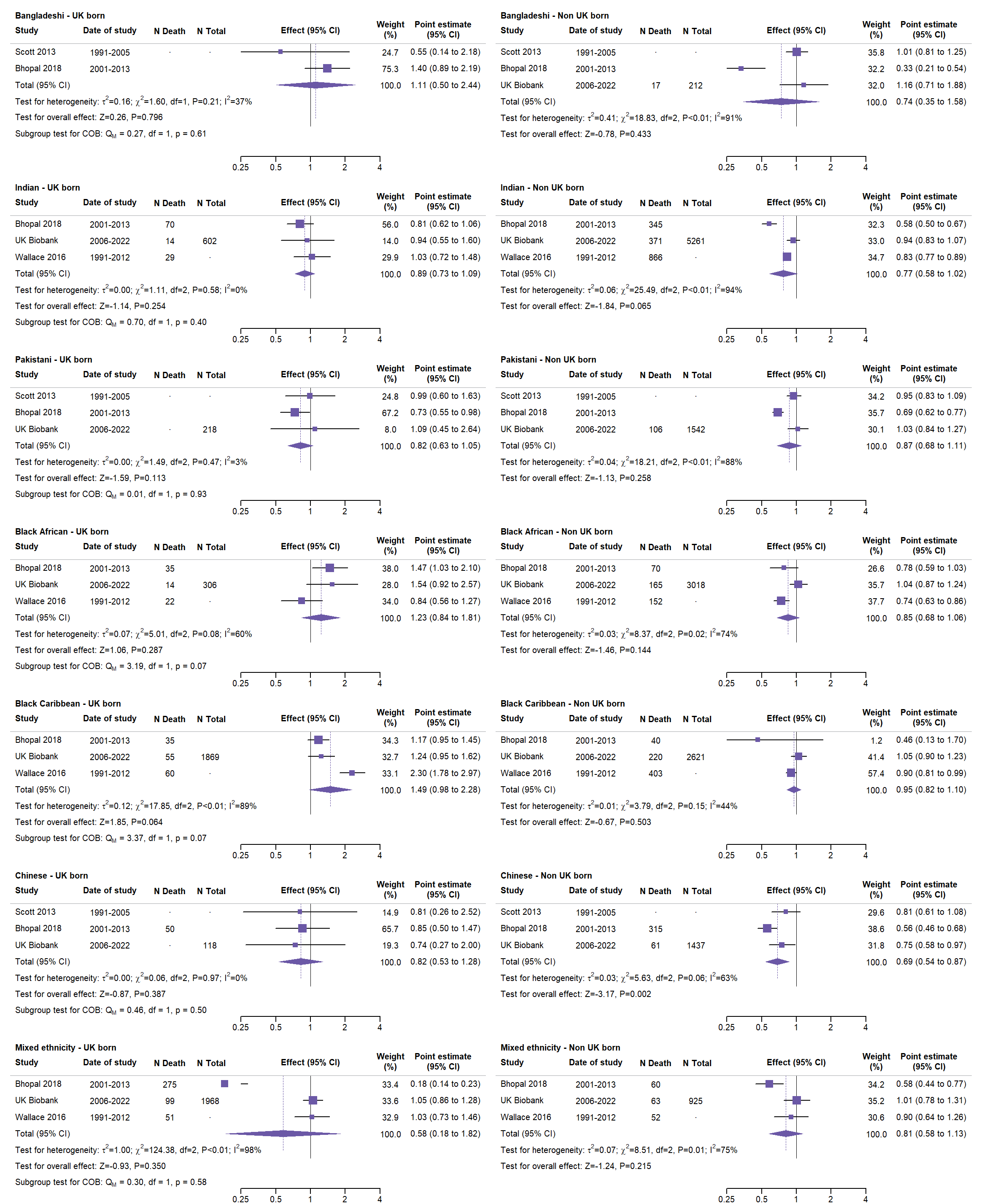

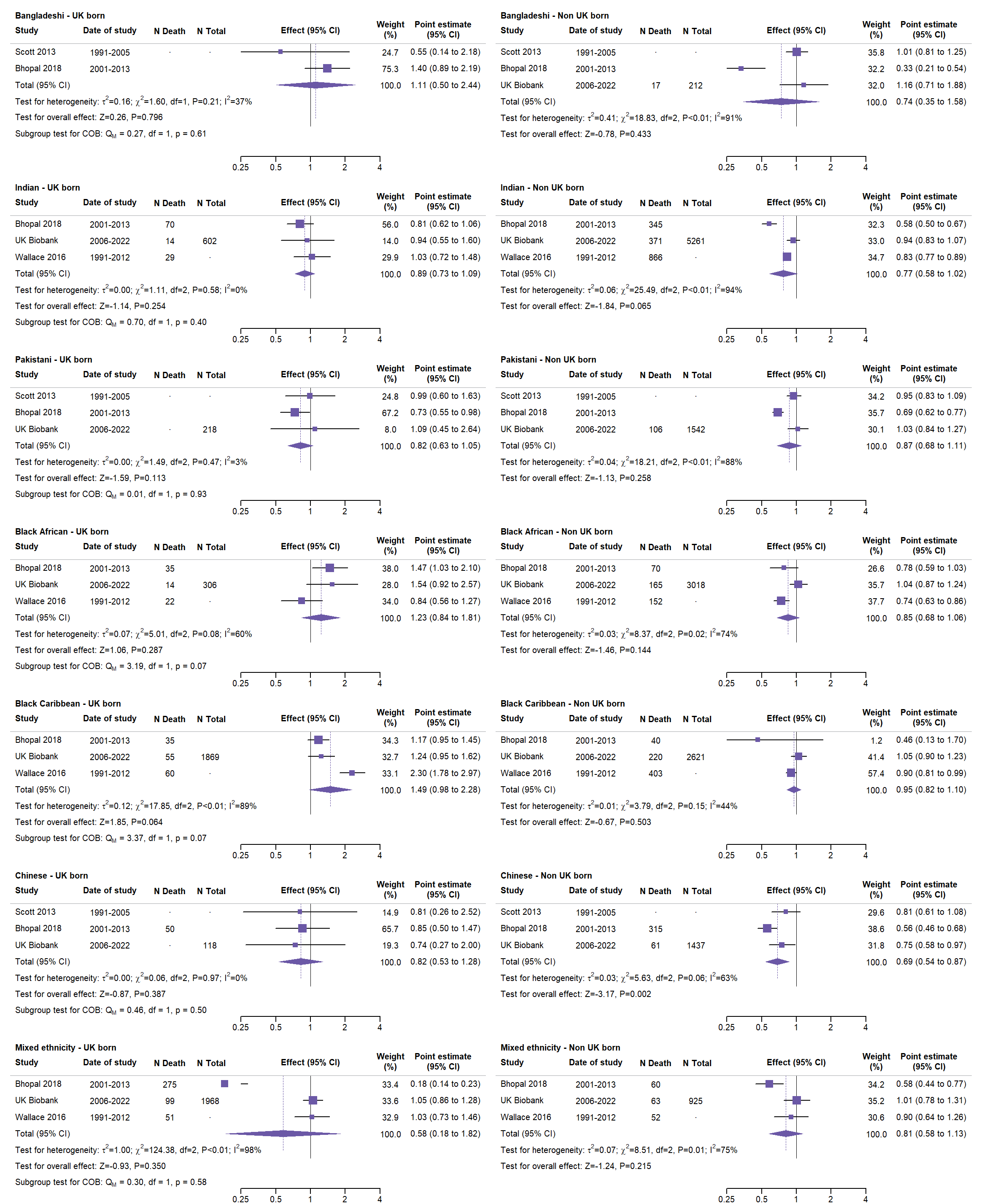

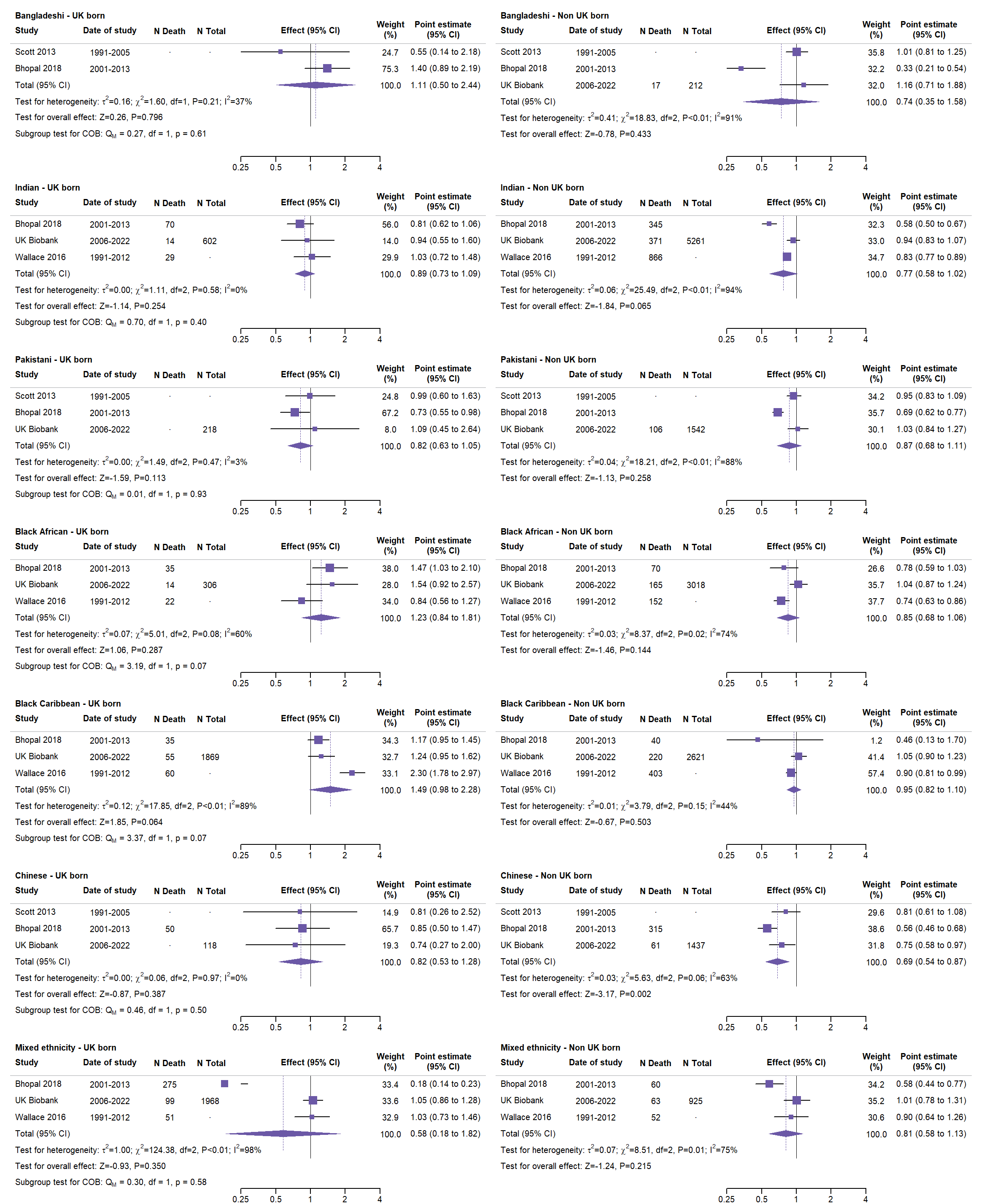


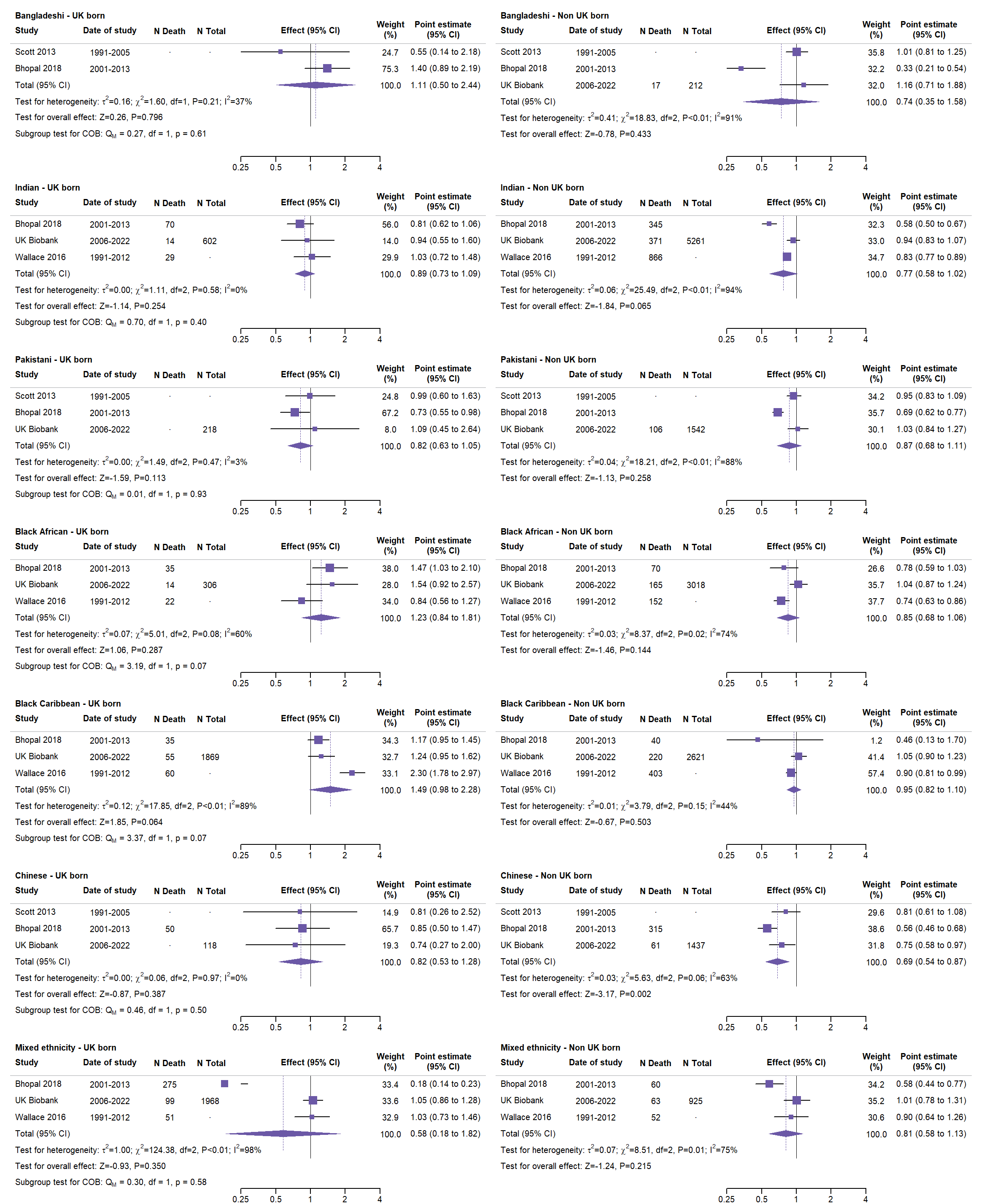


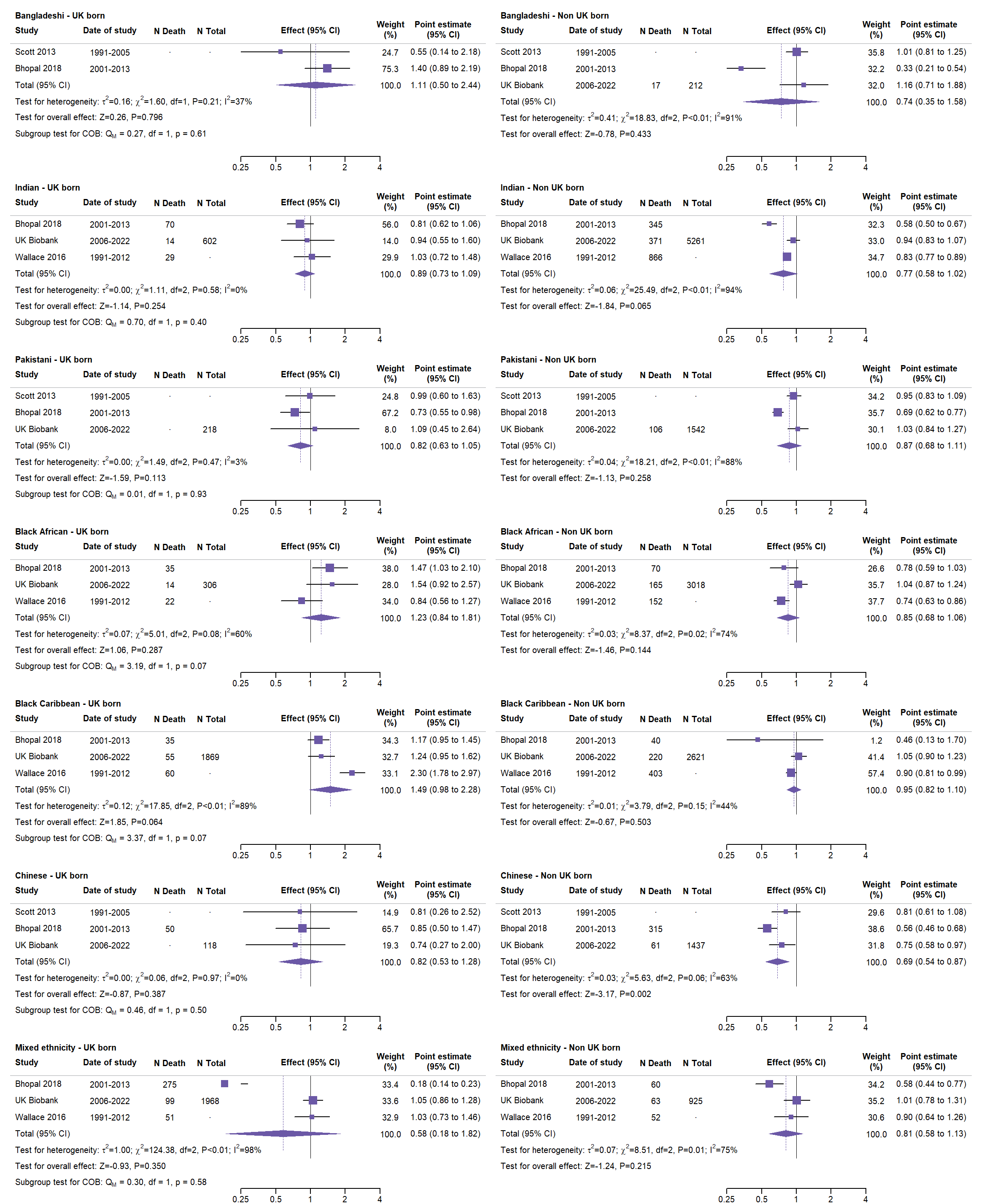

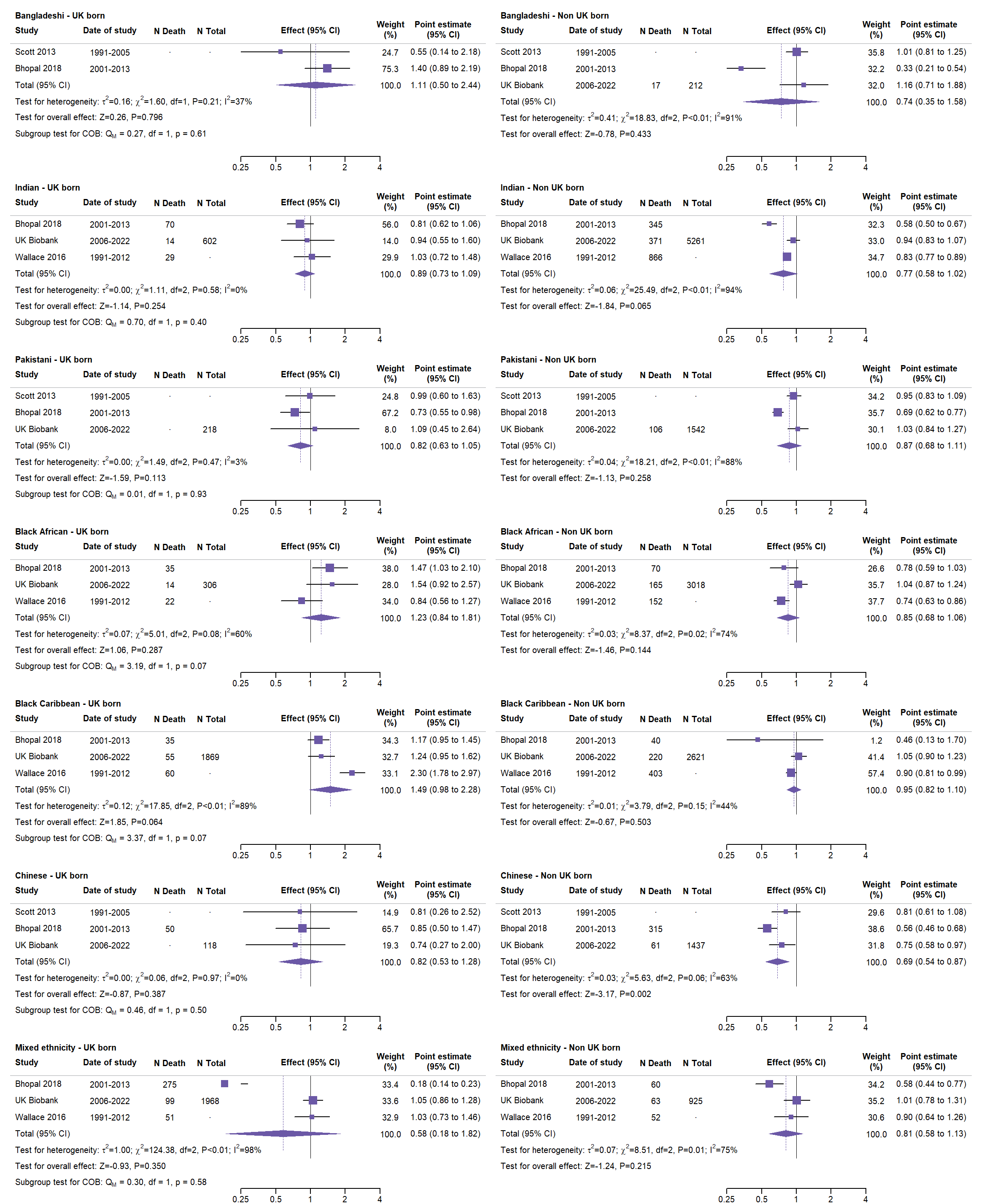


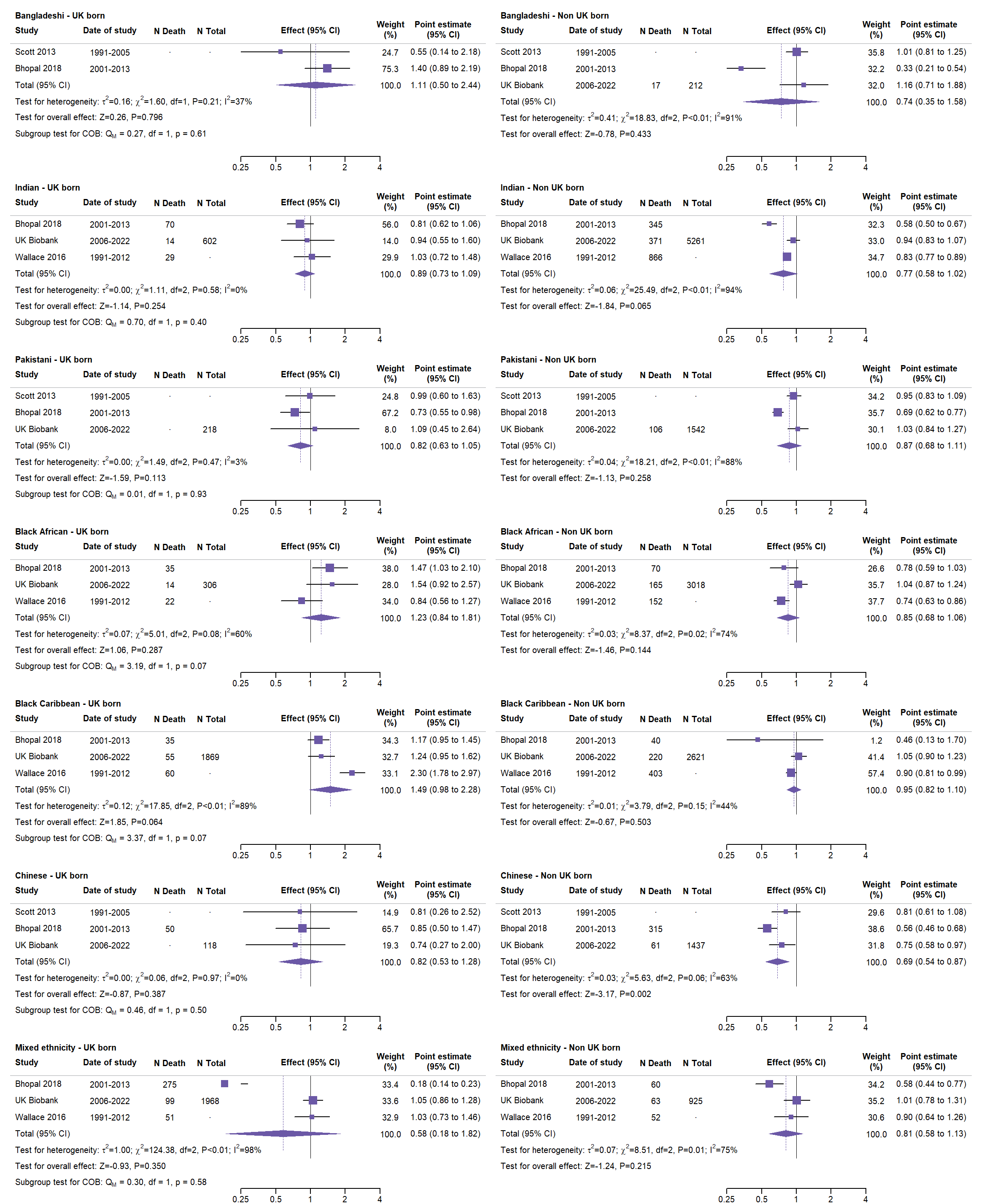


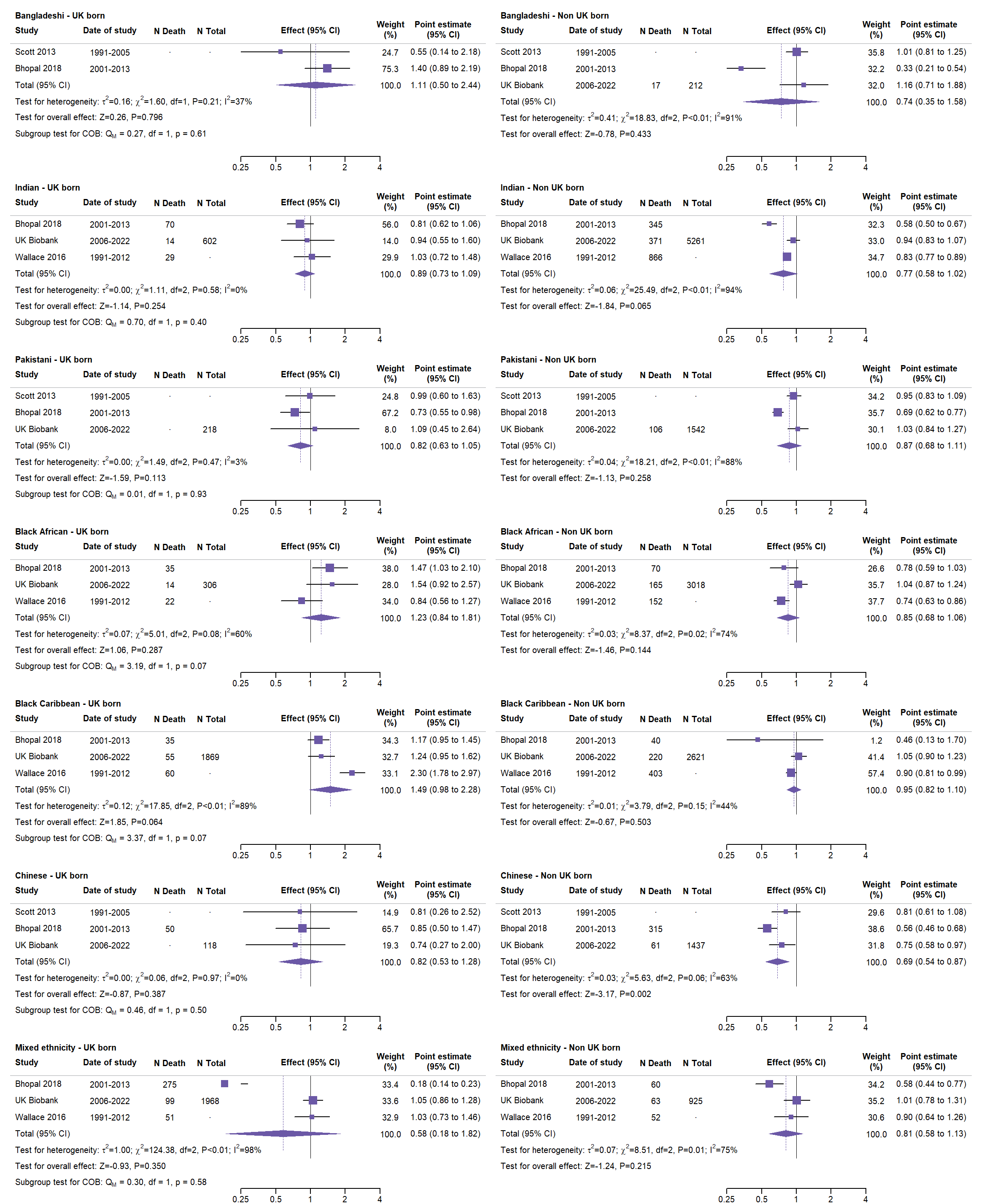


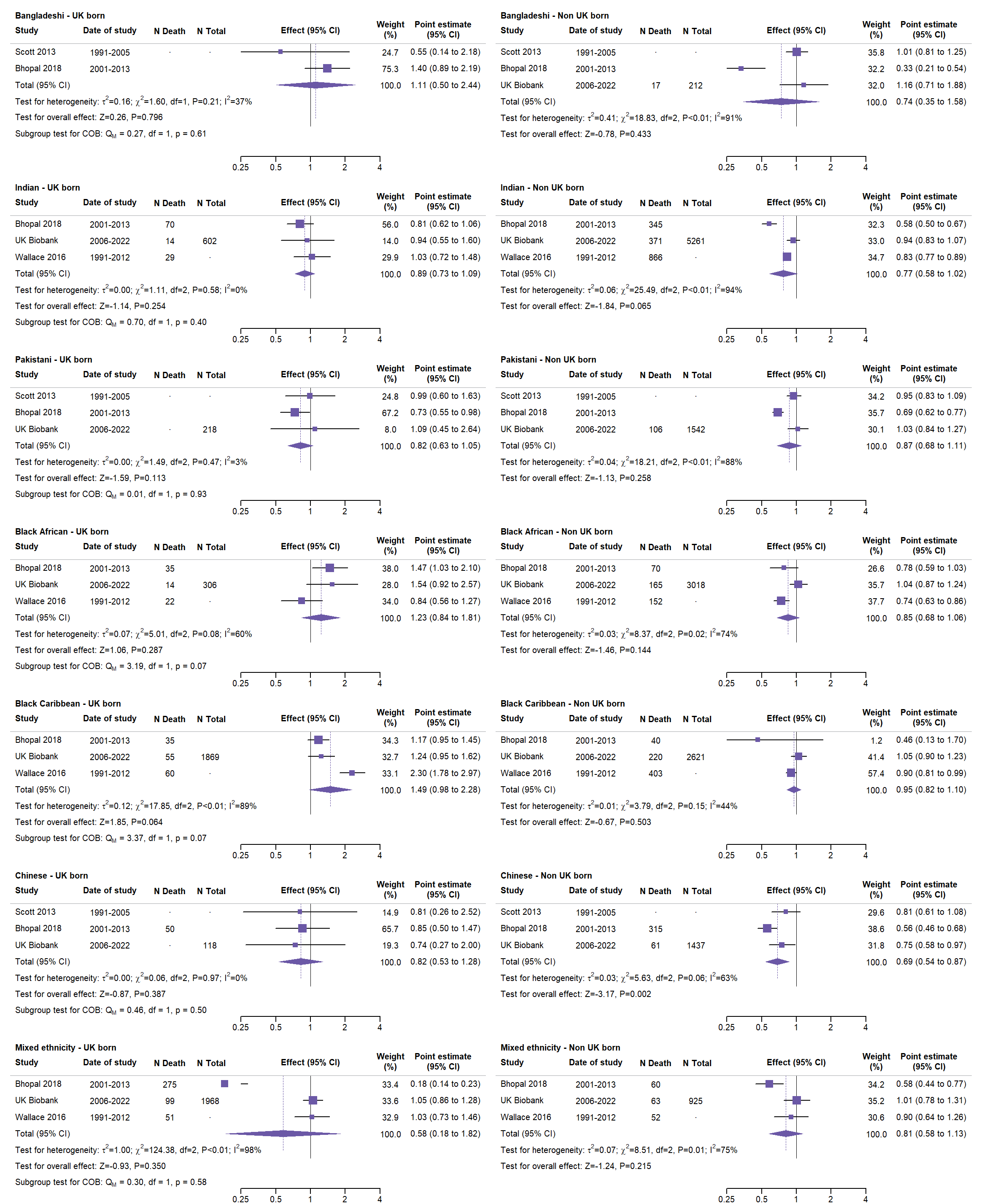


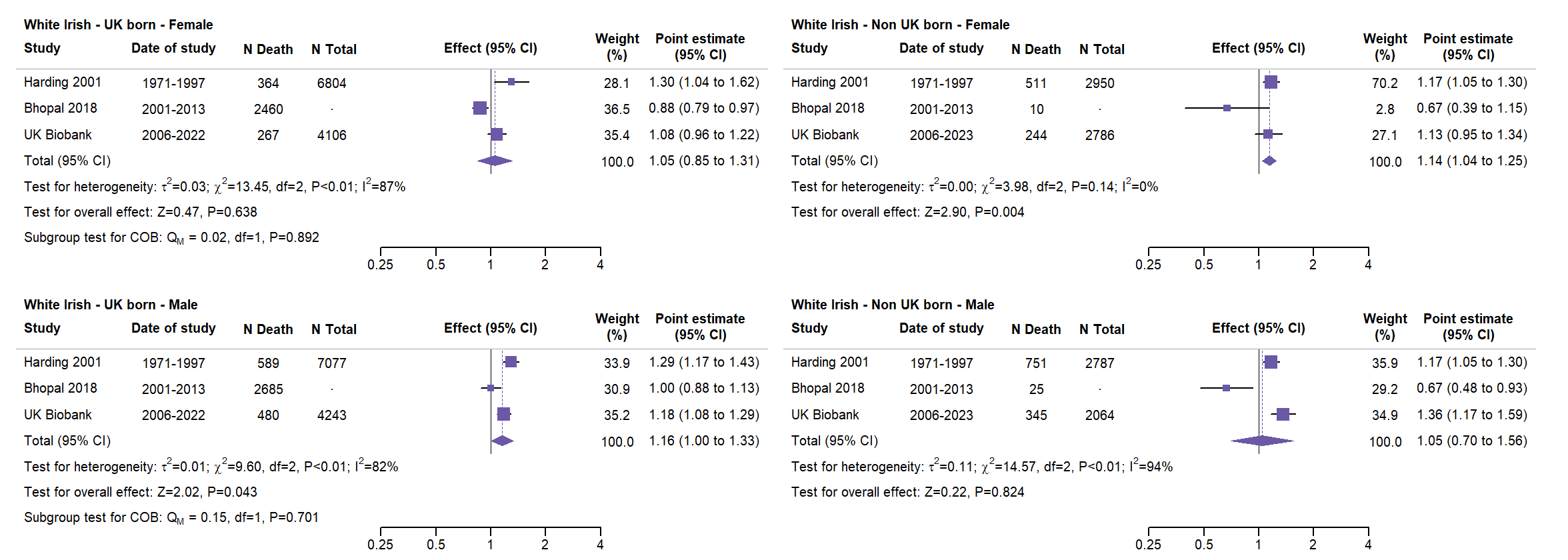

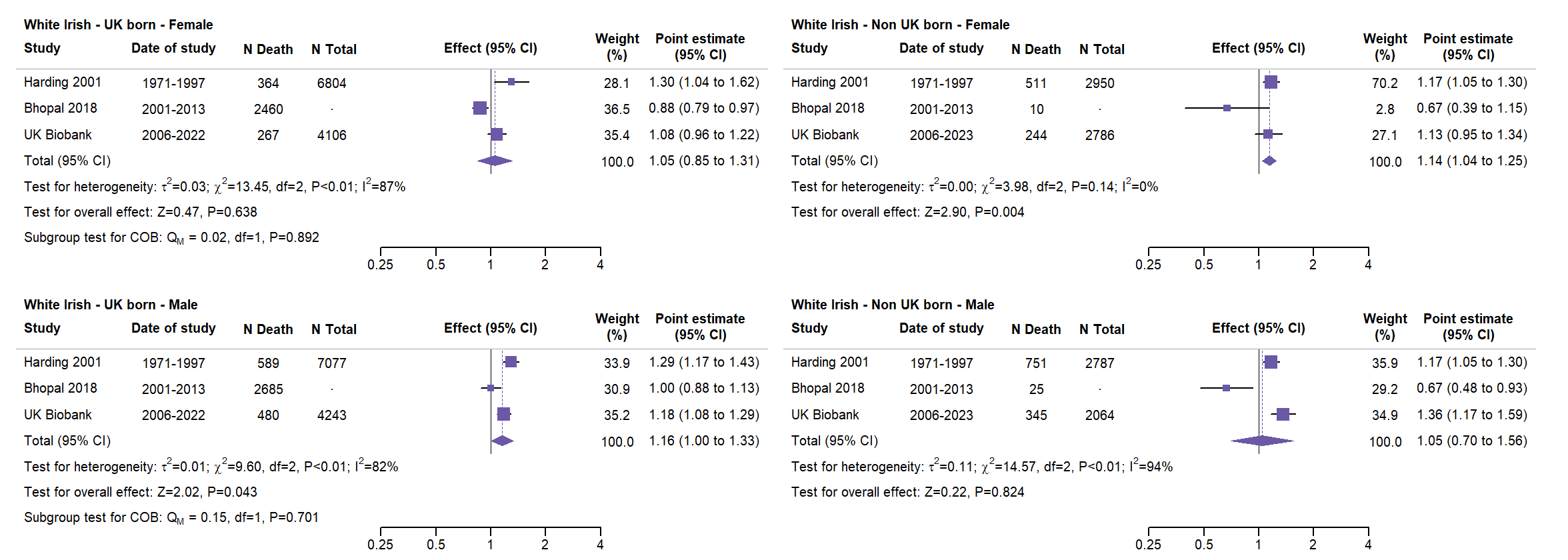

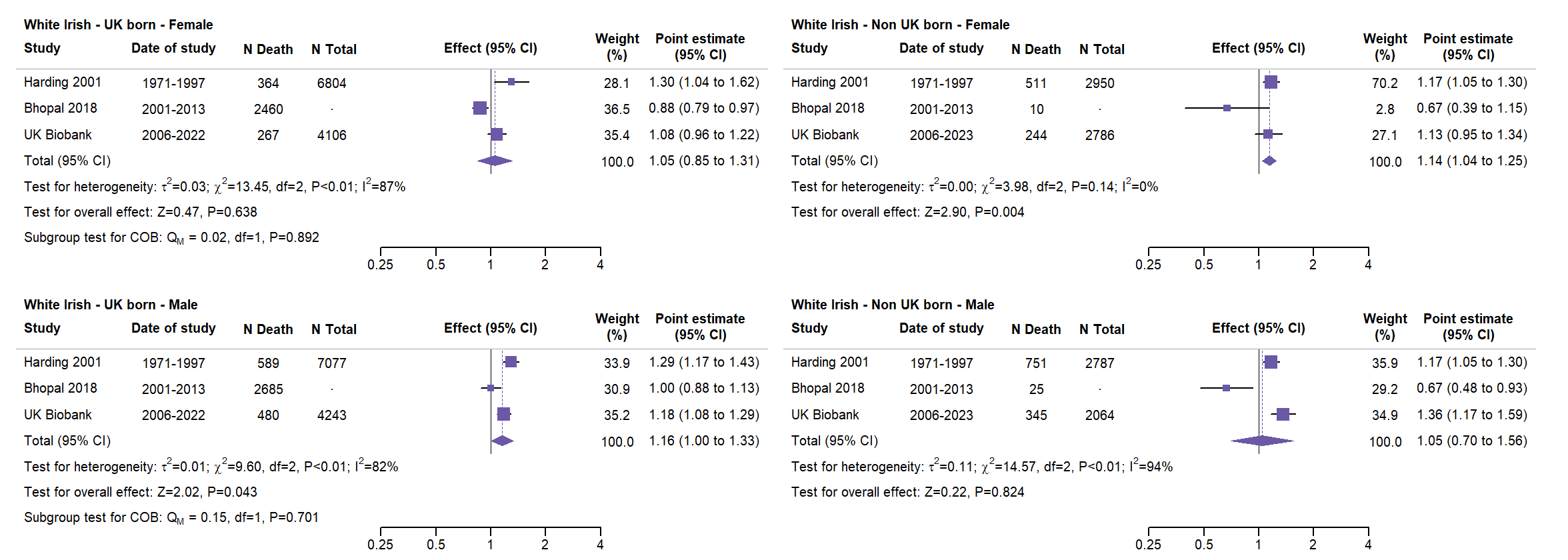
*Sex-stratified* *results*
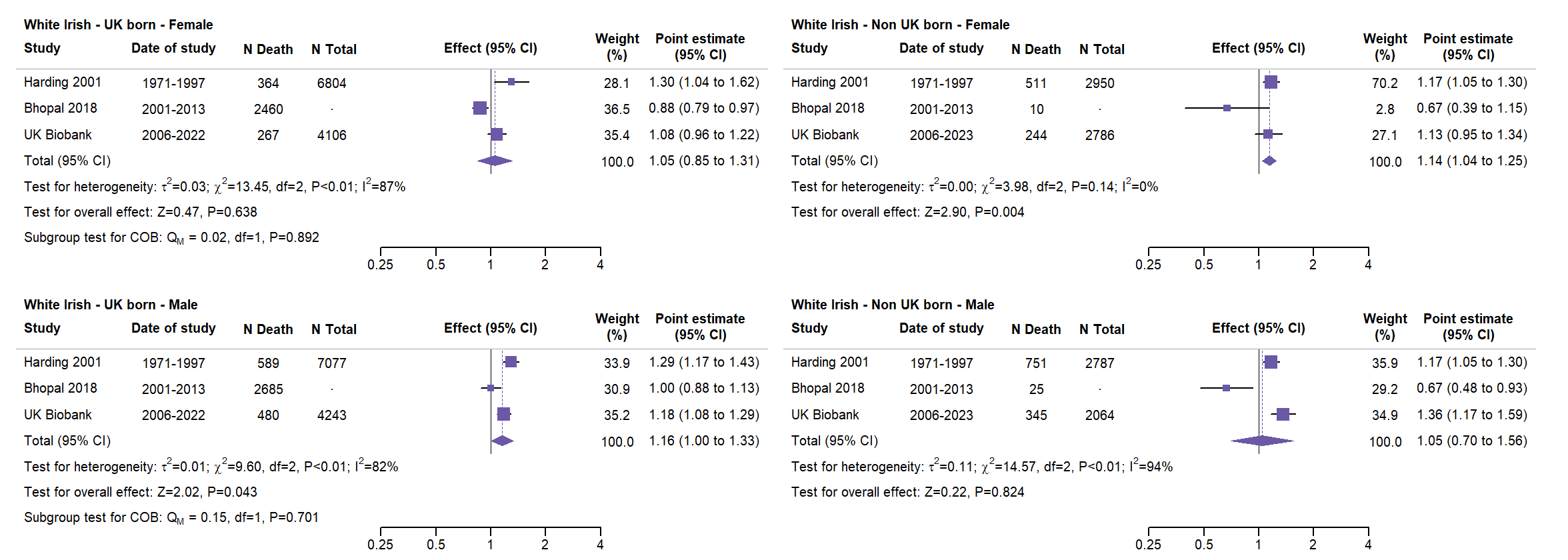


Note: All included studies had a White English/Welsh or total population comparator apart from the following studies with a White Scottish comparator: Bhopal 2018.

# Supplement 9: Age and SEP adjusted all-cause mortality by ethnicity

*In males*


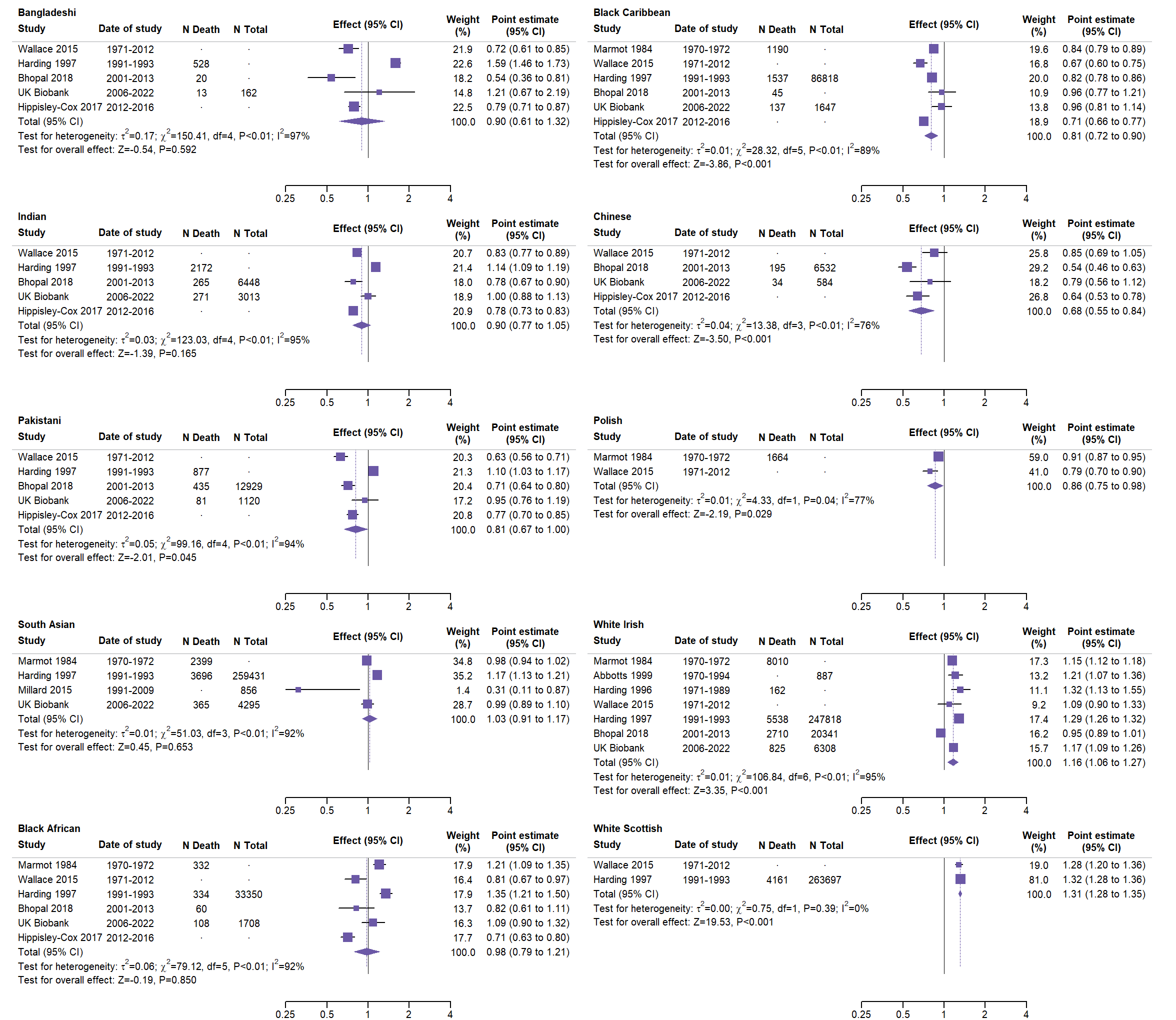


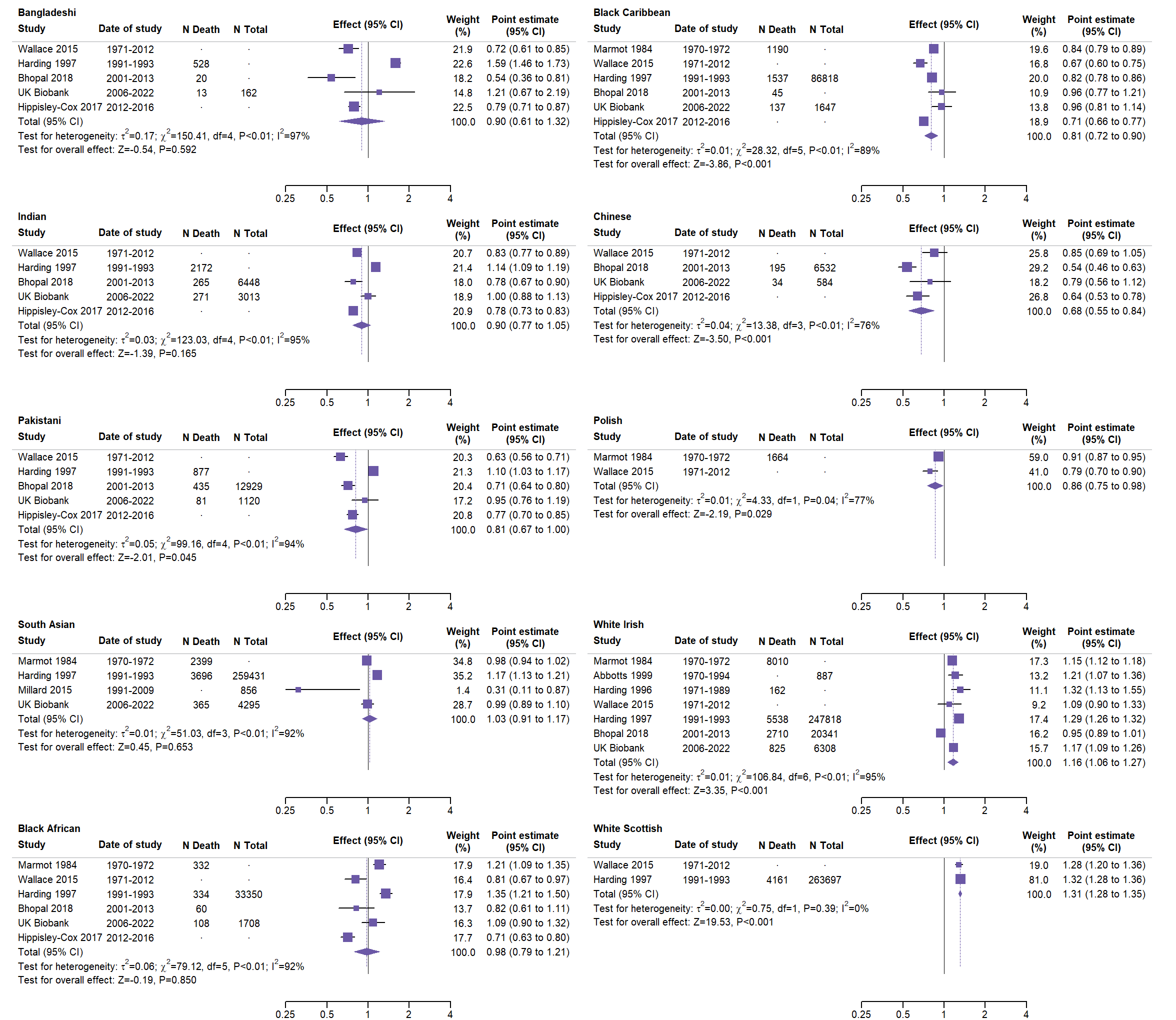

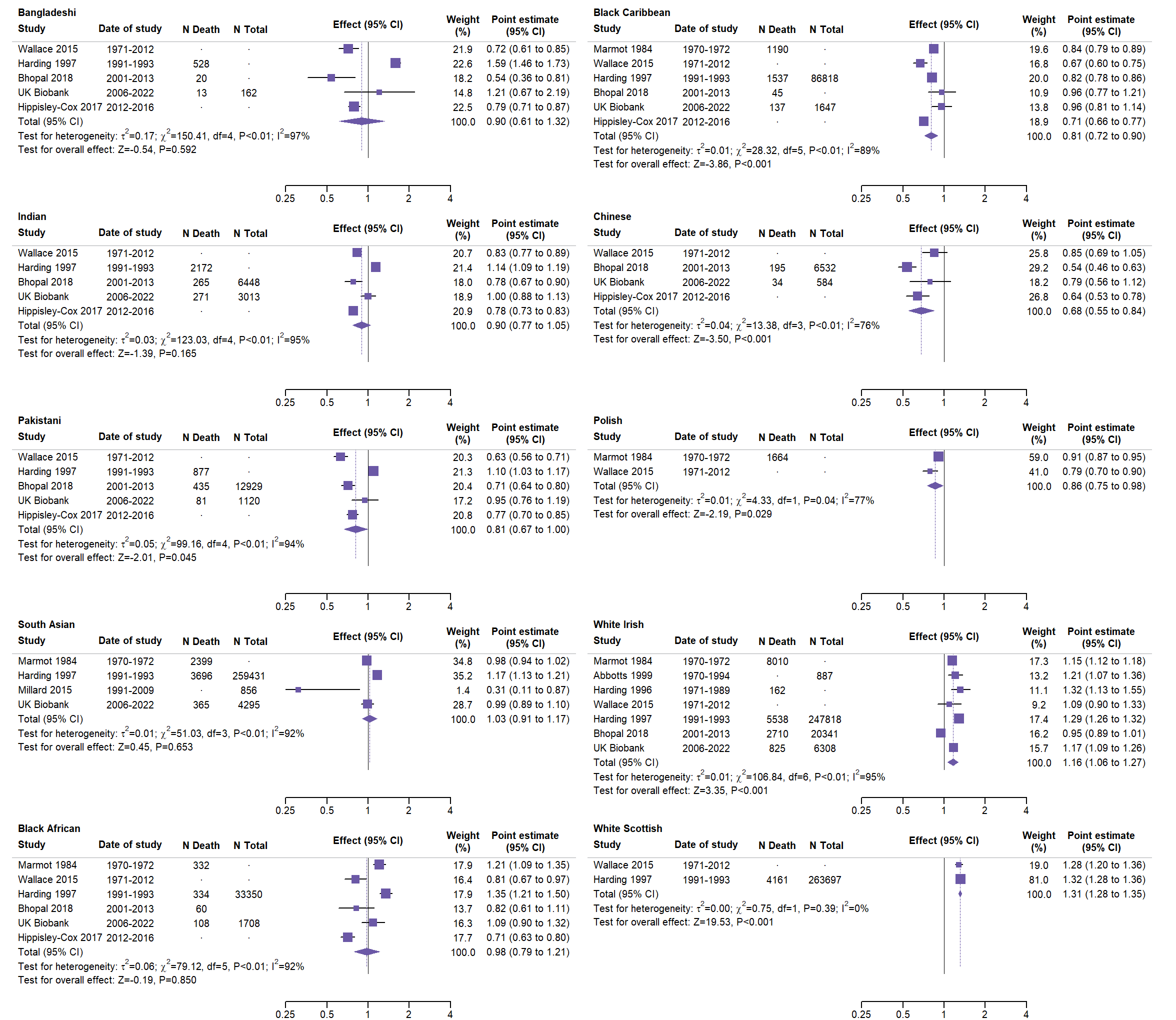


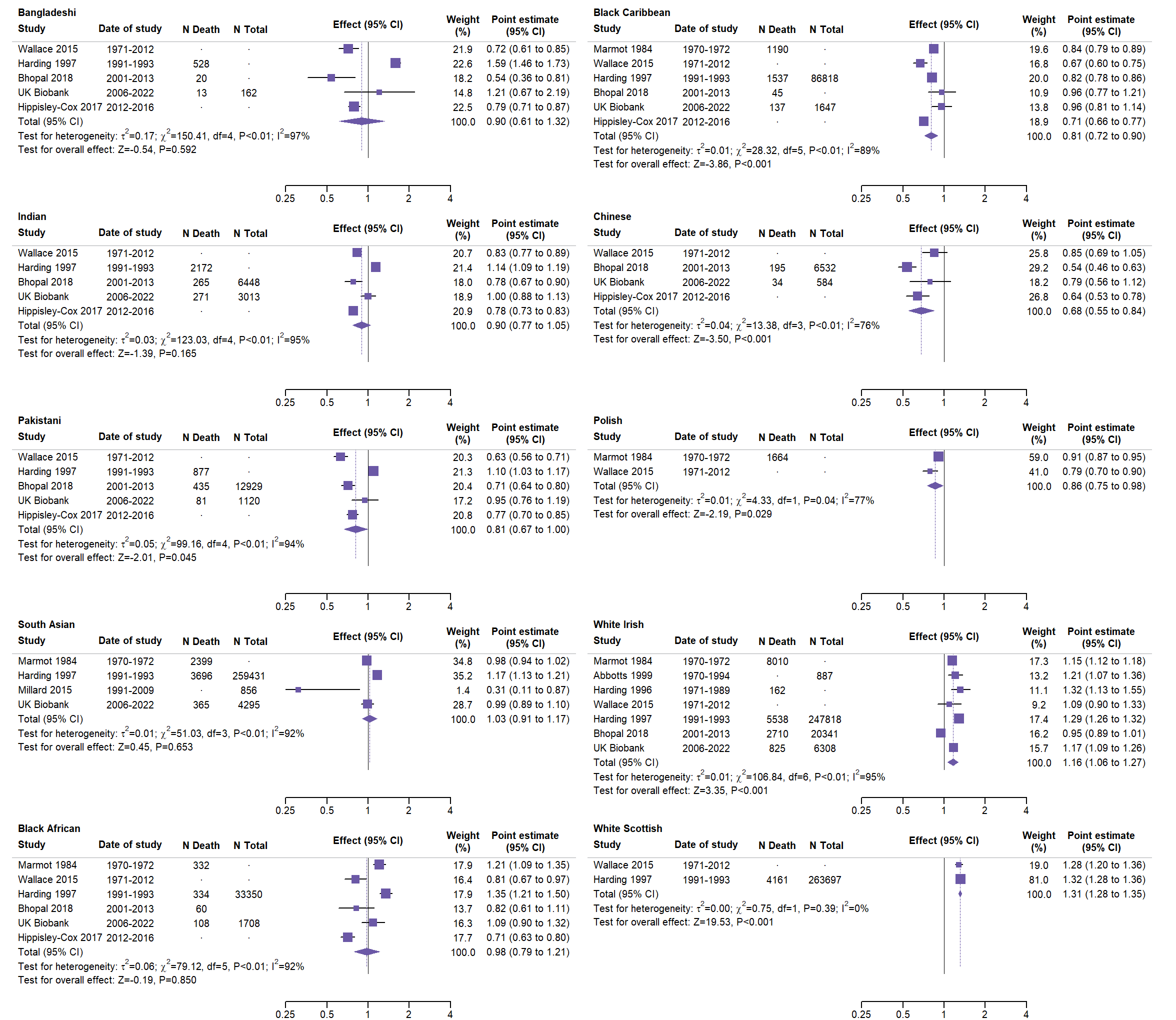


Note: All included studies had a White English/Welsh or total population comparator apart from the following studies with a White Scottish comparator: Bhopal 2018 and Abbotts 1999.


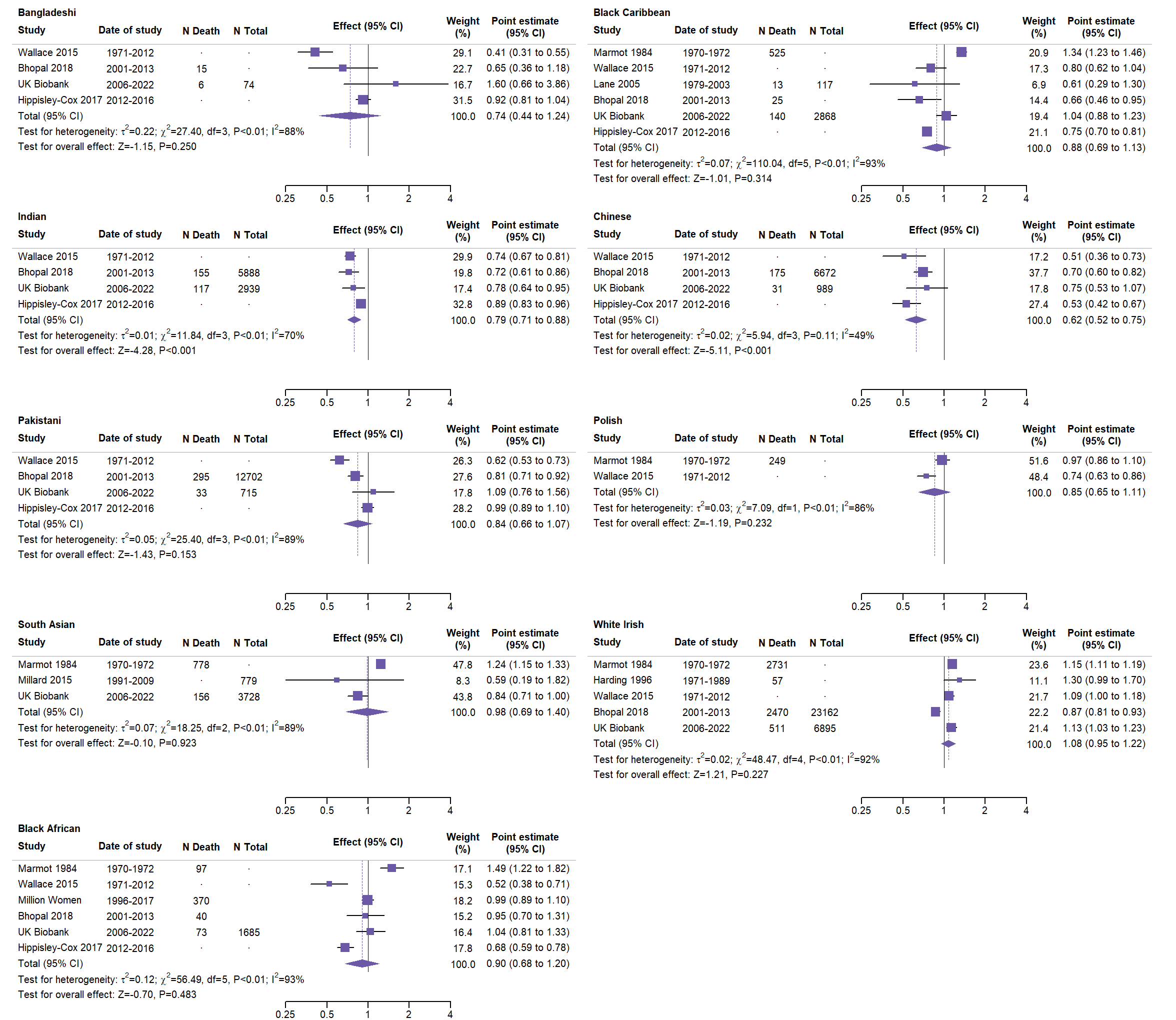
*In females*


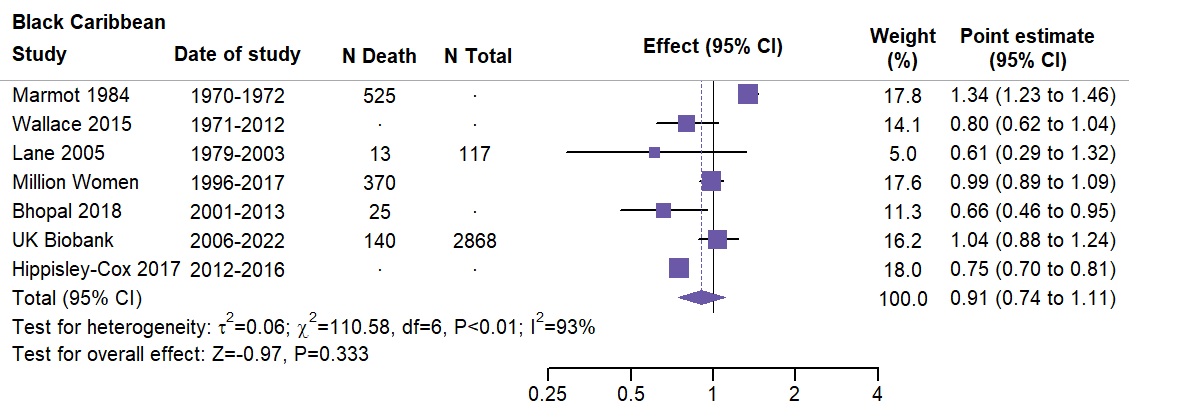

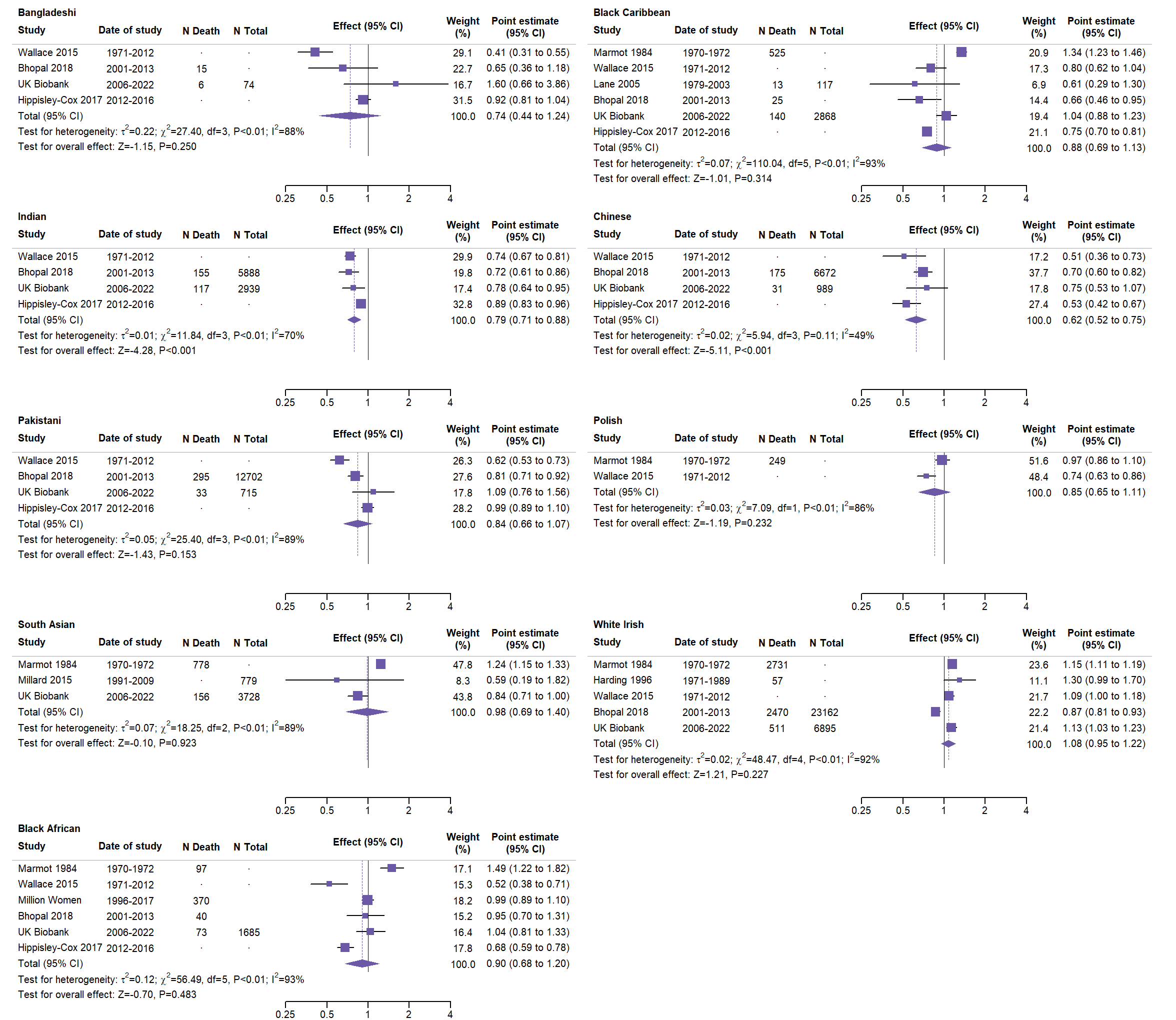

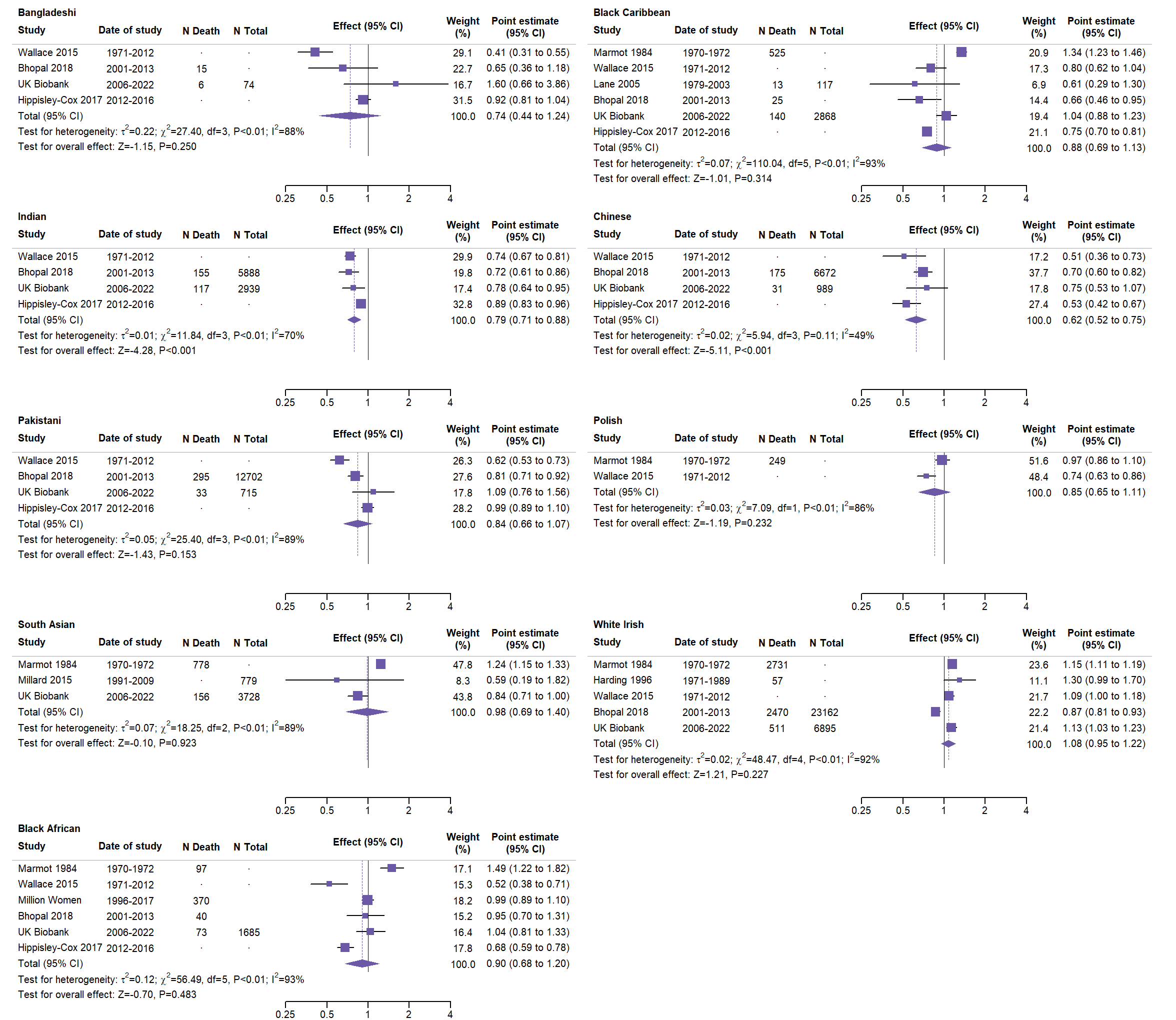


*

*The Million Women Study combined Black African and Black Caribbean + Other ethnicity together as Black + Other but the majority of participants in this category would be of Black Caribbean ethnicity.


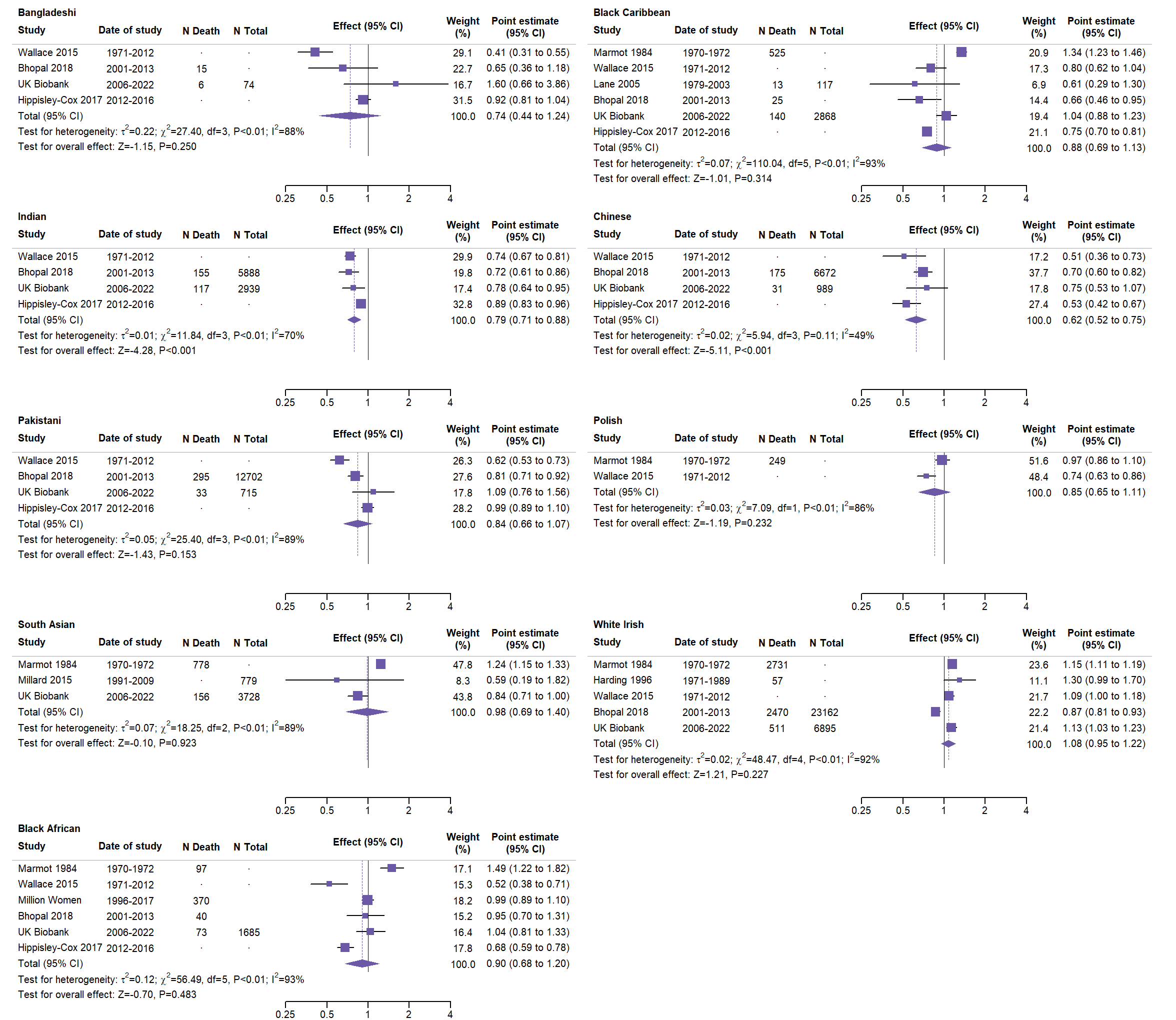


Note: All included studies had a White English/Welsh or total population comparator apart from the following studies with a White Scottish comparator: Bhopal 2018.

# Supplement 10: Table comparing age adjusted and age + SEP adjusted results

|  | **Age adjusted analyses** | | **Age and SEP adjusted analyses** | | |
| --- | --- | --- | --- | --- | --- |
| **Ethnic group** | Summary estimate | Confidence interval | | Summary estimate | Confidence interval |
|  |  |  | |  |  |
| **Males** |  |  | |  |  |
| Bangladeshi | 0.92 | 0.78-1.09 | | 0.90 | 0.61-1.32 |
| Indian | 0.88 | 0.78-0.98 | | 0.90 | 0.77-1.05 |
| Pakistani | 0.82 | 0.75-0.90 | | 0.81 | 0.67-1.00 |
| Black African | 0.93 | 0.79-1.08 | | 0.98 | 0.79-1.21 |
| Black Caribbean | 0.95 | 0.87-1.03 | | 0.81 | 0.72-0.90 |
| Chinese | 0.74 | 0.64-0.86 | | 0.68 | 0.55-0.84 |
| Mixed ethnicity | 0.99 | 0.90-1.09 | | - | - |
| Polish | 1.09 | 0.93-1.27 | | 0.86 | 0.75-0.98 |
| White Irish | 1.22 | 1.15-1.29 | | 1.16 | 1.06-1.27 |
| White Scottish | 1.19 | 1.11-1.27 | | 1.31 | 1.28-1.35 |
|  |  |  | |  |  |
| **Females** |  |  | |  |  |
| Bangladeshi | 0.83 | 0.73-0.94 | | 0.74 | 0.44-1.24 |
| Indian | 0.88 | 0.76-1.01 | | 0.79 | 0.71-0.88 |
| Pakistani | 0.88 | 0.81-0.96 | | 0.84 | 0.66-1.07 |
| Black African | 0.88 | 0.72-1.06 | | 0.90 | 0.68-1.20 |
| Black Caribbean | 0.98 | 0.88-1.08 | | 0.91 | 0.74-1.11 |
| Chinese | 0.76 | 0.67-0.86 | | 0.62 | 0.52-0.75 |
| Mixed ethnicity | 0.94 | 0.90-0.98 | | - | - |
| Polish | 0.96 | 0.88-1.06 | | 0.85 | 0.65-1.11 |
| White Irish | 1.12 | 1.06-1.18 | | 1.08 | 0.95-1.22 |
| White Scottish | 1.18 | 1.08-1.29 | | - | - |

# Supplement 11: Table of SEP measures used in included studies

| **Study citation** | **Socioeconomic position (SEP) adjustment** | | **Non-SEP adjustment factors (except age and sex)** |
| --- | --- | --- | --- |
| Abbotts 1999 | Social class of father | | Nil |
| Bhaskaran 2021 | Index of Multiple Deprivation | | Body mass index,  Comorbidities,  Geographical region,  Smoking status |
| Bhopal 2018 | Education,  Housing tenure,  Scottish Index of Multiple Deprivation | | Nil |
| Carey 2021 | Index of Multiple Deprivation | | Body mass index,  Geographical region,  Smoking status |
| Connolly 2011 | Car availability  Education,  Housing tenure,  National Statistics Socio-Economic Classification,  Northern Ireland Index of Multiple Deprivation – income domain (2005) | | Marital status |
| Harding 1996 | Social class | | Nil |
| Harding 1997 | Social class | | Nil |
| Hayes 2017 | Social class | | Alcohol intake,  Body mass index,  Diabetes,  Diet,  Hypertension,  Low density lipoprotein,  Physical activity,  Smoking status,  Waist-to-hip ratio |
| Hippisley-Cox 2017 | Townsend scale | | Alcohol intake,  Biochemistry,  Body mass index,  Comorbidities,  Prescribed drugs,  Smoking status,  Unplanned hospital admissions or GP visits with specific symptoms (past twelve months) |
| Jesky 2013 | Index of Multiple Deprivation (2007) | | Comorbidities,  Renal function (albumin-creatinine ratio, estimated glomerular filtration rate),  Smoking status |
| Lane 2005 | Nil | | Blood pressure,  Body mass index,  Smoking status |
| Marmot 1984 | Social class | | Nil |
| Millard 2015 | Carstairs Deprivation Index (1991) | | Nil |
| ONS 2023 | Area deprivation quintile | | Nil |
| Stafford 2022 | Index of Multiple Deprivation (2015) | | Number of long-term medical conditions |
| Wallace 2014 | Social class,  Education | | Exit uncertainty |
| Wallace 2015 | Education,  Occupation type | | Cause of death,  Geographical region,  Marital status |
| Wright 2016 | Index of Multiple Deprivation (2010) | | Nil |
| *Unpublished datasets* | | | |
| UK Biobank | Education | | Nil |
| EPIC Norfolk | Education | | Nil |
| Million Women Study | Education | | Nil |
|  |  |  | |

# Supplement 12: Sex adjusted analyses

This supplementary file contains;

**S12a**: Age and sex-adjusted all-cause mortality by ethnicity

**S12b**: Age, sex and SEP-adjusted all-cause mortality by ethnicity

**
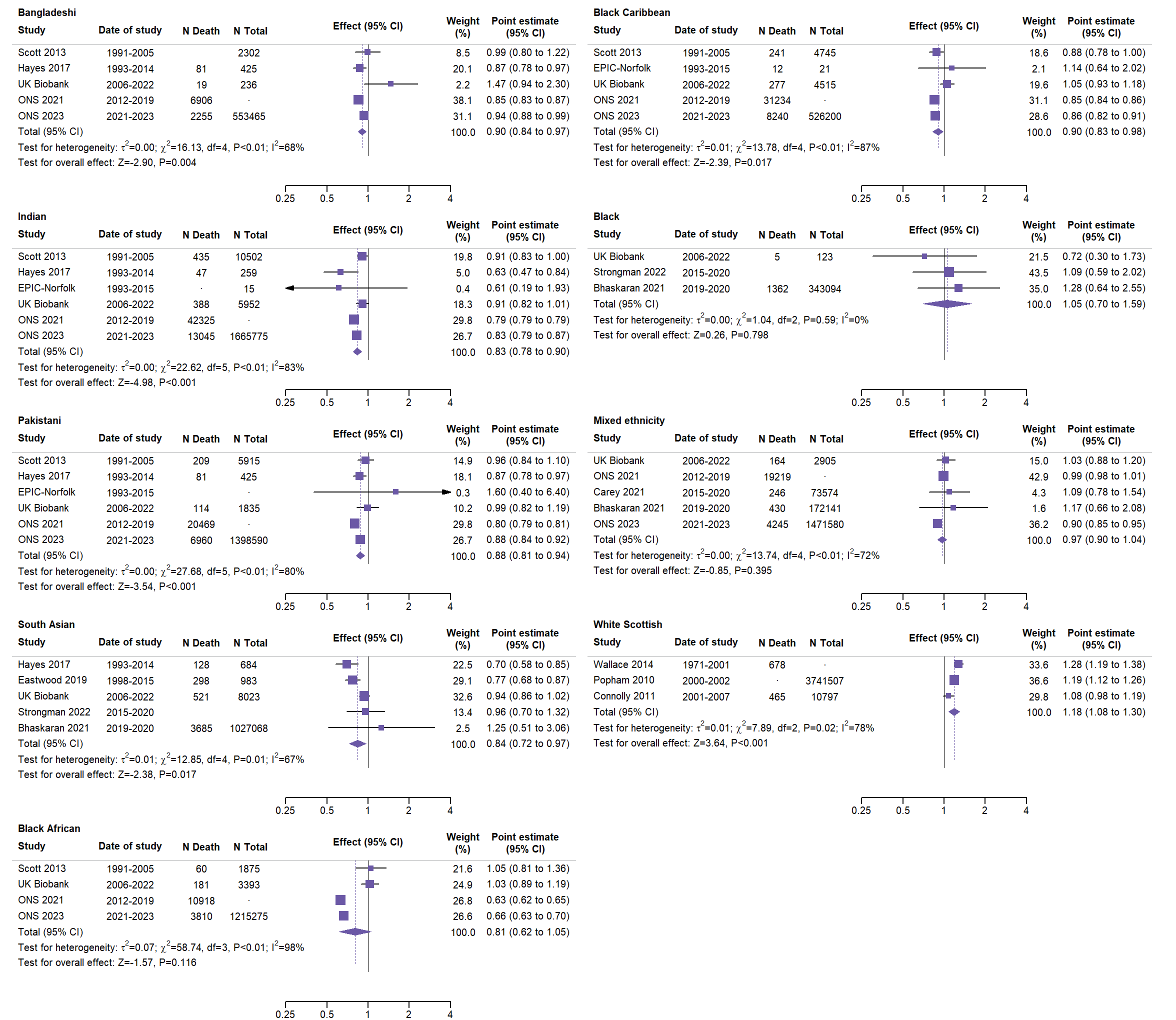
S12a**: Age and sex-adjusted all-cause mortality by ethnicity


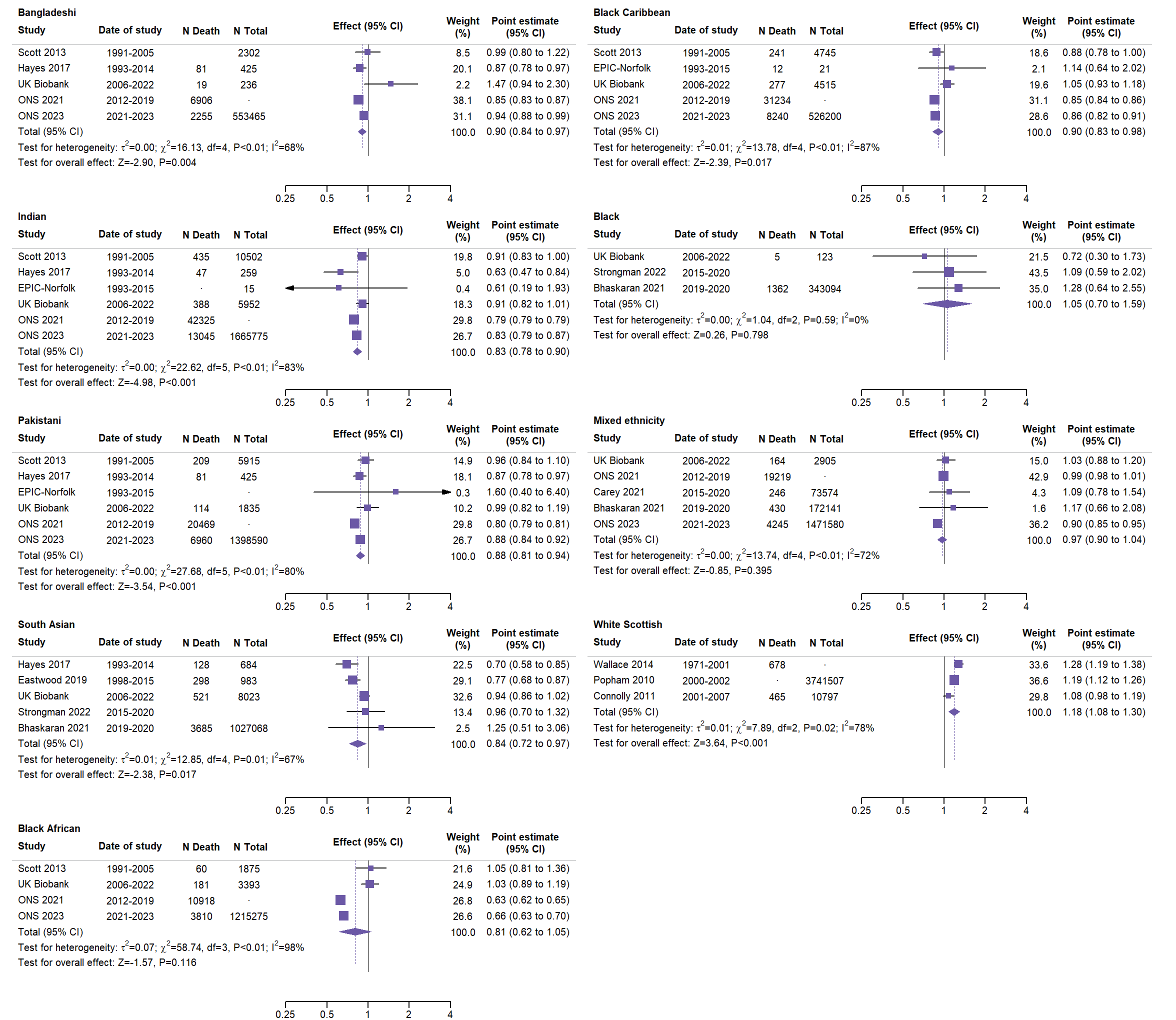

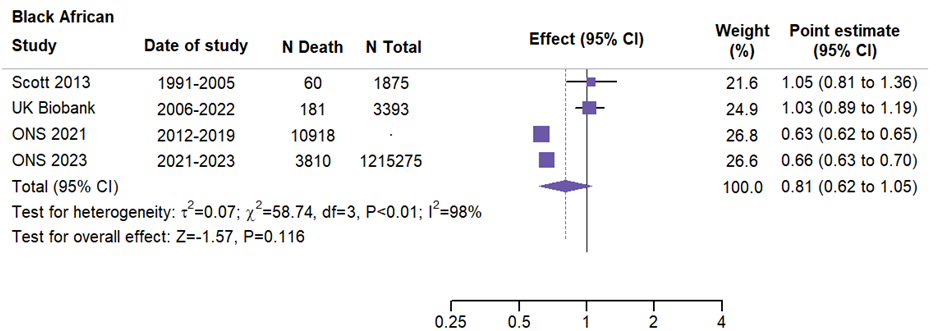


**
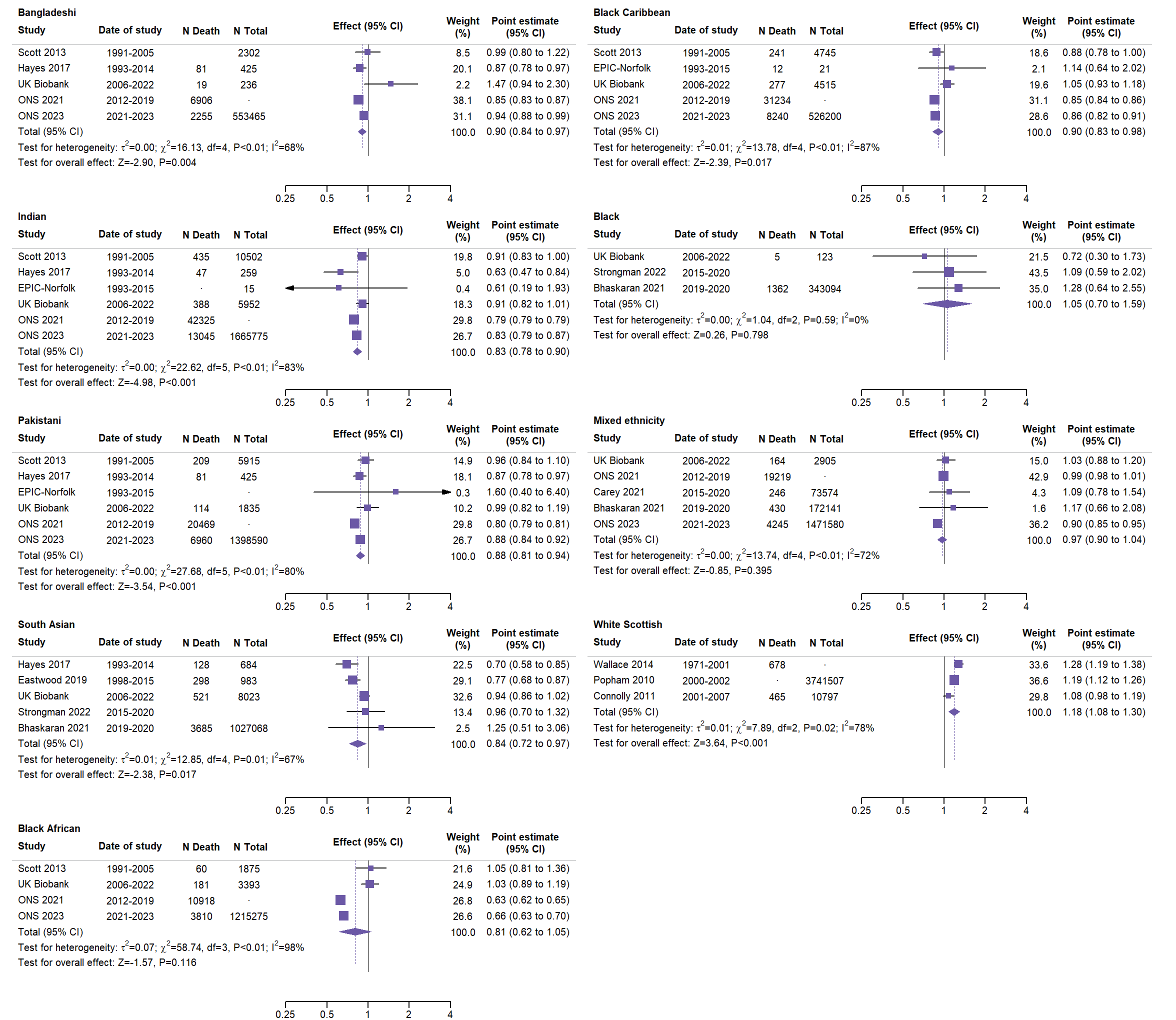
**

**S12b**: Age, sex and SEP-adjusted all-cause mortality by ethnicity


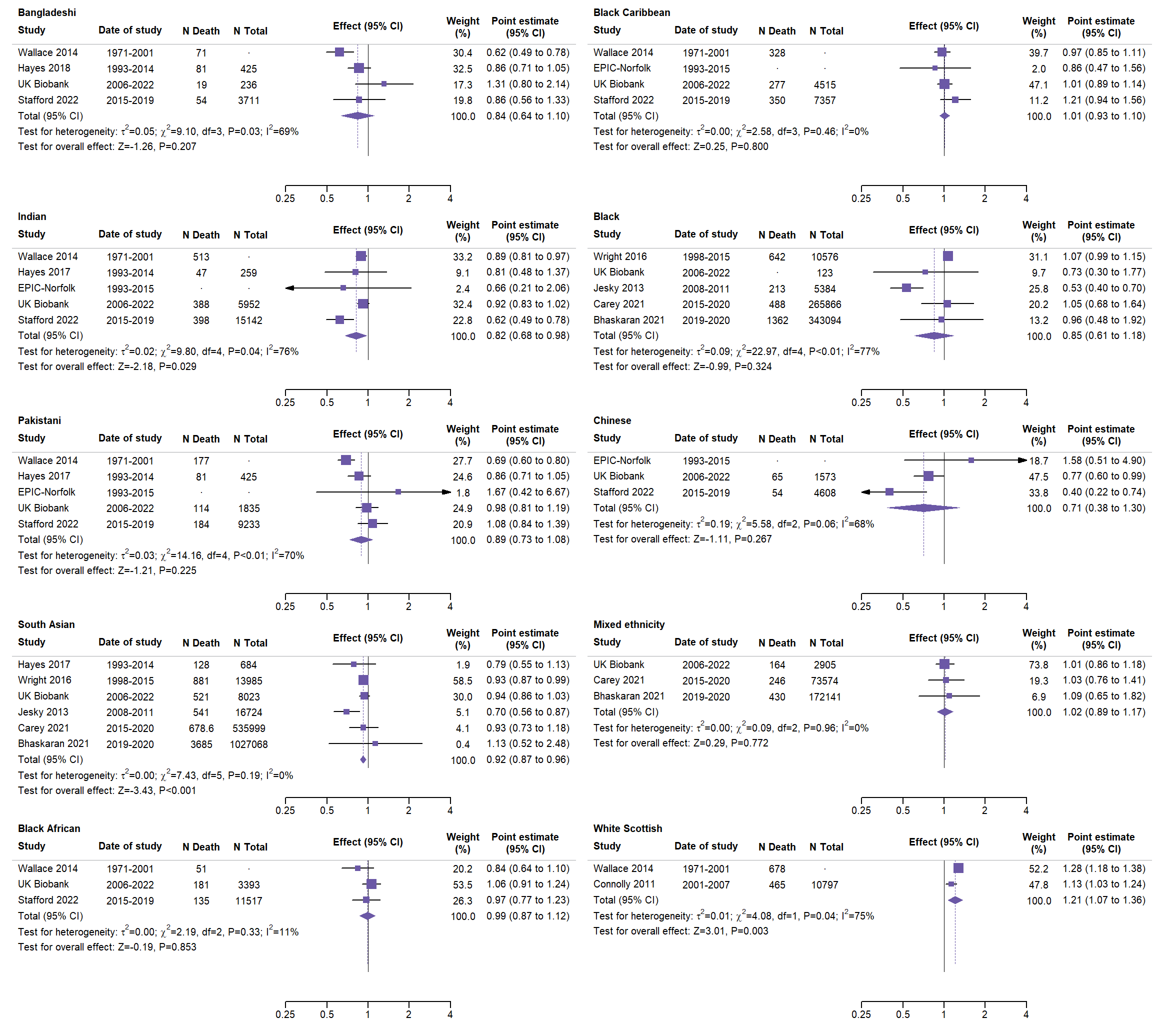


**
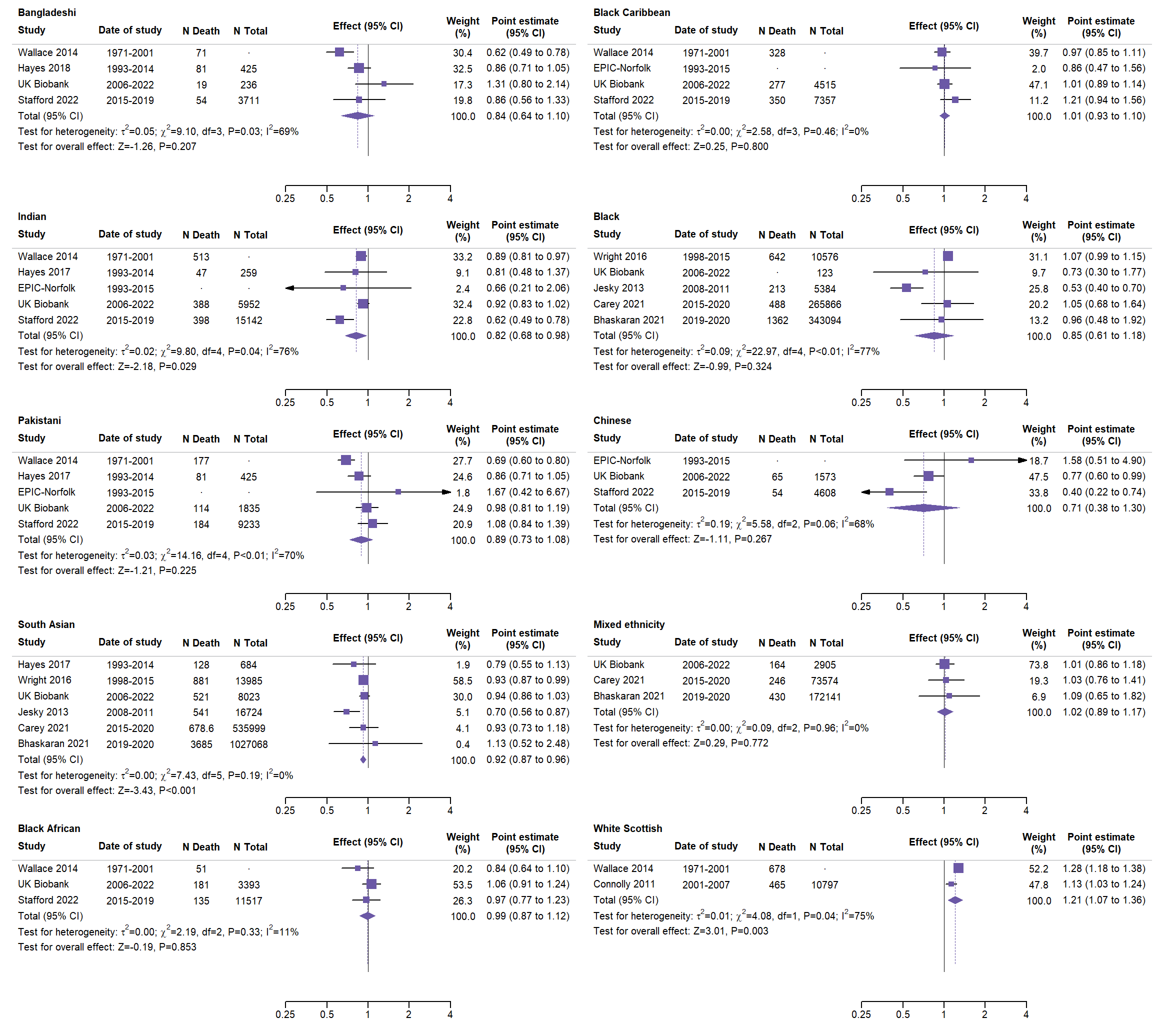
**
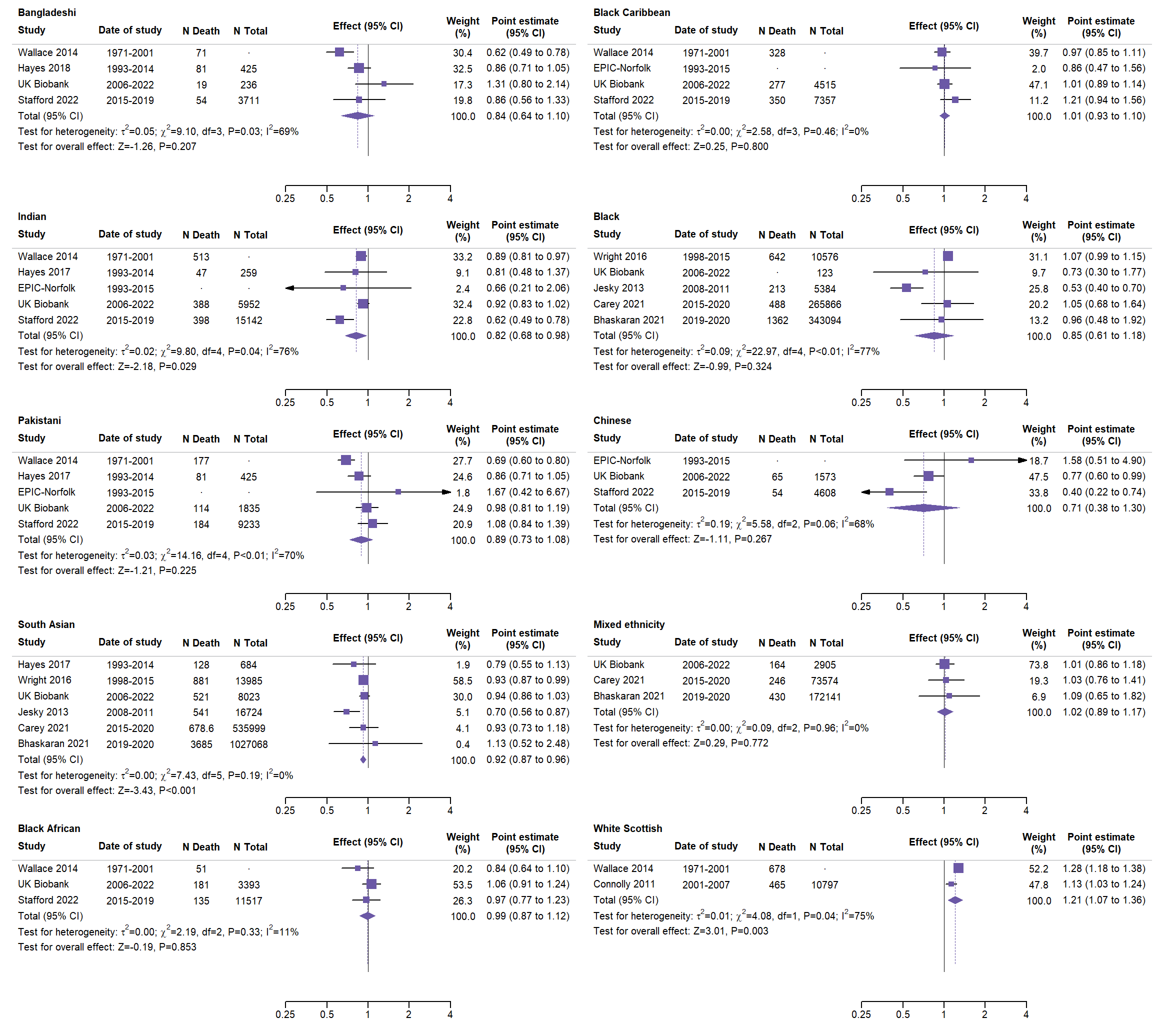


# Supplement 13: Age-adjusted all-cause mortality by ethnicity, restricted to studies with low or some risk of bias

*In males*


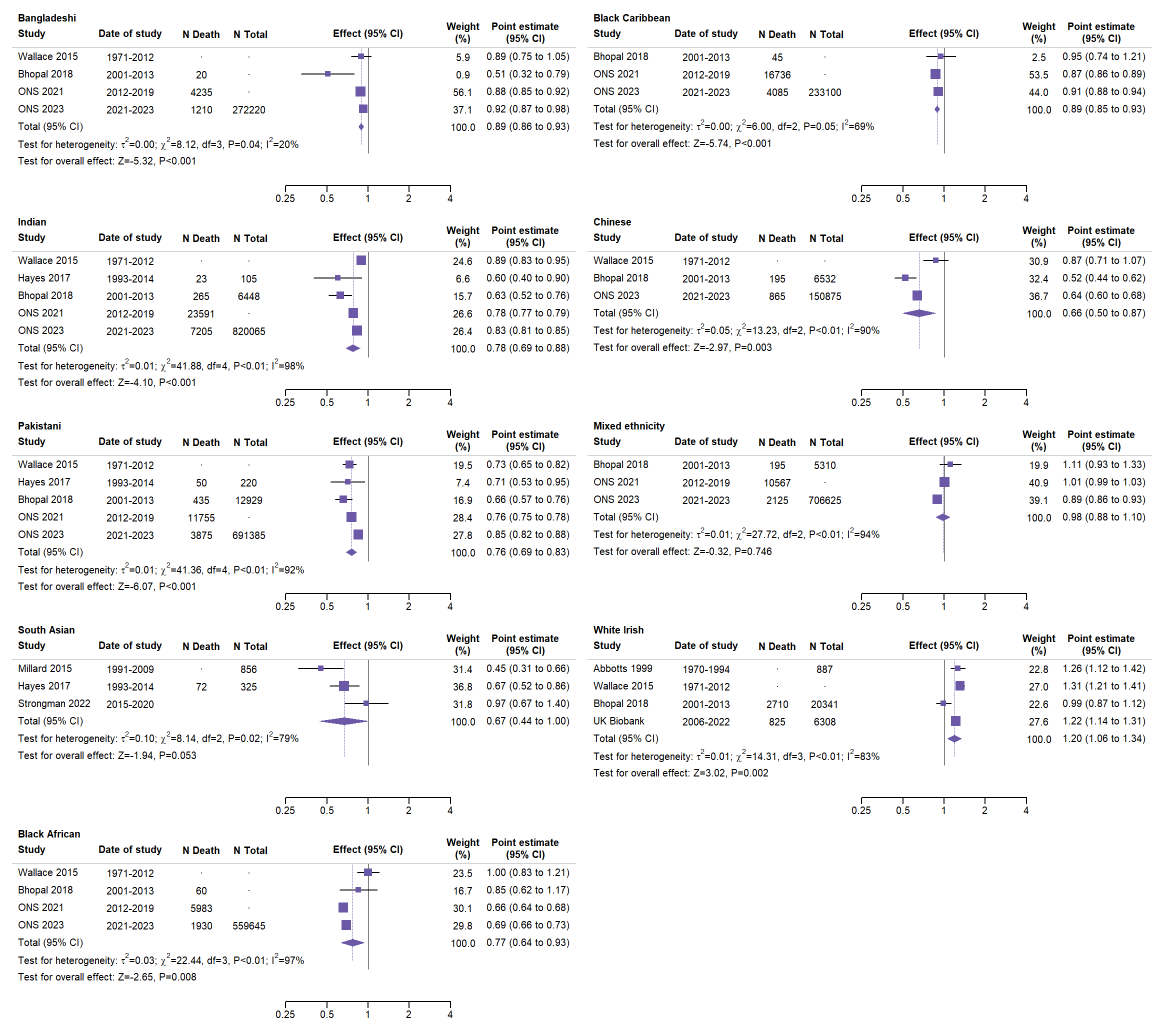


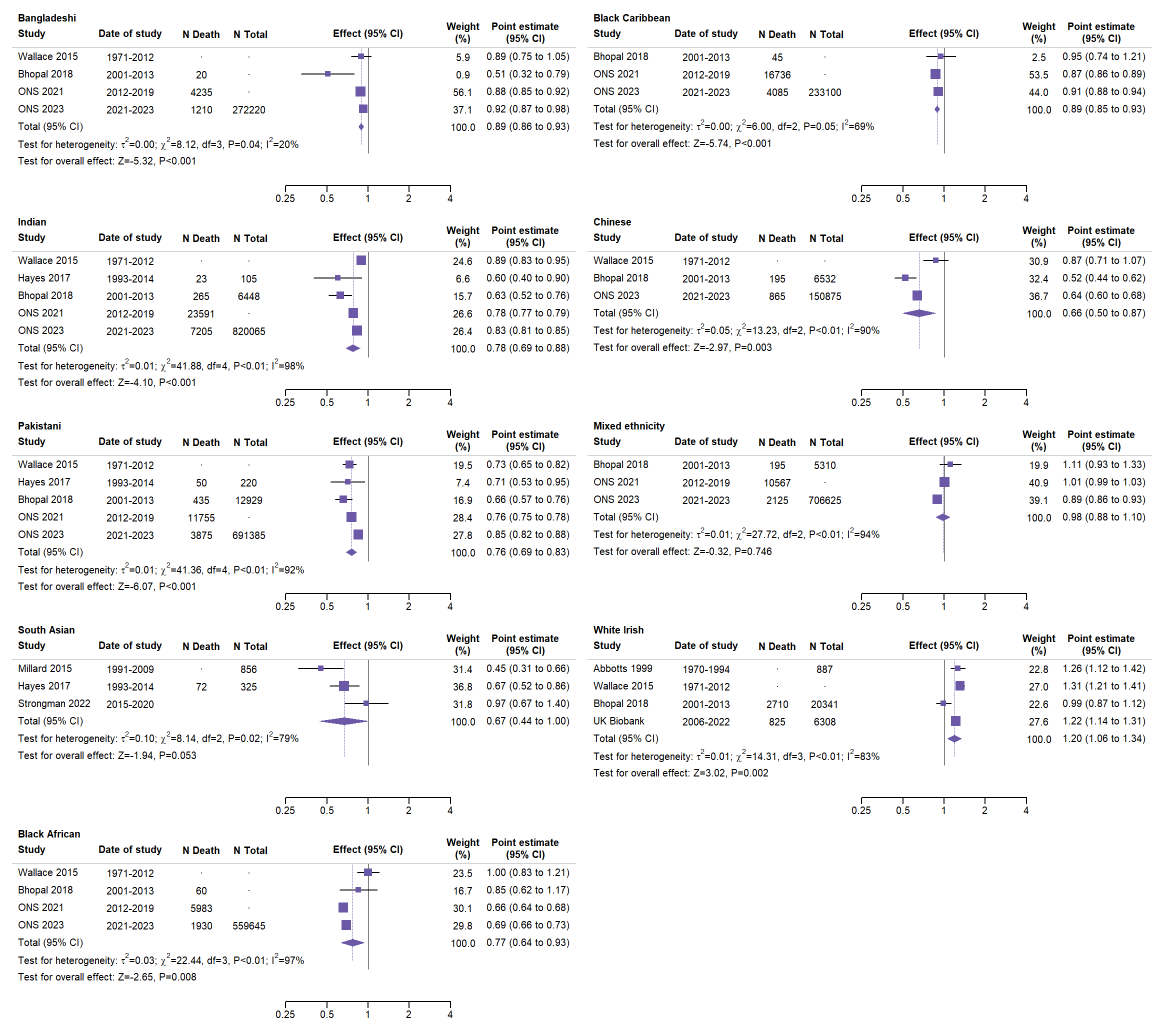

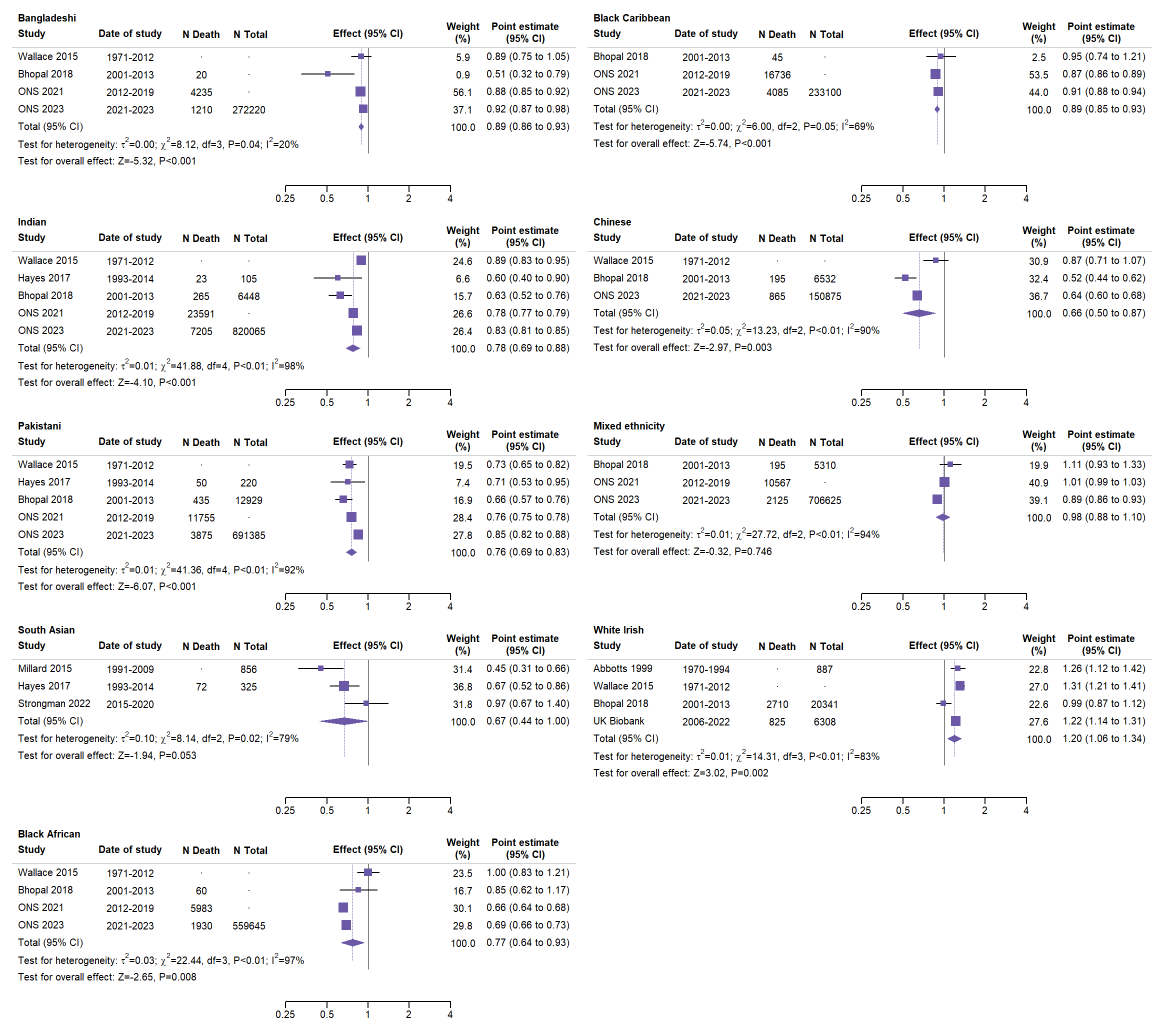


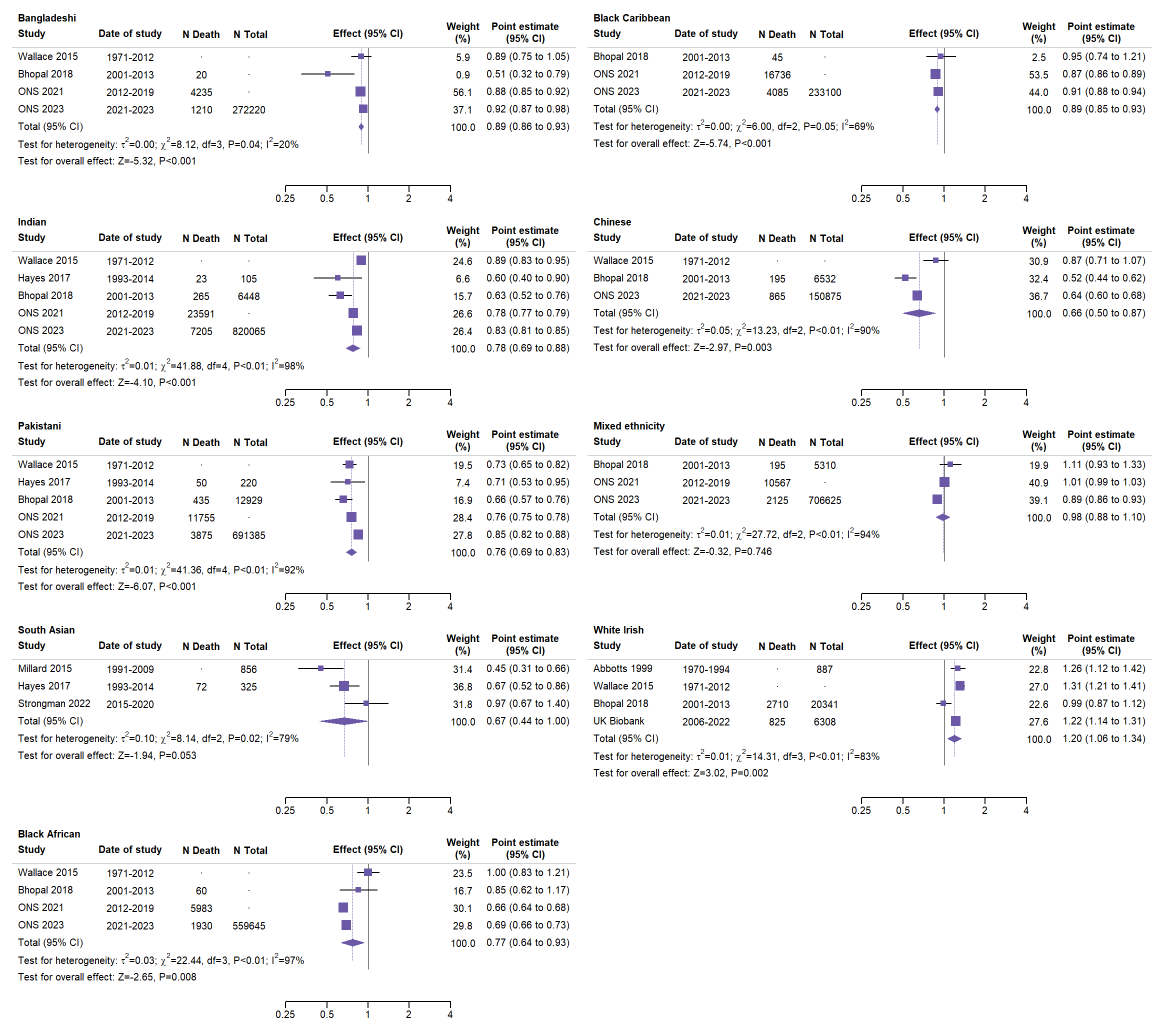


Note: All included studies had a White English/Welsh or total population comparator apart from the following studies with a White Scottish comparator: Bhopal 2018 and Abbotts 1999.


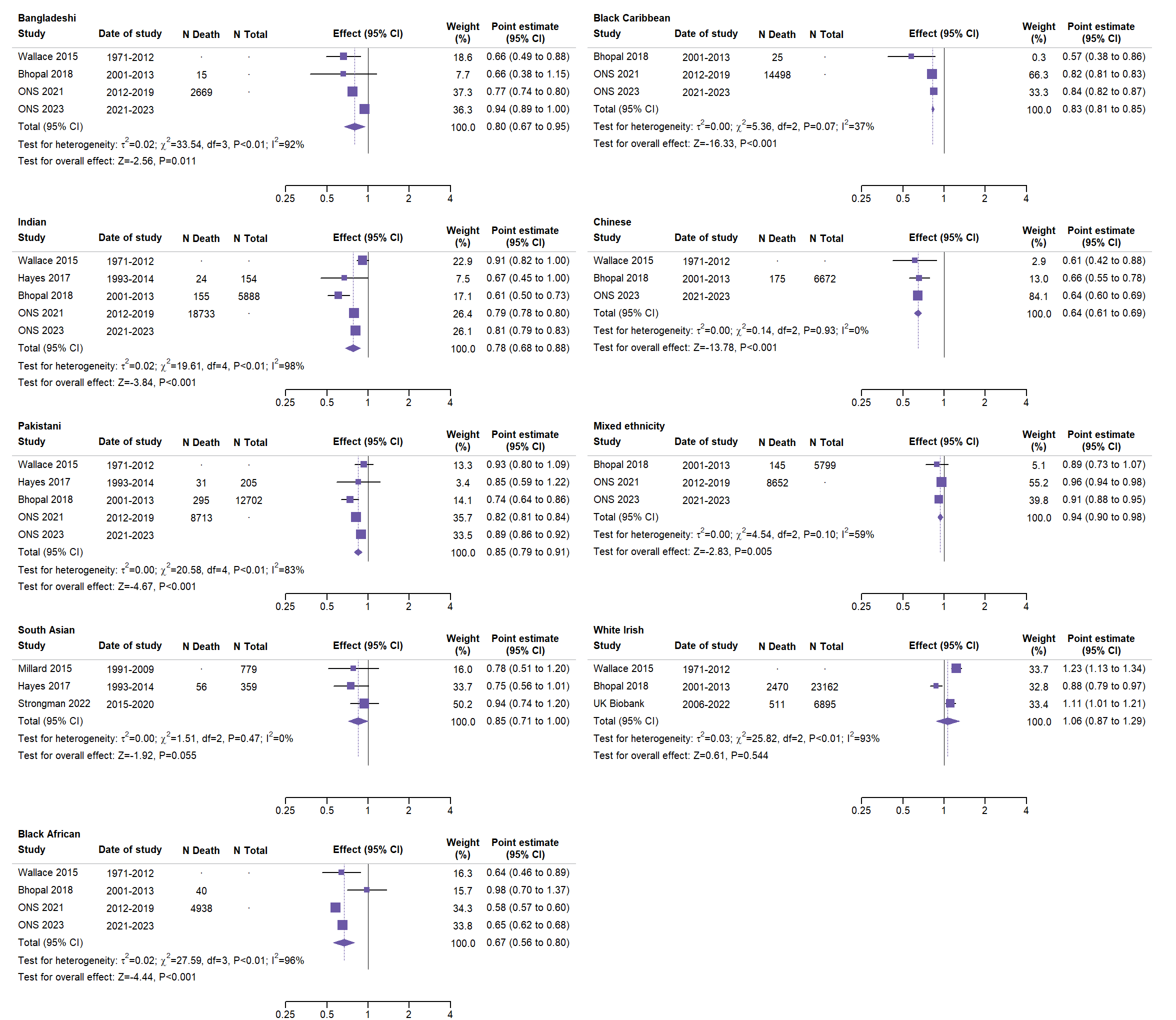
In females


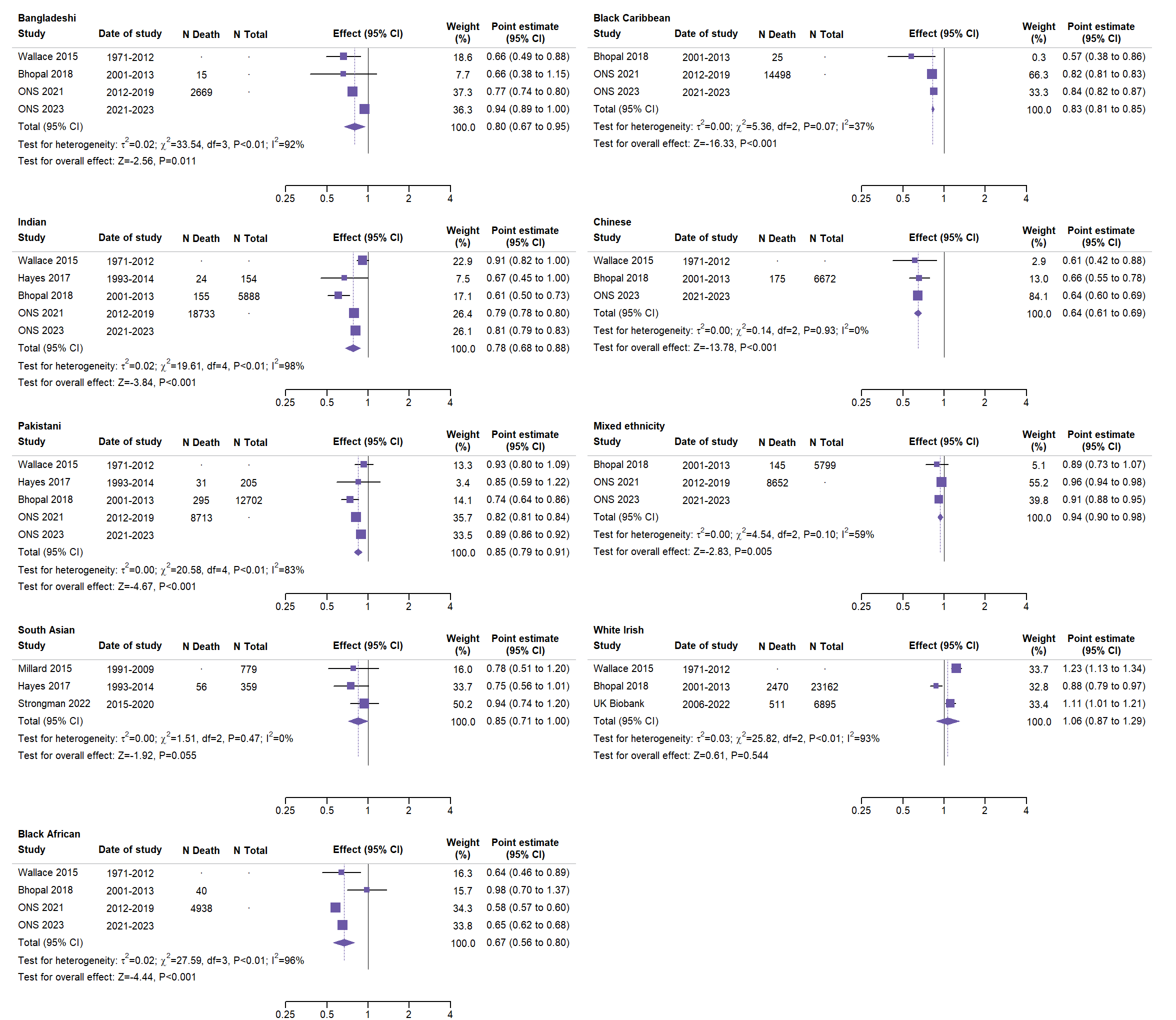

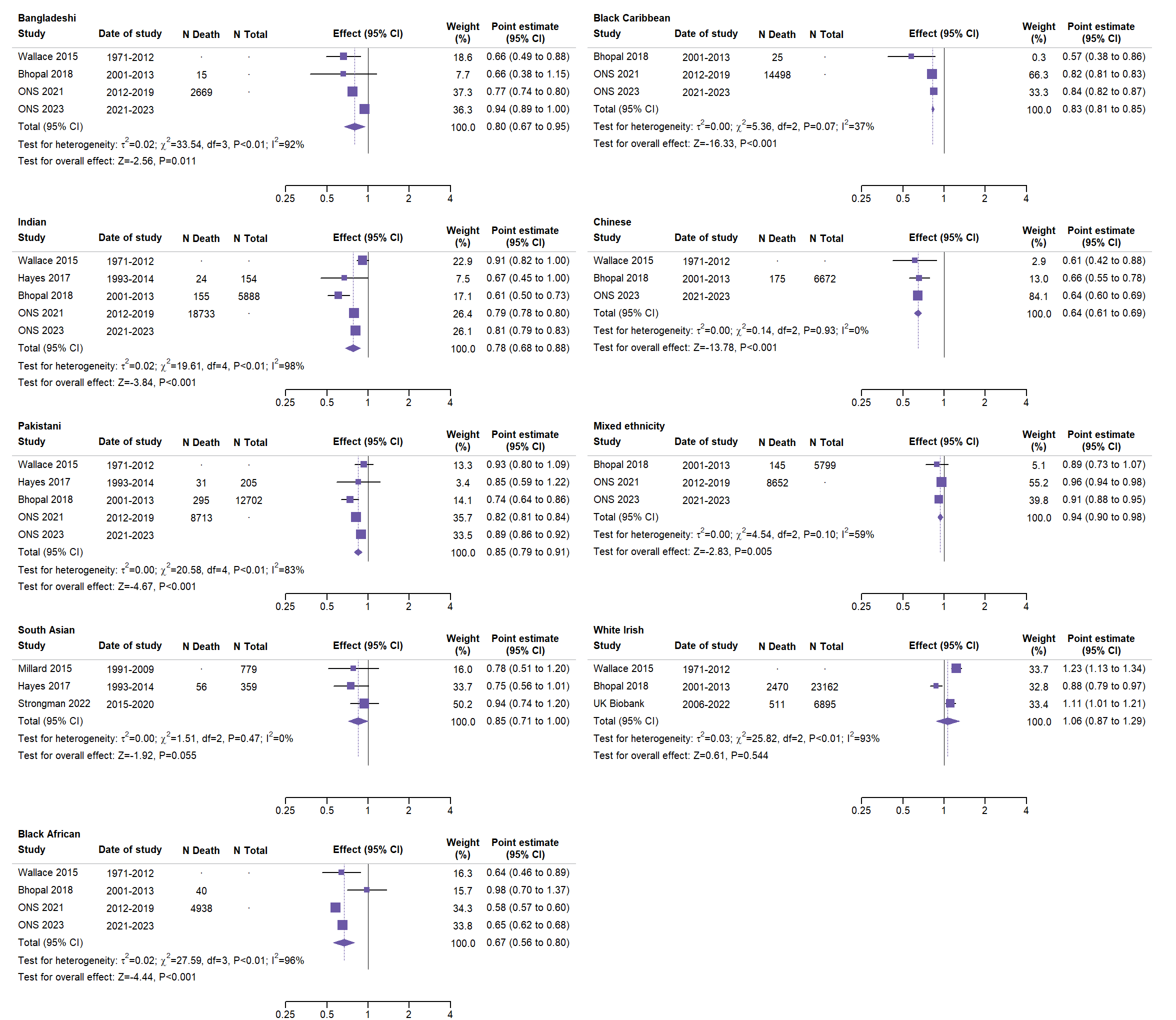


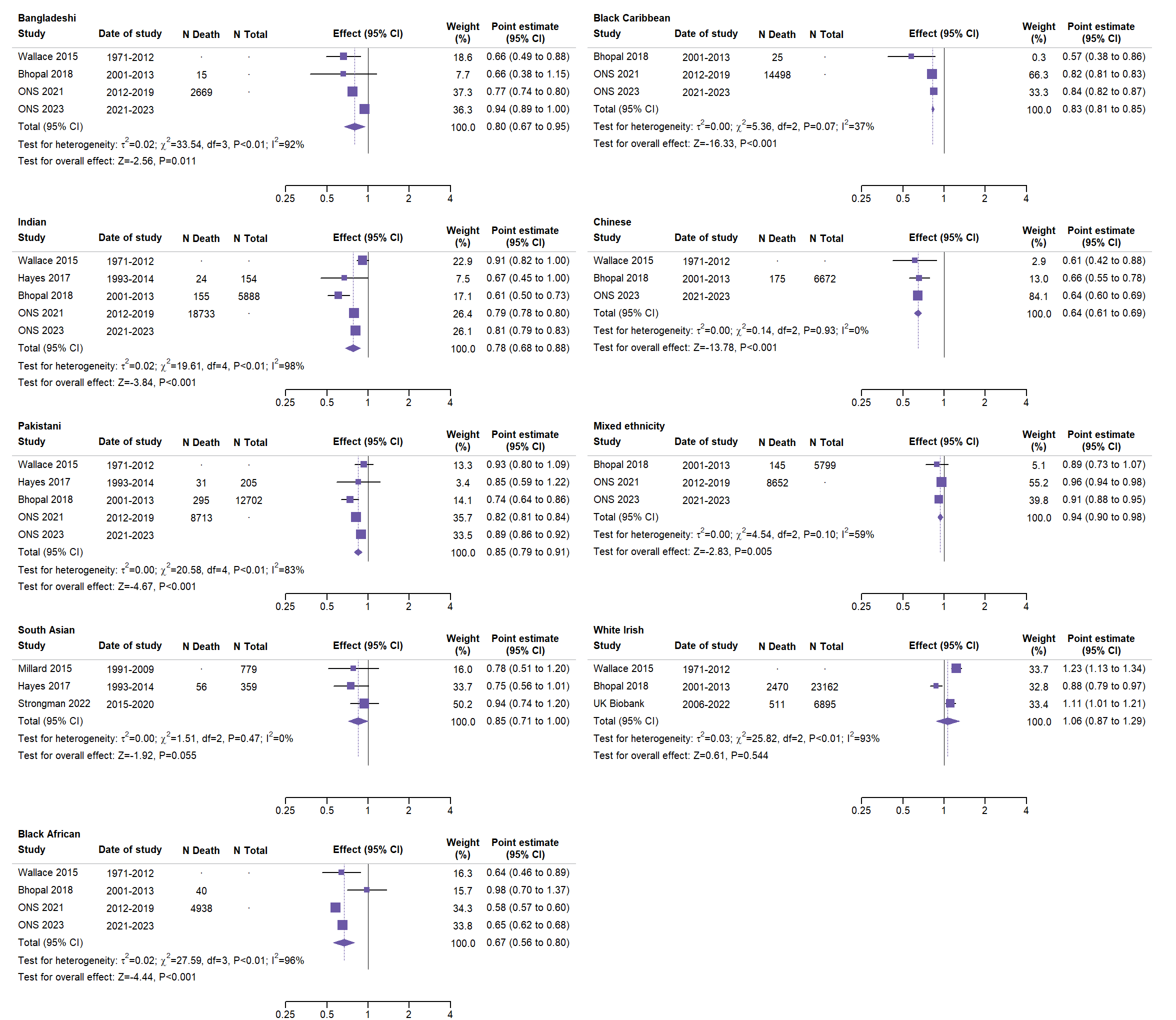


Note: All included studies had a White English/Welsh or total population comparator apart from the following studies with a White Scottish comparator: Bhopal 2018.

# Supplement 14: Sub-group analyses by comparison population (Scottish vs English and Welsh)

This supplementary file contains;

**S14a:** Age adjusted mortality by Bangladeshi ethnicity in males - subgroup analysis by comparison population (Scottish vs English and Welsh)

**S14b:** Age adjusted mortality by Bangladeshi ethnicity in females - subgroup analysis by comparison population (Scottish vs English and Welsh)

**S14c:** Age adjusted mortality by Indian ethnicity in males - subgroup analysis by comparison population (Scottish vs English and Welsh)

**S14d:** Age adjusted mortality by Indian ethnicity in females - subgroup analysis by comparison population (Scottish vs English and Welsh)

**S14e:** Age adjusted mortality by Pakistani ethnicity in males - subgroup analysis by comparison population (Scottish vs English and Welsh)

**S14f:** Age adjusted mortality by Pakistani ethnicity in females - subgroup analysis by comparison population (Scottish vs English and Welsh)

**S14g:** Age adjusted mortality by South Asian ethnicity in males - subgroup analysis by comparison population (Scottish vs English and Welsh)

**S14h:** Age adjusted mortality by South Asian ethnicity in females - subgroup analysis by comparison population (Scottish vs English and Welsh)

**S14i:** Age adjusted mortality by Chinese ethnicity in males - subgroup analysis by comparison population (Scottish vs English and Welsh)

**S14j:** Age adjusted mortality by Chinese ethnicity in females - subgroup analysis by comparison population (Scottish vs English and Welsh)

**S14k:** Age adjusted mortality in males by White Irish ethnicity - subgroup analysis by comparison population (Scottish vs English and Welsh)

**S14l:** Age adjusted mortality in females by White Irish ethnicity - subgroup analysis by comparison population (Scottish vs English and Welsh)

**S14a:** Age adjusted mortality by Bangladeshi ethnicity in males - subgroup analysis by comparison population (Scottish vs English and Welsh)


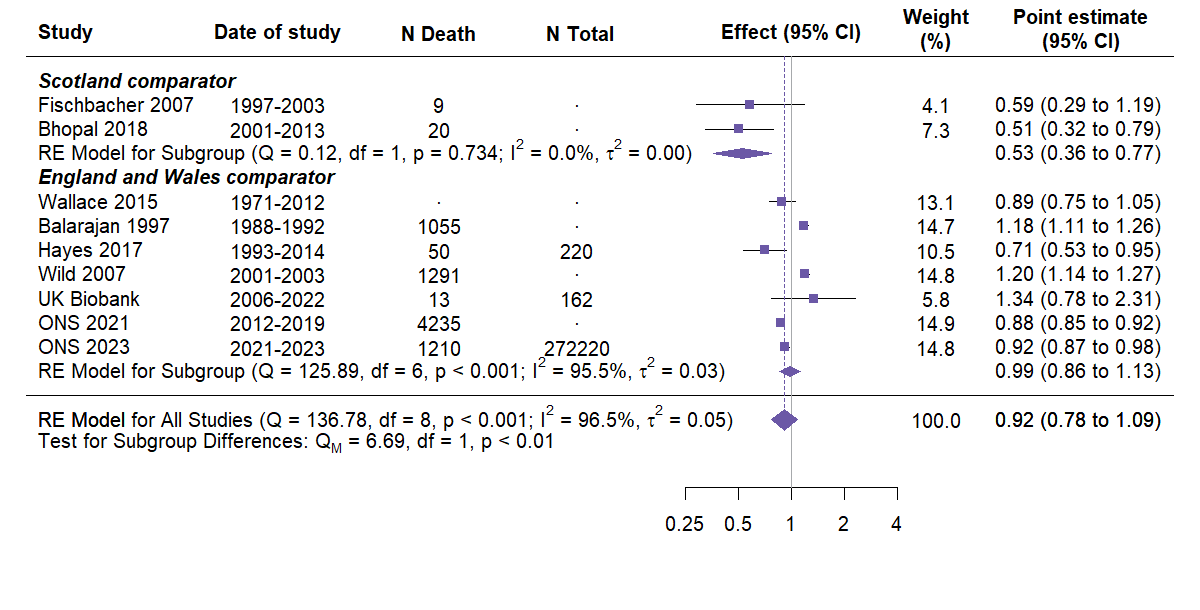


## **S14b:** Age adjusted mortality by Bangladeshi ethnicity in females - subgroup analysis by comparison population (Scottish vs English and Welsh)


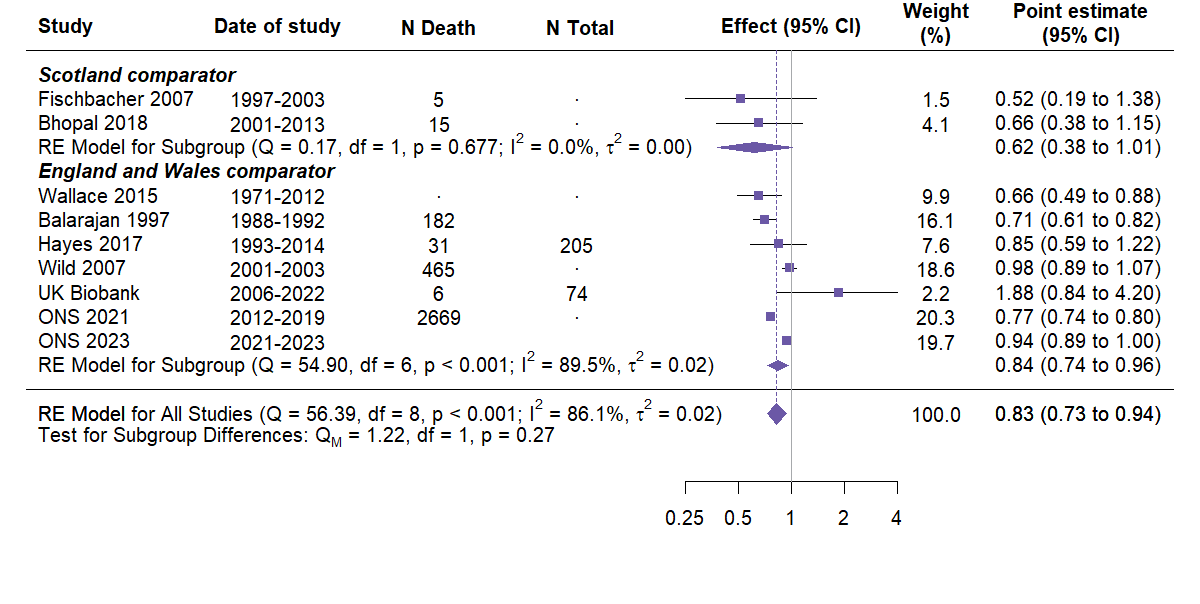


## **S14c:** Age adjusted mortality by Indian ethnicity in males - subgroup analysis by comparison population (Scottish vs English and Welsh)


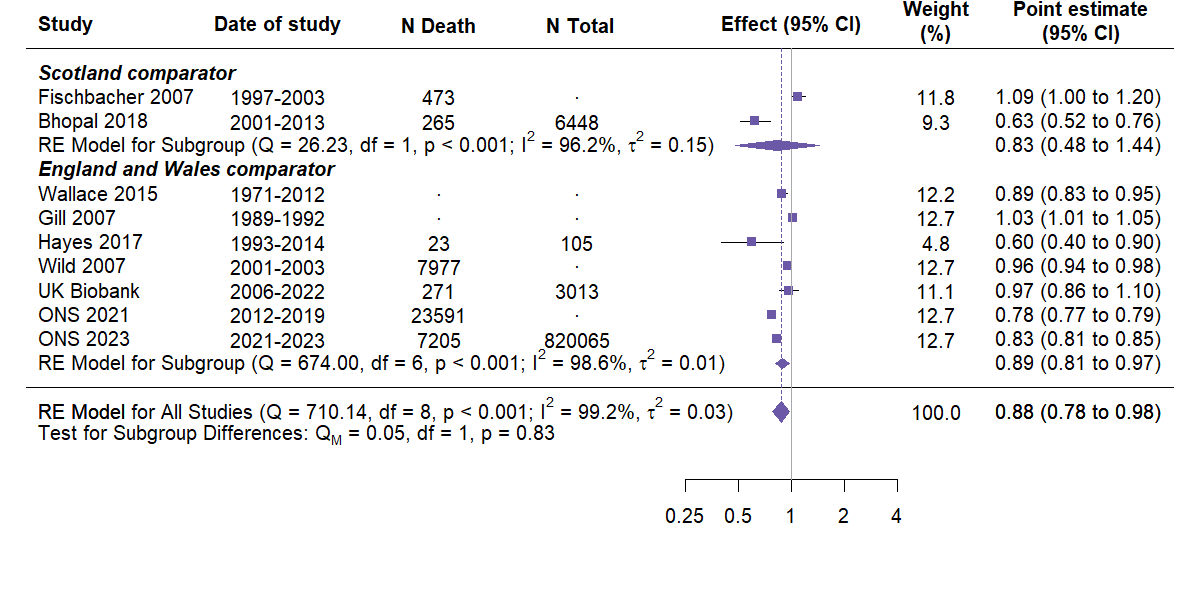


## **S14d:** Age adjusted mortality by Indian ethnicity in females - subgroup analysis by comparison population (Scottish vs English and Welsh)


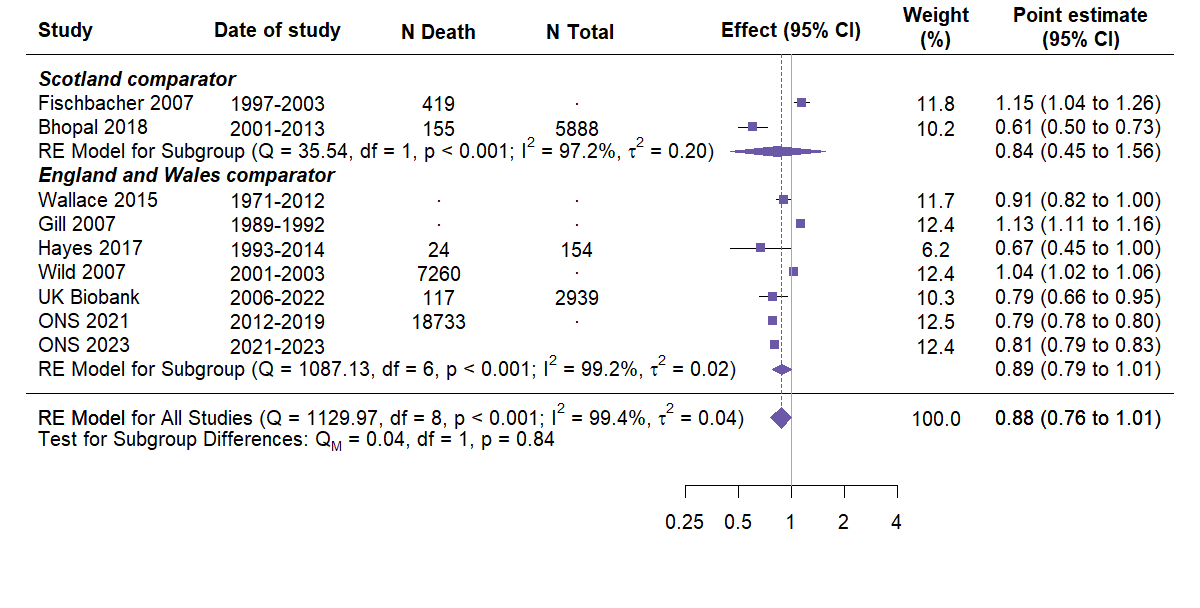


## **S14e:** Age adjusted mortality by Pakistani ethnicity in males - subgroup analysis by comparison population (Scottish vs English and Welsh)


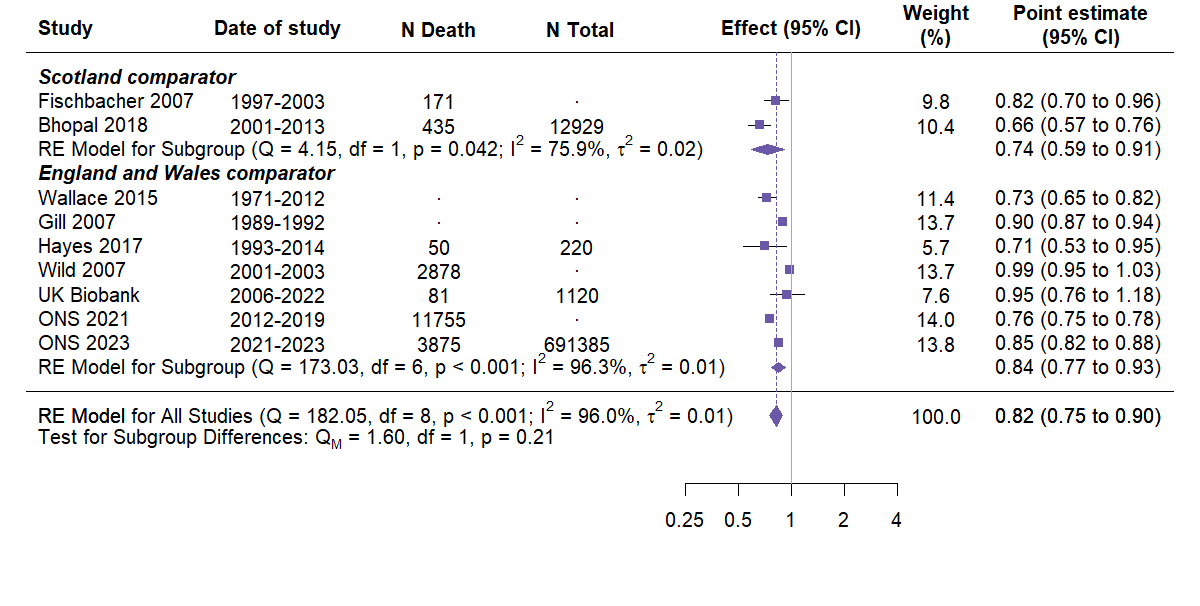


## **S14f:** Age adjusted mortality by Pakistani ethnicity in females - subgroup analysis by comparison population (Scottish vs English and Welsh)


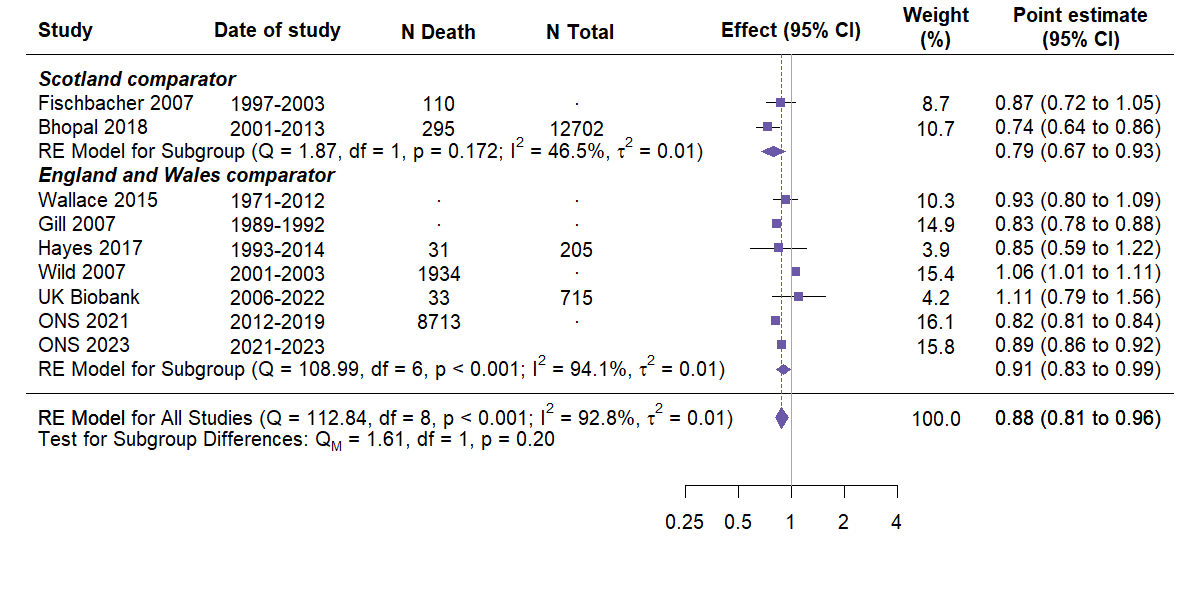


## **S14g:** Age adjusted mortality by Chinese ethnicity in males - subgroup analysis by comparison population (Scottish vs English and Welsh)


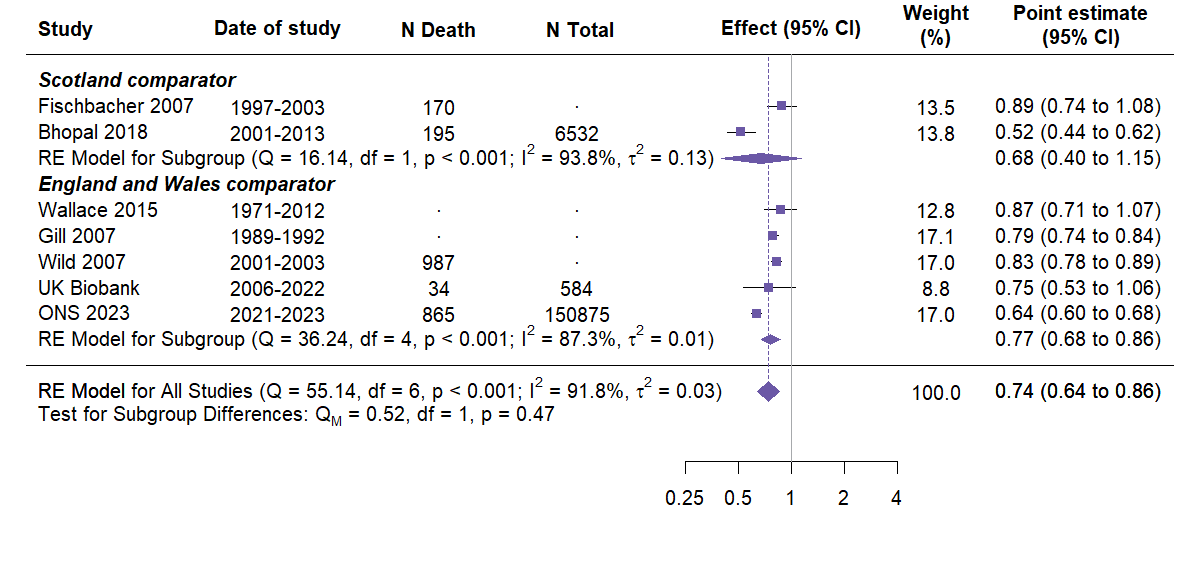


## **S14h:** Age adjusted mortality by Chinese ethnicity in females - subgroup analysis by comparison population (Scottish vs English and Welsh)


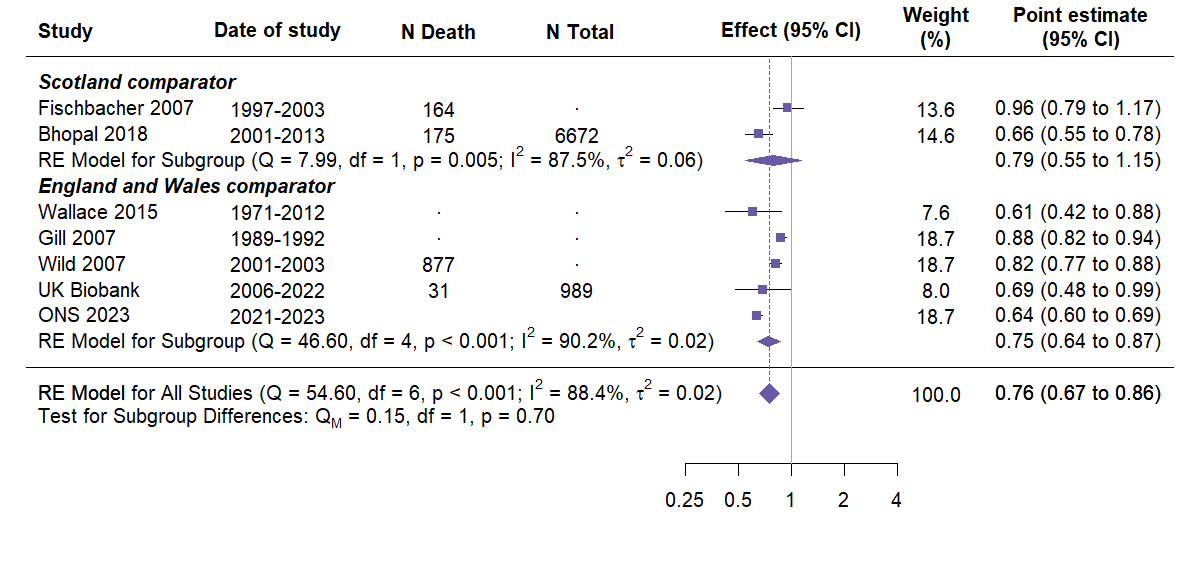


## **S14i:** Age adjusted mortality in males by White Irish ethnicity - subgroup analysis by comparison population (Scottish vs English/Welsh)

**
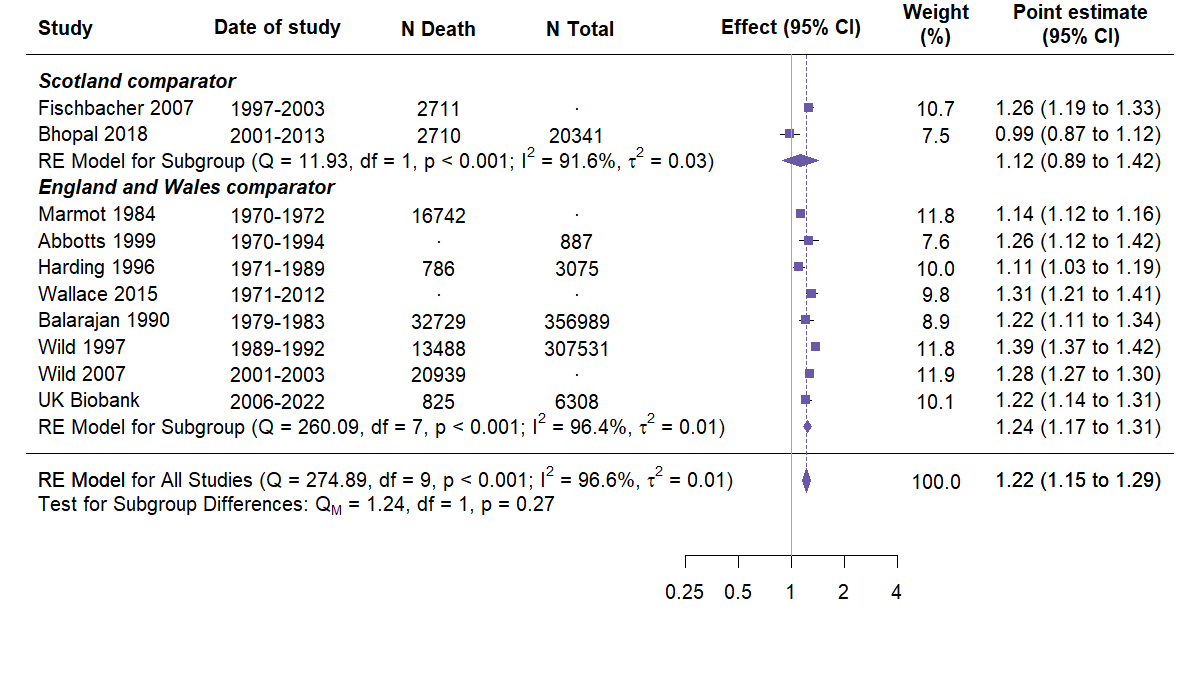
**

## **S14j:** Age adjusted mortality in females by White Irish ethnicity - subgroup analysis by comparison population (Scottish vs English/Welsh)


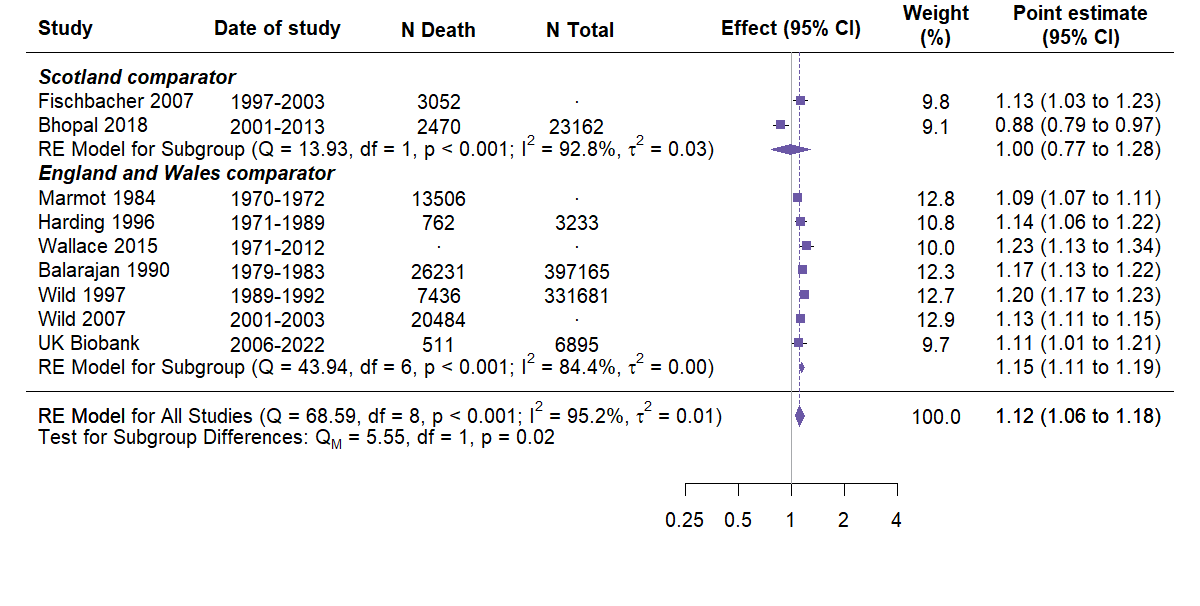


#
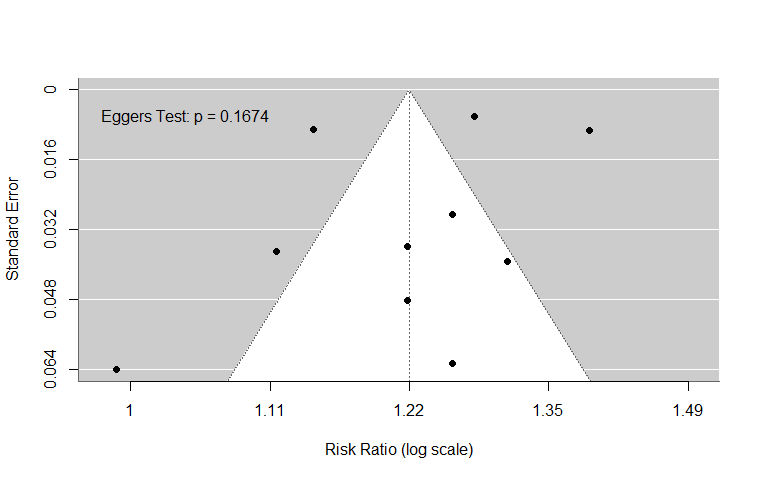
Supplement 15: Funnel plot for assessment of publication bias in age adjusted all-cause mortality in Irish males

# Supplement 16: Risk of bias assessment by analysis and study

| **Study citation** | **Confounding** | **Measurement (exposure*)** | | **Selection** | | | **Post-exposure interventions** | **Missing data** | | **Measurement (outcome)** | **Reporting** | | | **Overall judgement** |
| --- | --- | --- | --- | --- | --- | --- | --- | --- | --- | --- | --- | --- | --- | --- |
| **Age adjusted and sex stratified analyses** | | | | |  |  | | |  |  | |  |  | |
| Abbotts 1999 | LOW | SOME | | LOW | | | LOW | SOME | | LOW | LOW | | | SOME |
| Balarajan 1990 | LOW | VERY HIGH | | LOW | | | LOW | LOW | | LOW | LOW | | | VERY HIGH |
| Balarajan 1997 | LOW | VERY HIGH | | LOW | | | LOW | LOW | | LOW | LOW | | | VERY HIGH |
| Bhopal 2018 | LOW | LOW | | LOW | | | LOW | SOME | | LOW | LOW | | | SOME |
| Fischbacher 2007 | LOW | VERY HIGH | | LOW | | | LOW | LOW | | LOW | LOW | | | VERY HIGH |
| Gill 2007 | LOW | VERY HIGH | | LOW | | | LOW | LOW | | LOW | LOW | | | VERY HIGH |
| Harding 1996 | LOW | HIGH | | LOW | | | LOW | SOME | | LOW | LOW | | | HIGH |
| Hayes 2017 | LOW | HIGH | | SOME | | | LOW | SOME | | LOW | LOW | | | HIGH |
| Ikram 2016 (1) | LOW | HIGH | | LOW | | | LOW | LOW | | LOW | LOW | | | HIGH |
| Marmot 1984 | LOW | VERY HIGH | | LOW | | | LOW | LOW | | LOW | LOW | | | VERY HIGH |
| Millard 2015 | LOW | SOME | | LOW | | | LOW | SOME | | LOW | LOW | | | SOME |
| Million Women Study | LOW | SOME | | HIGH | | | LOW | HIGH | | LOW | NA | | | HIGH |
| ONS 2021 | LOW | LOW | | LOW | | | LOW | SOME | | LOW | LOW | | | SOME |
| ONS 2023 | LOW | LOW | | LOW | | | LOW | LOW | | LOW | LOW | | | LOW |
| Strongman 2022 | LOW | LOW | | LOW | | | LOW | SOME | | LOW | LOW | | | SOME |
| UK Biobank | LOW | LOW | | HIGH | | | LOW | LOW | | LOW | NA | | | HIGH |
| Wallace 2015 | SOME | HIGH | | LOW | | | LOW | SOME | | LOW | LOW | | | HIGH |
| Wild 1997 | LOW | HIGH | | LOW | | | LOW | LOW | | LOW | LOW | | | HIGH |
| Wild 2007 | LOW | VERY HIGH | | LOW | | | LOW | LOW | | LOW | LOW | | | VERY HIGH |
| **Age and sex adjusted analyses** | | |  | |  |  | | |  |  | |  |  | |
| Bhaskaran 2021 | LOW | LOW | | LOW | | | LOW | SOME | | LOW | LOW | | | SOME |
| Carey 2021 | LOW | LOW | | LOW | | | LOW | SOME | | LOW | LOW | | | SOME |
| Connolly 2011 | LOW | SOME | | LOW | | | LOW | SOME | | LOW | LOW | | | SOME |
| Eastwood 2019 | LOW | SOME | | HIGH | | | LOW | HIGH | | LOW | LOW | | | HIGH |
| EPIC Norfolk | LOW | LOW | | HIGH | | | LOW | SOME | | LOW | NA | | | HIGH |
| Hayes 2017 | LOW | HIGH | | SOME | | | LOW | SOME | | LOW | LOW | | | HIGH |
| ONS 2021 | LOW | LOW | | LOW | | | LOW | SOME | | LOW | LOW | | | SOME |
| ONS 2023 | LOW | LOW | | LOW | | | LOW | LOW | | LOW | LOW | | | LOW |
| Popham 2010 | LOW | SOME | | LOW | | | LOW | LOW | | LOW | LOW | | | SOME |
| Scott 2013 | LOW | LOW | | LOW | | | LOW | SOME | | LOW | LOW | | | SOME |
| Strongman 2022 | LOW | LOW | | LOW | | | LOW | SOME | | LOW | LOW | | | SOME |
| UK Biobank | LOW | LOW | | HIGH | | | LOW | LOW | | LOW | NA | | | HIGH |
| Wallace 2014 | LOW | SOME | | LOW | | | LOW | SOME | | LOW | LOW | | | SOME |
| **Age and SEP adjusted analyses stratified by sex** | | | | |  |  | | |  |  | |  |  | |
| Abbotts 1999 | HIGH | SOME | | LOW | | | LOW | SOME | | LOW | LOW | | | HIGH |
| Bhopal 2018 | HIGH | LOW | | LOW | | | LOW | SOME | | LOW | LOW | | | HIGH |
| Harding 1996 | HIGH | HIGH | | LOW | | | LOW | SOME | | LOW | LOW | | | HIGH |
| Harding 1997 | HIGH | VERY HIGH | | LOW | | | LOW | LOW | | LOW | LOW | | | VERY HIGH |
| Hippisley-Cox 2017 | HIGH | LOW | | HIGH | | | LOW | SOME | | LOW | LOW | | | HIGH |
| Lane 2005 | HIGH | LOW | | HIGH | | | LOW | LOW | | LOW | LOW | | | HIGH |
| Marmot 1984 | HIGH | VERY HIGH | | LOW | | | LOW | LOW | | LOW | LOW | | | VERY HIGH |
| Millard 2015 | HIGH | SOME | | LOW | | | LOW | SOME | | LOW | LOW | | | HIGH |
| Million Women Study | HIGH | SOME | | HIGH | | | LOW | HIGH | | LOW | NA | | | HIGH |
| UK Biobank | HIGH | LOW | | HIGH | | | LOW | LOW | | LOW | NA | | | HIGH |
| Wallace 2015 | HIGH | HIGH | | LOW | | | LOW | SOME | | LOW | LOW | | | HIGH |
| **Age, sex and SEP adjusted analyses** | | |  | |  |  | | |  |  | |  |  | |
| Bhaskaran 2021 | HIGH | LOW | | LOW | | | LOW | SOME | | LOW | LOW | | | HIGH |
| Carey 2021 | HIGH | LOW | | LOW | | | LOW | SOME | | LOW | LOW | | | HIGH |
| Connolly 2011 | HIGH | SOME | | LOW | | | LOW | SOME | | LOW | LOW | | | HIGH |
| EPIC Norfolk | HIGH | LOW | | HIGH | | | LOW | SOME | | LOW | NA | | | HIGH |
| Hayes 2017 | HIGH | HIGH | | SOME | | | LOW | SOME | | LOW | LOW | | | HIGH |
| Jesky 2013 | HIGH | LOW | | HIGH | | | LOW | SOME | | LOW | LOW | | | HIGH |
| Stafford 2022 | HIGH | LOW | | LOW | | | LOW | SOME | | LOW | LOW | | | HIGH |
| UK Biobank | HIGH | LOW | | HIGH | | | LOW | LOW | | LOW | NA | | | HIGH |
| Wallace 2014 | HIGH | HIGH | | LOW | | | LOW | SOME | | LOW | LOW | | | HIGH |
| Wright 2017 | HIGH | LOW | | HIGH | | | LOW | SOME | | LOW | LOW | | | HIGH |
| **Country of birth stratified analyses** | | |  | |  |  | | |  |  | |  |  | |
| Bhopal 2018 | LOW | LOW | | LOW | | | LOW | SOME | | LOW | LOW | | | SOME |
| Harding 2001 | LOW | HIGH | | LOW | | | LOW | SOME | | LOW | LOW | | | HIGH |
| Scott 2013 | LOW | LOW | | LOW | | | LOW | SOME | | LOW | LOW | | | SOME |
| UK Biobank | LOW | LOW | | HIGH | | | LOW | LOW | | LOW | NA | | | HIGH |
| Wallace 2016 | LOW | LOW | | LOW | | | LOW | SOME | | LOW | LOW | | | SOME |

*Risk of bias for measurement of exposure in some studies differed by ethnicity as follows:

|  | **Risk of bias for measurement of exposure based on ethnicity** | | | |
| --- | --- | --- | --- | --- |
| **Study citation** | LOW | SOME | HIGH | VERY HIGH |
| Balarajan 1990 | Polish | Black Caribbean  White Irish  White Scottish | - | South Asian  Black African |
| Fischbacher 2007 | - | Chinese  White Irish  White Scottish | - | Bangladeshi  Indian  Pakistani |
| Gill 2007 | - | Black Caribbean  Chinese | Black African | Indian  Pakistani |
| Harding 1997 | - | Black Caribbean  White Scottish  White Irish | South Asian  Black African | Bangladeshi  Indian  Pakistani |
| Hayes 2017 | Indian  South Asian | Pakistani | Bangladeshi | - |
| Marmot 1984 | Polish | Black Caribbean  White Irish  White Scottish | - | South Asian  Black African |
| Wallace 2014 | - | Bangladeshi  Indian  Pakistani  Black African  White Scottish | Black Caribbean | - |
| Wallace 2015 | - | Bangladeshi  Indian  Pakistani  Black African  Chinese  White Irish  White Scottish | Black Caribbean  Polish | - |
| Wild 1997 | - | White Irish  White Scottish | South Asian | - |
| Wild 2007 | - | Chinese  White Irish  White Scottish | - | Bangladeshi  Indian  Pakistani |

# Supplement 17: Detailed GRADE assessment for each ethnic group and analysis

**Question:** What is the relative-risk of all-cause mortality in those of Bangladeshi ethnicity compared to White majority population?

**Setting:** United Kingdom

| **Certainty assessment** | | | | | | **Relative Effect (95% CI)** | **Certainty** | **Comments** |
| --- | --- | --- | --- | --- | --- | --- | --- | --- |
| **№ of studies** | **Risk of bias** | **Inconsistency** | **Indirectness** | **Imprecision** | **Publication bias** |  |  |  |
| **Age-adjusted all-cause mortality in males by Bangladeshi ethnicity** | | | | | | | | |
| 4 | Serious | Not Serious | Not Serious | Not Serious | Undetected | **RR 0.89** (0.86 to 0.93) | ⨁⨁⨁◯ Moderate |  |
| **Age-adjusted all-cause mortality in females by Bangladeshi ethnicity** | | | | | | | | |
| 4 | Serious | Not Serious | Not Serious | Not Serious | Undetected | **RR 0.80** (0.67 to 0.95) | ⨁⨁⨁◯ Moderate |  |
| **Question:** To what extent do inequalities in SEP account for observed differences in the relative risk of all-cause mortality in those of Bangladeshi ethnicity?  **Age and SEP adjusted all-cause mortality in males by Bangladeshi ethnicity** | | | | | | | | |
| 5 | Very Serious | Serious | Serious | Serious | Undetected | **RR 0.90** (0.61 to 1.32) | ⨁◯◯◯ Very low |  |
| **Age and SEP adjusted all-cause mortality in females by Bangladeshi ethnicity** | | | | | | | | |
| 4 | Very Serious | Serious | Serious | Serious | Undetected | **RR 0.74** (0.44 to 1.24) | ⨁◯◯◯ Very low |  |
| **Question:** Does the relative risk of all-cause mortality in the Bangladeshi population compared to the White British population differ by country of birth (UK-born vs born outside of the UK)?  **Age and sex adjusted all-cause mortality by Bangladeshi ethnicity stratified by country of birth (UK vs non UK)** | | | | | | | | |
| 3 | Serious | Serious | Not Serious | Serious | Undetected | Bangladeshi UK-born: **RR 1.11** (0.50 - 2.44)  Bangladeshi non-UK born: **RR 0.74** (0.35 - 1.58) | ⨁◯◯◯ Very low |  |

**Question:** What is the relative-risk of all-cause mortality in those of Indian ethnicity compared to White majority population?

**Setting:** United Kingdom

| **Certainty assessment** | | | | | | **Relative Effect (95% CI)** | **Certainty** | **Comments** |
| --- | --- | --- | --- | --- | --- | --- | --- | --- |
| **№ of studies** | **Risk of bias** | **Inconsistency** | **Indirectness** | **Imprecision** | **Publication bias** |  |  |  |
| **Age-adjusted all-cause mortality in males by Indian ethnicity** | | | | | | | | |
| 5 | Serious | Not Serious | Not Serious | Not Serious | Undetected | **RR 0.78** (0.69 to 0.88) | ⨁⨁⨁◯ Moderate |  |
| **Age-adjusted all-cause mortality in females by Indian ethnicity** | | | | | | | | |
| 5 | Serious | Not Serious | Not Serious | Not Serious | Undetected | **RR 0.78** (0.68 to 0.88) | ⨁⨁⨁◯ Moderate |  |
| **Question:** To what extent do inequalities in SEP account for observed differences in the relative risk of all-cause mortality in those of Indian ethnicity?  **Age and SEP adjusted all-cause mortality in males by Indian ethnicity** | | | | | | | | |
| 5 | Very Serious | Serious | Serious | Serious | Undetected | **RR 0.90** (0.77 to 1.05) | ⨁◯◯◯ Very low |  |
| **Age and SEP adjusted all-cause mortality in females by Indian ethnicity** | | | | | | | | |
| 4 | Very Serious | Not Serious | Serious | Not Serious | Undetected | **RR 0.79** (0.71 to 0.88) | ⨁◯◯◯ Very low |  |
| **Question:** Does the relative risk of all-cause mortality in the Indian population compared to the White British population differ by country of birth (UK-born vs born outside of the UK)?  **Age and sex adjusted all-cause mortality by Indian ethnicity stratified by country of birth (UK vs non UK)** | | | | | | | | |
| 3 | Serious | Serious | Not Serious | Serious | Undetected | Indian UK-born: **RR 0.89** (0.73 – 1.09)  Indian non-UK born: **RR 0.77** (0.58 - 1.02) | ⨁◯◯◯ Very low |  |

**Question:** What is the relative-risk of all-cause mortality in those of Pakistani ethnicity compared to White majority population?

**Setting:** United Kingdom

| **Certainty assessment** | | | | | | **Relative Effect (95% CI)** | **Certainty** | **Comments** |
| --- | --- | --- | --- | --- | --- | --- | --- | --- |
| **№ of studies** | **Risk of bias** | **Inconsistency** | **Indirectness** | **Imprecision** | **Publication bias** |  |  |  |
| **Age-adjusted all-cause mortality in males by Pakistani ethnicity** | | | | | | | | |
| 5 | Serious | Not Serious | Not Serious | Not Serious | Undetected | **RR 0.79** (0.69 to 0.83) | ⨁⨁⨁◯ Moderate |  |
| **Age-adjusted all-cause mortality in females by Pakistani ethnicity** | | | | | | | | |
| 5 | Serious | Not Serious | Not Serious | Not Serious | Undetected | **RR 0.85** (0.79 to 0.91) | ⨁⨁⨁◯ Moderate |  |
| **Question:** To what extent do inequalities in SEP account for observed differences in the relative risk of all-cause mortality in those of Pakistani ethnicity?  **Age and SEP adjusted all-cause mortality in males by Pakistani ethnicity** | | | | | | | | |
| 5 | Very Serious | Serious | Serious | Serious | Undetected | **RR 0.81** (0.67 to 1.00) | ⨁◯◯◯ Very low |  |
| **Age and SEP adjusted all-cause mortality in females by Pakistani ethnicity** | | | | | | | | |
| 4 | Very Serious | Serious | Serious | Serious | Undetected | **RR 0.84** (0.66 to 1.07) | ⨁◯◯◯ Very low |  |
| **Question:** Does the relative risk of all-cause mortality in the Pakistani population compared to the White British population differ by country of birth (UK-born vs born outside of the UK)?  **Age and sex adjusted all-cause mortality by Pakistani ethnicity stratified by country of birth (UK vs non UK)** | | | | | | | | |
| 3 | Serious | Serious | Not Serious | Serious | Undetected | Pakistani UK-born: **RR 0.82** (0.63 – 1.05)  Pakistani non-UK born: **RR 0.87** (0.68 - 1.11) | ⨁◯◯◯ Very low |  |

**Question:** What is the relative-risk of all-cause mortality in those of Black African ethnicity compared to White majority population?

**Setting:** United Kingdom

| **Certainty assessment** | | | | | | **Relative Effect (95% CI)** | **Certainty** | **Comments** |
| --- | --- | --- | --- | --- | --- | --- | --- | --- |
| **№ of studies** | **Risk of bias** | **Inconsistency** | **Indirectness** | **Imprecision** | **Publication bias** |  |  |  |
| **Age-adjusted all-cause mortality in males by Black African ethnicity** | | | | | | | | |
| 4 | Serious | Serious | Not Serious | Not Serious | Undetected | **RR 0.77** (0.64 to 0.93) | ⨁⨁◯◯ Low |  |
| **Age-adjusted all-cause mortality in females by Black African ethnicity** | | | | | | | | |
| 4 | Serious | Serious | Not Serious | Not Serious | Undetected | **RR 0.67** (0.56 to 0.80) | ⨁⨁◯◯ Low |  |
| **Question:** To what extent do inequalities in SEP account for observed differences in the relative risk of all-cause mortality in those of Black African ethnicity?  **Age and SEP adjusted all-cause mortality in males by Black African ethnicity** | | | | | | | | |
| 6 | Very Serious | Serious | Serious | Serious | Undetected | **RR 0.98** (0.79 to 1.21) | ⨁◯◯◯ Very low |  |
| **Age and SEP adjusted all-cause mortality in females by Black African ethnicity** | | | | | | | | |
| 6 | Very Serious | Serious | Serious | Serious | Undetected | **RR 0.90** (0.68 to 1.20) | ⨁◯◯◯ Very low |  |
| **Question:** Does the relative risk of all-cause mortality in the Black African population compared to the White British population differ by country of birth (UK-born vs born outside of the UK)?  **Age and sex adjusted all-cause mortality by Black African ethnicity stratified by country of birth (UK vs non UK)** | | | | | | | | |
| 3 | Serious | Serious | Not Serious | Serious | Undetected | Black African UK-born: **RR 1.23** (0.84 – 1.81)  Black African non-UK born: **RR 0.85** (0.68 - 1.06) | ⨁◯◯◯ Very low |  |

**Question:** What is the relative-risk of all-cause mortality in those of Black Caribbean ethnicity compared to White majority population?

**Setting:** United Kingdom

| **Certainty assessment** | | | | | | **Relative Effect (95% CI)** | **Certainty** | **Comments** |
| --- | --- | --- | --- | --- | --- | --- | --- | --- |
| **№ of studies** | **Risk of bias** | **Inconsistency** | **Indirectness** | **Imprecision** | **Publication bias** |  |  |  |
| **Age-adjusted all-cause mortality in males by Black Caribbean ethnicity** | | | | | | | | |
| 3 | Serious | Not Serious | Not Serious | Not Serious | Undetected | **RR 0.89** (0.85 to 0.93) | ⨁⨁⨁◯ Moderate |  |
| **Age-adjusted all-cause mortality in females by Black Caribbean ethnicity** | | | | | | | | |
| 3 | Serious | Not Serious | Not Serious | Not Serious | Undetected | **RR 0.83** (0.81 to 0.85) | ⨁⨁⨁◯ Moderate |  |
| **Question:** To what extent do inequalities in SEP account for observed differences in the relative risk of all-cause mortality in those of Black Caribbean ethnicity?  **Age and SEP adjusted all-cause mortality in males by Black Caribbean ethnicity** | | | | | | | | |
| 6 | Very Serious | Not Serious | Serious | Not Serious | Undetected | **RR 0.81** (0.72 to 0.90) | ⨁◯◯◯ Very low |  |
| **Age and SEP adjusted all-cause mortality in females by Black Caribbean ethnicity** | | | | | | | | |
| 6 | Very Serious | Serious | Serious | Serious | Undetected | **RR 0.88** (0.69 to 1.13) | ⨁◯◯◯ Very low |  |
| **Question:** Does the relative risk of all-cause mortality in the Black Caribbean population compared to the White British population differ by country of birth (UK-born vs born outside of the UK)?  **Age and sex adjusted all-cause mortality by Black Caribbean ethnicity stratified by country of birth (UK vs non UK)** | | | | | | | | |
| 3 | Serious | Serious | Not Serious | Serious | Undetected | Black Caribbean UK-born: **RR 1.49** (0.98 – 2.28)  Black Caribbean non-UK born: **RR 0.95** (0.82 - 1.10) | ⨁◯◯◯ Very low |  |

**Question:** What is the relative-risk of all-cause mortality in those of Chinese ethnicity compared to White majority population?

**Setting:** United Kingdom

| **Certainty assessment** | | | | | | **Relative Effect (95% CI)** | **Certainty** | **Comments** |
| --- | --- | --- | --- | --- | --- | --- | --- | --- |
| **№ of studies** | **Risk of bias** | **Inconsistency** | **Indirectness** | **Imprecision** | **Publication bias** |  |  |  |
| **Age-adjusted all-cause mortality in males by Chinese ethnicity** | | | | | | | | |
| 3 | Serious | Not Serious | Not Serious | Not Serious | Undetected | **RR 0.66** (0.50 to 0.87) | ⨁⨁⨁◯ Moderate |  |
| **Age-adjusted all-cause mortality in females by Chinese ethnicity** | | | | | | | | |
| 3 | Serious | Not Serious | Not Serious | Not Serious | Undetected | **RR 0.64** (0.61 to 0.69) | ⨁⨁⨁◯ Moderate |  |
| **Question:** To what extent do inequalities in SEP account for observed differences in the relative risk of all-cause mortality in those of Chinese ethnicity?  **Age and SEP adjusted all-cause mortality in males by Chinese ethnicity** | | | | | | | | |
| 4 | Very Serious | Not Serious | Serious | Not Serious | Undetected | **RR 0.68** (0.55 to 0.84) | ⨁◯◯◯ Very low |  |
| **Age and SEP adjusted all-cause mortality in females by Chinese ethnicity** | | | | | | | | |
| 4 | Very Serious | Not Serious | Serious | Not Serious | Undetected | **RR 0.62** (0.52 to 0.75) | ⨁◯◯◯ Very low |  |
| **Question:** Does the relative risk of all-cause mortality in the Chinese population compared to the White British population differ by country of birth (UK-born vs born outside of the UK)?  **Age and sex adjusted all-cause mortality by Chinese ethnicity stratified by country of birth (UK vs non UK)** | | | | | | | | |
| 3 | Serious | Not Serious | Not Serious | Serious | Undetected | Chinese UK-born: **RR 0.82** (0.53 – 1.28)  Chinese non-UK born: **RR 0.69** (0.54 – 0.87) | ⨁⨁◯◯ Low |  |

**Question:** What is the relative-risk of all-cause mortality in those of Mixed ethnicity compared to White majority population?

**Setting:** United Kingdom

| **Certainty assessment** | | | | | | **Relative Effect (95% CI)** | **Certainty** | **Comments** |  |
| --- | --- | --- | --- | --- | --- | --- | --- | --- | --- |
| **№ of studies** | **Risk of bias** | **Inconsistency** | **Indirectness** | **Imprecision** | **Publication bias** |  |  |  |  |
| **Age-adjusted all-cause mortality in males by Mixed ethnicity** | | | | | | | | |  |
| 3 | Serious | Serious | Not Serious | Serious | Undetected | **RR 0.98** (0.88 to 1.10) | ⨁◯◯◯ Very low |  |  |
| **Age-adjusted all-cause mortality in females by Mixed ethnicity** | | | | | | | | |  |
| 3 | Serious | Not Serious | Not Serious | Serious | Undetected | **RR 0.94** (0.90 to 0.98) | ⨁⨁◯◯ Low | Serious risk for imprecision as although the confidence interval does not cross the line of no effect, it is unlikely that a RR of 0.98 (the upper end of the CI) indicates a difference in mortality of public health importance. |  |
|  | | | | | | | | |  |
| **Question:** To what extent do inequalities in SEP account for observed differences in the relative risk of all-cause mortality in those of Mixed ethnicity?  **Age and SEP adjusted all-cause mortality in males by Mixed ethnicity** | | | | | | | | |  |
| No information | | | | | | | | |  |
| **Age and SEP adjusted all-cause mortality in females by Mixed ethnicity** | | | | | | | | |  |
| No information | | | | | | | | |  |
| **Question:** Does the relative risk of all-cause mortality in the Mixed population compared to the White British population differ by country of birth (UK-born vs born outside of the UK)?  **Age and sex adjusted all-cause mortality by Mixed ethnicity stratified by country of birth (UK vs non UK)** | | | | | | | | | |
| 3 | | Serious | Serious | Not Serious | Serious | Undetected | Mixed UK-born: **RR 0.58** (0.18 – 1.82)  Mixed non-UK born: **RR 0.81** (0.58 – 1.13) | ⨁◯◯◯ Very low |  |

**Question:** What is the relative-risk of all-cause mortality in those of Polish ethnicity compared to White majority population?

**Setting:** United Kingdom

| **Certainty assessment** | | | | | | **Relative Effect (95% CI)** | **Certainty** | **Comments** | |  |  |
| --- | --- | --- | --- | --- | --- | --- | --- | --- | --- | --- | --- |
| **№ of studies** | **Risk of bias** | **Inconsistency** | **Indirectness** | **Imprecision** | **Publication bias** |  |  |  |  |  |  |
| **Age-adjusted all-cause mortality in males by Polish ethnicity** | | | | | | | | | |  |  |
| 4 | Very Serious | Serious | Not Serious | Serious | Undetected | **RR 1.09** (0.93 to 1.27) | ⨁◯◯◯ Very low |  | |  |  |
| **Age-adjusted all-cause mortality in females by Polish ethnicity** | | | | | | | | | |  |  |
| 4 | Very Serious | Serious | Not Serious | Serious | Undetected | **RR 0.96** (0.88 to 1.06) | ⨁◯◯◯ Very low |  | |  |  |
| **Question:** To what extent do inequalities in SEP account for observed differences in the relative risk of all-cause mortality in those of Polish ethnicity?  **Age and SEP adjusted all-cause mortality in males by Polish ethnicity** | | | | | | | | | |  |  |
| 4 | Very Serious | Not Serious | Serious | Not Serious | Undetected | **RR 0.86** (0.75 to 0.98) | ⨁◯◯◯ Very low |  | |  |  |
| **Age and SEP adjusted all-cause mortality in females by Polish ethnicity** | | | | | | | | | |  |  |
| 4 | Very Serious | Not Serious | Serious | Serious | Undetected | **RR 0.85** (0.65 to 1.11) | ⨁◯◯◯ Very low |  | |  |  |
| **Question:** Does the relative risk of all-cause mortality in the Polish population compared to the White British population differ by country of birth (UK-born vs born outside of the UK)?  **Age and sex adjusted all-cause mortality by Polish ethnicity stratified by country of birth (UK vs non UK)** | | | | | | | | | | | |
| No information | | | | | | | | | |  |  |

**Question:** What is the relative-risk of all-cause mortality in those of White Irish ethnicity compared to White majority population?

**Setting:** United Kingdom

| **Certainty assessment** | | | | | | **Relative Effect (95% CI)** | **Certainty** | **Comments** |
| --- | --- | --- | --- | --- | --- | --- | --- | --- |
| **№ of studies** | **Risk of bias** | **Inconsistency** | **Indirectness** | **Imprecision** | **Publication bias** |  |  |  |
| **Age-adjusted all-cause mortality in males by White Irish ethnicity** | | | | | | | | |
| 4 | Serious | Not Serious | Not Serious | Not Serious | Undetected | **RR 1.20** (1.06 to 1.34) | ⨁⨁⨁◯ Moderate |  |
| **Age-adjusted all-cause mortality in females by White Irish ethnicity** | | | | | | | | |
| 3 | Serious | Not Serious | Not Serious | Serious | Undetected | **RR 1.06** (0.87 to 1.29) | ⨁⨁◯◯ Low | No serious risk for inconsistency as differences in results were explained by the difference in comparison population (Scottish). |
| **Question:** To what extent do inequalities in SEP account for observed differences in the relative risk of all-cause mortality in those of White Irish ethnicity?  **Age and SEP adjusted all-cause mortality in males by White Irish ethnicity** | | | | | | | | |
| 7 | Very Serious | Serious | Serious | Not Serious | Undetected | **RR 1.16** (1.06 to 1.27) | ⨁◯◯◯ Very low |  |
| **Age and SEP adjusted all-cause mortality in females by White Irish ethnicity** | | | | | | | | |
| 5 | Very Serious | Not Serious | Serious | Serious | Undetected | **RR 1.08** (0.95 to 1.22) | ⨁◯◯◯ Very low |  |
| **Question:** Does the relative risk of all-cause mortality in the White Irish population compared to the White British population differ by country of birth (UK-born vs born outside of the UK)  **Age-adjusted mortality by White Irish ethnicity stratified by sex and country of birth (UK vs non-UK)** | | | | | | | | |
| 3 | Serious | Serious | Not Serious | Serious | Undetected | White Irish male UK-born: **RR 1.16** (1.00 – 1.33)  White Irish male non-UK born: **RR 1.05** (0.70 – 1.56)  White Irish female UK born: **RR 1.05** (0.85 – 1.31)  White Irish female non-UK born: **RR 1.14** (1.04 – 1.) | ⨁◯◯◯ Very low |  |

**Question:** What is the relative-risk of all-cause mortality in those of White Scottish ethnicity compared to White majority population?

**Setting:** United Kingdom

| **Certainty assessment** | | | | | | **Relative Effect (95% CI)** | **Certainty** | **Comments** | | |  |
| --- | --- | --- | --- | --- | --- | --- | --- | --- | --- | --- | --- |
| **№ of studies** | **Risk of bias** | **Inconsistency** | **Indirectness** | **Imprecision** | **Publication bias** |  |  |  |  |  |  |
| **Age-adjusted all-cause mortality in males by White Scottish ethnicity** | | | | | | | | | | |  |
| 6 | Serious | Not Serious | Not Serious | Not Serious | Undetected | **RR 1.19** (1.11 to 1.27) | ⨁⨁⨁◯ Moderate |  | | |  |
| **Age-adjusted all-cause mortality in females by White Scottish ethnicity** | | | | | | | | | | |  |
| 6 | Serious | Not Serious | Not Serious | Not Serious | Undetected | **RR 1.18** (1.08 to 1.29) | ⨁⨁⨁◯ Moderate |  | | |  |
| **Question:** To what extent do inequalities in SEP account for observed differences in the relative risk of all-cause mortality in those of White Scottish ethnicity?  **Age and SEP adjusted all-cause mortality in males by White Scottish ethnicity** | | | | | | | | | | |  |
| 2 | Very Serious | Not Serious | Serious | Not Serious | Undetected | **RR 1.31** (1.28 to 1.35) | ⨁◯◯◯ Very low |  | | |  |
| **Age and SEP adjusted all-cause mortality in females by White Scottish ethnicity** | | | | | | | | | | |  |
| No information | | | | | | | | | | | |
| **Question:** Does the relative risk of all-cause mortality in the White Scottish population compared to the White British population differ by country of birth (UK-born vs born outside of the UK)?  **Age and sex adjusted all-cause mortality by White Scottish ethnicity stratified by country of birth (UK vs non UK)** | | | | | | | | | | | |
| No information | | | | | | | | | |  |  |

**CI:** confidence interval; **RR:** risk ratio

# Supplement 18: Online repository

**Access online repository here:** [**https://osf.io/mt58h/files/osfstorage**](https://osf.io/mt58h/files/osfstorage)

Contains folders:

Comparison population sensitivity and subgroup analyses

Ethnicity measurement sensitivity and subgroup analyses

Other sensitivity and subgroup analyses

Post-hoc sensitivity and subgroup analyses

Publication bias

Study design sensitivity and subgroup analyses

Subgroup analyses

# Supplement 19: Analysis code

Data analysis was conducted using R (version 4.3.2; R Core Team, 2021).

The R code for the meta-analysis, which uses the ‘Metafor’ package, is available on GitHub: <https://github.com/linusyd/R-Script-for-Meta-Analysis/blob/main/R%20Script>.
